# Phase Engineering of Two‐Dimensional Transition Metal Dichalcogenides

**DOI:** 10.1002/smsc.202300093

**Published:** 2023-11-27

**Authors:** Jong Hun Kim, Hayeong Sung, Gwan-Hyoung Lee

**Affiliations:** ^1^ Department of Materials Science and Engineering Seoul National University Seoul 08826 Korea; ^2^ Department of Physics Inha University Incheon 22212 Republic of Korea

**Keywords:** phase engineering, phase patterning, polymorph, transition metal dichalcogenides, 2D materials

## Abstract

Since the successful isolation of single‐layer graphene with an atomic thickness, various van der Waals (vdW) materials have been intensively studied owing to their unique properties. Among the families of vdW materials, transition metal dichalcogenides (TMDs) have served as representatives because of their diverse band structures and intriguing quantum states, unlike those observed in their bulk counterparts. Particularly, unconventional polymorphic phases of TMDs increase the degrees of freedom in device fabrication and property modulation. As variations in structural phases significantly change the electrical, physical, and chemical properties of materials, phase engineering is essential for the new paradigm of TMD‐based devices. In this review, diverse strategies that can induce and control structural phases in TMDs are explored. After introducing the polymorphic phase changes and the resulting electronic band structures, the various empirical approaches used for manipulating phases in vdW materials, including phase‐selective synthesis and post‐synthesis treatments, are summarized. The group‐VI TMDs are considered as reference, and the analysis is extended to other TMDs across various groups in the periodic table. In addition to providing a comprehensive survey of the recent progress in TMD applications, the challenges for TMD applications and potential opportunities in emerging fields are discussed.

## Graphical abstract

This review critically explores strategies for inducing and controlling structural phases in vdW transition metal dichalcogenides (TMDs). It covers empirical methods like phase‐selective synthesis and postsynthesis treatments, focusing on group‐VI TMDs as a primary reference and extending the analysis to other TMDs. The review also addresses potential challenges and opportunities concerning TMD applications in emerging fields.

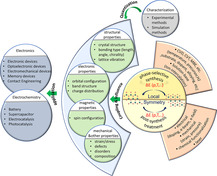

## 1 Introduction

Phase‐transition materials comprise several stable phases with repeated spatial ordering. These phases can be modified via external stimuli, such as heat, stress, and magnetic field. Typical phase transitions occur because of spontaneous symmetry breaking and can be understood in the context of thermodynamics.^[^ ^1^, ^2^, ^3^, ^4^ ^]^ In other words, if a high‐temperature system with low coupling loses energy and is cooled down to a critical temperature, it may transform into a lower symmetry state with higher coupling. As thermal fluctuation does not provide access to each energy state, more ordered states are preferred over entropy to eventually result in the new ground state. Therefore, the thermodynamic behavior of each phase can be characterized by an order parameter defined as a derivative of the partition function; this becomes discontinuous at the phase transition point. As an actual system contains numerous microstates equivalent to the Boltzmann number, the phenomenological Landau–Ginzburg approach is primarily used for examining the symmetries of the order parameter.^[^ ^5^ ^]^ Moreover, with the development of empirical research methods, the quantum phase transition has become an interesting topic apart from the Landau–Ginzburg approach.^[^ ^6^, ^7^ ^]^

Typically, as low‐dimensional materials, such as van der Waals (vdW) nanosheets, exhibit higher sensitivity to external stimuli than their bulk counterparts, a larger margin is generated for tailoring phases.^[^ ^7^, ^8^ ^]^ Therefore, controlled surface states in low‐dimensional materials facilitate the realization of thermodynamically unfavorable phases in bulk materials. Since the empirical discovery of single‐layer graphene,^[^ ^9^, ^10^, ^11^, ^12^, ^13^, ^14^ ^]^ manipulation of its surface properties, such as pressure, gas flow rate, growth temperature, and annealing protocols, using finely tuned growth conditions has been investigated.^[^ ^15^, ^16^, ^17^, ^18^, ^19^, ^20^, ^21^, ^22^, ^23^, ^24^, ^25^, ^26^ ^]^ Furthermore, as the symmetry breaking in graphene is easier than that in graphite, unconventional hidden phases are expected.^[^ ^27^ ^]^ For instance, although a bilayer hexagonal polytype exists as a natural unit cell in multilayer graphene, a trilayer can thermodynamically favor the rhombohedral structure under certain high‐energy environments. This leads to the spontaneous transition of phases into the rhombohedral structure via mechanical grinding^[^ ^28^ ^]^ and stacking .^[^ ^29^ ^]^ The window for phase transitions can also be enlarged by the relative orientation of stacked layers. Recent studies on the twisted bilayer graphene have reported that the dominant van Hove singularity‐driven flat band has resulted in the emergence of Mott‐insulating behavior and superconductivity owing to electronic correlations.^[^ ^30^, ^31^, ^32^ ^]^ The significant advances in graphene research have paved the way for investigating other vdW materials, such as transition metal dichalcogenides (TMDs). The group‐VI TMX_2_s are the representative TMDs, where M and X represent transition metals (Mo and W) and chalcogens (S, Se, and Te), respectively. Owing to its three‐atom‐layer structure (X‐M‐X), single‐layer TMDs can host more complex phase diagrams and exhibit enhanced physical properties in comparison with graphene. Particularly, tuning the interlayer interaction renders a multilayer TMD a promising nano‐template for investigating the relationships between crystal structure and properties. Therefore, TMDs have exhibited a wide range of unique properties^[^ ^33^, ^34^ ^]^ owing to their thickness‐dependent band structure,^[^ ^35^ ^]^ interlayer interactions,^[^ ^36^ ^]^ electron–electron coupling,^[^ ^37^, ^38^ ^]^ and electron–phonon coupling.^[^ ^39^, ^40^ ^]^

**Figure 1:**
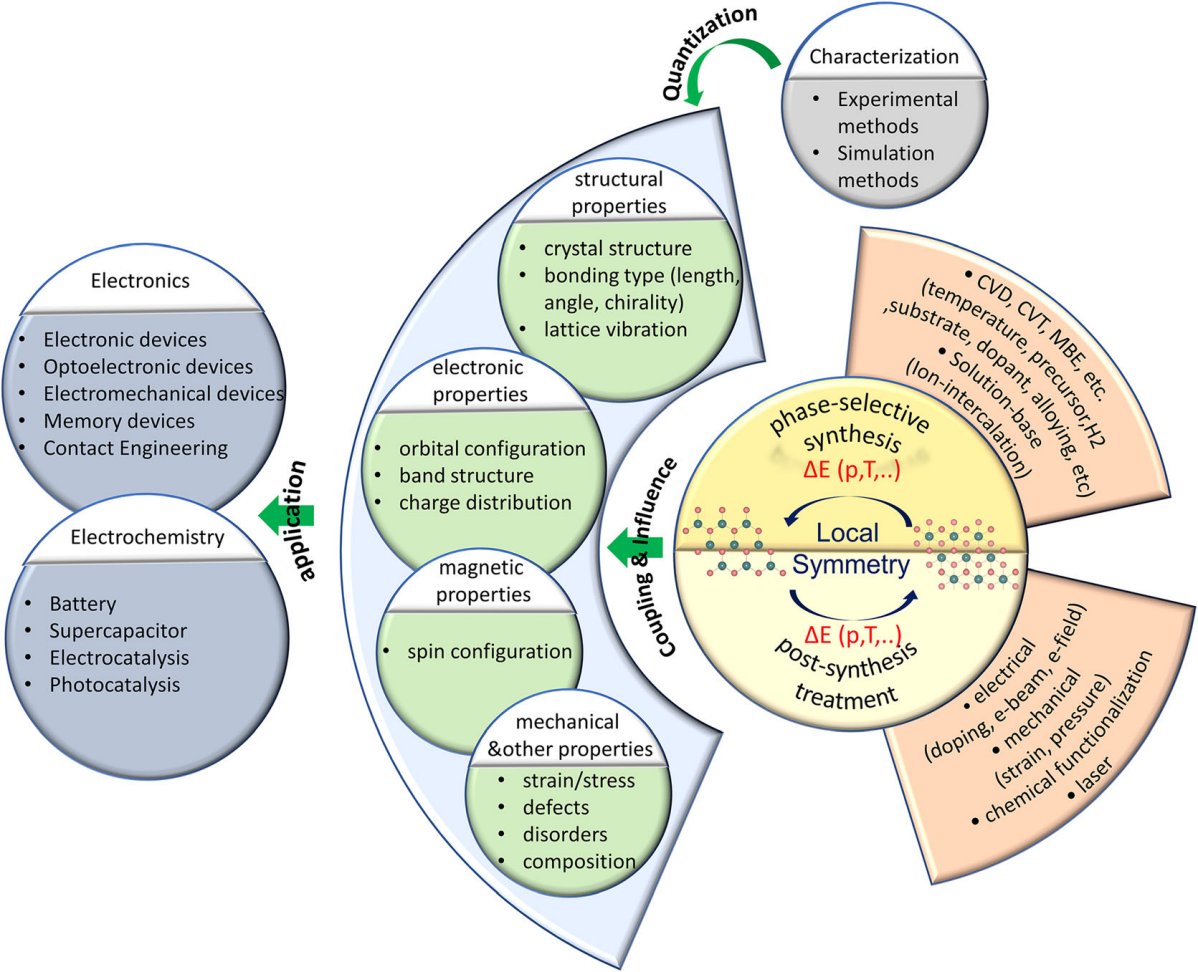
Schematic diagram outlining the overall flow of phase engineering in TMDs.

In this review (**Figure** **1**), we present an overview of phase transition in various TMDs. The discussion primarily begins with group‐VI TMDs, followed by a review of TMDs with other group transition metals, including Sn‐based dichalcogenides. Initially, we introduce the nomenclature used for denoting the different structural phase states in the phase engineering of TMDs. Subsequently, the concomitant electronic band structure is reviewed in conjunction with the corresponding structures. Furthermore, the concept of phase transition is briefly discussed from a thermodynamic perspective. We also summarize the various experimental tools used for monitoring the evolution of phases. The empirical phase engineering approaches for selectively mediating the specific phases of vdW materials are outlined, considering both the direct synthesis and post‐synthesis treatments. Additionally, the progress in other types of phase engineering, such as charge density wave (CDW) and superconductivity, is briefly reviewed. Finally, a survey of recent meaningful applications is presented along with the challenges and potential opportunities in emerging fields.

## 2 Polymorphic Phase Transition

### 2.1 Stack‐Dependent Polymorphic Transitions

**Figure 2:**
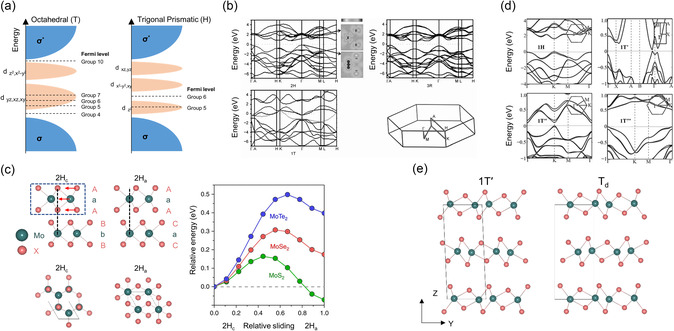
a) Schematic of the energy levels of 1T and 1H TMDs with the Fermi‐level positions obtained depending on the number of *d*‐electrons. b) Energy band structure calculation of 2H‐, 3R‐, and 1T‐MoS_2_ along the high‐symmetry points.^[^ ^51^ ^]^ Reproduced with permission.^[^ ^51^ ^]^ Copyright 2013, Springer Link. c) Structural details of 2H_c_‐ and 2H_a_‐MoX_2_ (left) and the total energy of MoX_2_ as a function of relative sliding from 2H_c_ to 2H_a_ (right).^[^ ^61^ ^]^ Reproduced under the terms of the CC BY license.^[^ ^61^ ^]^ Copyright 2015, The Authors, published by Springer Nature. d) Electronic band structures of various polymorphs of single‐layer MoS_2_ exhibiting the 2H/1H, 1T′, 1T″(2a × 2a), and 1T‴(√3a × √3a) structures.^[^ ^70^ ^]^ Reproduced under the terms of the CC BY license.^[^ ^70^ ^]^ Copyright 2016, The Authors, published by Springer Nature. e) Structural details of 1T′‐ and T_d_‐MoX_2_.

In contrast to graphene, TMDs exhibit various polymorphs and stacking fashions owing to their three‐atom‐layer structure. Therefore, the ability to derive polymorphic phases in vdW materials is the most essential and unique feature of TMDs. Additionally, in comparison to oxygen or halogen atoms, the relatively lower electronegativities of chalcogens result in discernible structural modifications.^[^ ^41^, ^42^ ^]^ The multilayer TMDs have repetitive unit layers of X‐TM‐X with weak vdW interlayer forces, wherein the intralayer is configured by combining covalent and ionic bonds between metal and chalcogen atoms within the layer. Consequently, multilayer TMDs commonly exhibit three possible stacking structures^[^ ^43^, ^44^ ^]^ (**Table** **1**), namely, 2H, 1T, and 3R; here, the letters in the nomenclatures denote, hexagonal, trigonal, and rhombohedral structures, respectively, and the numerical values indicate the number of X‐TM‐X atomic layers in a single unit cell. The stacking orders along the out‐of‐plane direction can be obtained as a series of three letters, each identifying the relative position between M and X atoms. Therefore, the atoms in the 1T, 2H, and 3R polymorphs are stacked in the AbC, AbA BaB, and AbA CaC BcB sequences belonging to different point groups of D_6d_, D_6h_, and C_3v_, respectively. Additionally, to differentiate a single‐layer trigonal TMD from 2H‐bilayer TMD, the former is often denoted as 1H, where the inversion symmetry is broken. As 2H/1H is thermodynamically more stable than other structures, bulk MoS_2_ generally exhibits the 2H/1H structure. Although the *d*‐orbital of TM is commonly positioned within the gap between the bonding (σ) and antibonding (σ*) states formed by TM–X, the electronic structure significantly depends on the coordination and number of *d*‐electrons. Five states of the *d*‐orbital are energetically divided into three levels (*d*_*z*_^2^; *d*_*x*_^2^_*–y*_^2^, *d*_*xy*_; and *d*_*xz*_, *d*_*yz*_) for the 2H/1H structure and two groups (*d*_*xy*_, *d*_*xz*_, *d*_*yz*_ and *d*_*x*_^2^_–*y*_^2^, *d*_z_^2^) for the 1T structure (**Figure** **2**a).^[^ ^41^, ^45^ ^]^ The number of *d*‐electrons in TMDs with the 2H/1H structure are zero and six for group‐IV 4 TMDs and six for group‐X TMDs, respectively. As the Fermi level is typically positioned within the energy gap between *d*_*z*_^*2*^ and *d*_*x*_^*2*^_*–y*_^*2*^, *d*_*xy*_ in the group‐VI TMDs with the 2H/1H structure, the TMDs act as semiconductors because of the occupied *d*‐orbitals.^[^ ^46^ ^]^ Furthermore, in the case of 1T‐TMDs, wherein the Fermi level within the partially occupied *d*‐states (*d*_*xy*_, *d*_*xz*_, and *d*_*yz*_) degenerates, TMDs predominantly exhibit metallic behaviors. As the atomic number of a chalcogen increases from S to Te, the bandgap gradually decreases owing to the increase in the valence band edge (VBE) caused by the increasing overlap of *p‐d* orbitals.^[^ ^47^, ^48^ ^]^ Conversely, the shift of the conduction band edge (CBE) is relatively small, indicating that its contribution to the band structure is marginal.^[^ ^41^ ^]^ As Mo attains stronger reactivity than W owing to the smaller size, the stability of Mo‐based TMDs is higher than that of W‐based TMDs.^[^ ^41^, ^49^ ^]^ Additionally, TMDs can alter their electronic band structures by varying the hybridization degree of *p‐d* orbitals using interlayer coupling. For instance, although the *d*‐orbitals of Mo in MoS_2_ are relatively insensitive to the thickness because of the sandwiched structure, the interlayer coupling involving S*p*_z_ orbitals can modify the electronic band structure depending on the thickness. Owing to the increased interlayer coupling, the slightly increased antibonding *p*_z_ state facilitates the VBE of 2H‐MoS_2_ to shift toward the Γ‐point, which in turn reduces the energy bandgap.^[^ ^50^ ^]^ The interlayer interaction in the 3R phase is fundamentally similar to that of the 2H phase, resulting in a similar electronic band structure; however, the exception is that the shift of the VBE from Γ to *A* is caused by the variations in the stacking order of the S‐Mo‐S layers.^[^ ^51^, ^52^, ^53^ ^]^ This behavior becomes significant for group‐V transition metal (Nb and Ta)‐based TMDs with the 3R polytype.^[^ ^54^, ^55^, ^56^, ^57^, ^58^ ^]^ Figure 2b depicts the band structures of 2H, 3R, and 1T phases. The 2H phase has an indirect bandgap, where VBE is positioned at Γ, and CBE is positioned between Γ and K. The 3R phase has an indirect bandgap similar to the 2H phase despite the different band structure.

**Table 1:** Polymorphisms in multilayer TMDs

2H	3R	1T′	T_d_
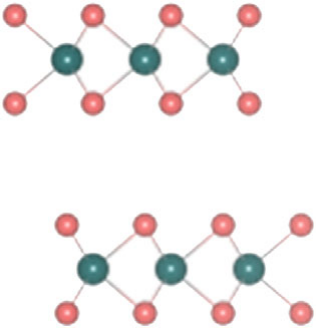	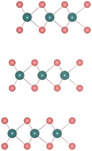	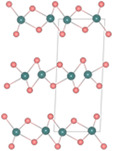	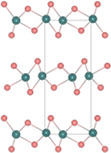
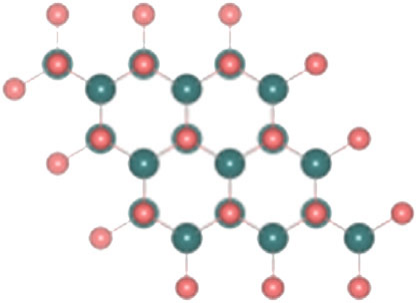	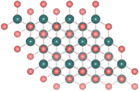	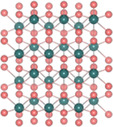	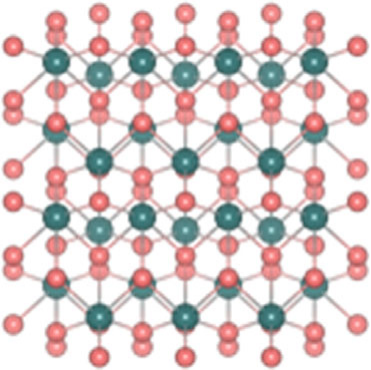

The 2H structure can be additionally divided into two stable states (Figure 2c), namely, AbA BaB (2H_a_) and AcA BaB (2H_c_). Here, the 2H_c_ state is equivalent to 2H and generally remains stable in group‐VI TMDs in the ambient condition. However, if the in‐plane (out‐of‐plane) lattice increases (decreases) owing to external pressure, the transition from 2H_c_ to 2H_a_ can occur by systematically shifting half of the atoms in a unit of X‐TM‐X. After the transition, their coordination is maintained during the subsequent sliding. In the case of Mo‐based TMX_2_, such phase transitions can appear with the gap closing, resulting in metallic properties.^[^ ^59^, ^60^ ^]^ However, as the energy barrier for the phase shift increases with the size of X, the transition to 2H_a_ under pressure has been primarily observed in MoS_2_ rather than in other Mo‐based TMX_2_s.^[^ ^61^, ^62^, ^63^ ^]^ This can be attributed to the Coulomb interaction between Mo and X in the 2H_c_ structure, which is reinforced by the shorter mutual distance under pressure when compared with the 2H_a_ phase. Therefore, in terms of the competing effect of vdW and coulomb forces, only MoS_2_ can possess a sufficiently low energy barrier and adequate stability in both phases, enabling reversible phase transition.^[^ ^64^ ^]^

### 2.2 Monolayer Polymorphic Transition

Owing to their energetically unstable state when compared with the 1H structure,^[^ ^65^, ^66^ ^]^ ideal octahedral 1T‐TMDs often undergo additional structural relaxation, resulting in the formation of 1T′, 1T″, and 1T‴ phases, where transition metals are superstructured in a zigzag‐chain (2a × a), a tetrarization (2a × 2a), and a trimerization (a√3 × a√3) arrangement on the *ab*‐plane (**Table** **2**).^[^ ^66^, ^67^, ^68^, ^69^ ^]^ Accordingly, 1T phases of MoS_2_ can transiently comprise nanodomains, including 1T″, 1T‴, and other transient phases.^[^ ^69^ ^]^ Zhuang et al.^[^ ^70^ ^]^ reported that the formation energies of 1T, 1T′, 1T″, and 1T‴ increase by 0.28, 0.18, 0.21, and 0.22 eV atom^−1^, respectively, with reference to 1H, implying that the distorted 1T phases are more stable than the 1T phases. Singh et al.^[^ ^71^ ^]^ also demonstrated that distorted 1T phases can be stabilized in group‐VI TMDs by providing additional energy, despite the 1T phase being energetically higher compared to the 1H phase. Furthermore, less stable derivatives of 1T may secure their sustainability by regulating the degree of doping, disorder, and thermal entropy.^[^ ^72^ ^]^ In terms of kinetics, the energy differences among 2H/1H, 1T, and 1T′ phases have been calculated for Mo‐ and W‐based TMDs ,^[^ ^70^, ^73^, ^74^ ^]^ and their transition dynamics (1H → 1T;^[^ ^69^, ^75^ ^]^ 1H → 1T → 1T′^[^ ^70^, ^73^, ^76^ ^]^) have been simulated and empirically observed, specifically for MoS_2_. Similar studies on phase transition have been conducted for other TMDs, primarily based on Mo^[^ ^66^, ^77^, ^78^, ^79^, ^80^ ^]^ or W.^[^ ^66^, ^68^, ^78^, ^81^, ^82^, ^83^ ^]^ As the distortions of 1T can break the degeneracy among *d*_*xy*_, *d*_*xz*_, and *d*_*yz*_, the energy levels of the occupied *d*_*xz*_ and *d*_*xy*_ bonding orbitals can be lowered. Furthermore, TMDs are susceptible to CDWs caused by various structural distortions, which can alter the material properties^[^ ^66^, ^84^ ^]^ and result in varying numbers of *d*‐electrons depending on the 1T derivatives.^[^ ^85^, ^86^, ^87^ ^]^ Figure 2d depicts the electronic band structures of various polymorphs of MoS_2_ monolayers, where VBE is set to zero. While the *E*_g_ value of 1H‐MoS_2_ is ≈1.9 eV, those of 1T′, 1T″, and 1T‴ superstructures are determined to be approximately 50, 140, and 570 meV, respectively.^[^ ^70^, ^88^ ^]^ Moreover, 1T′‐tellurized TMDs can undergo another structural transition to 1T_d_ phase at low temperatures by breaking the spatial inversion symmetry^[^ ^89^, ^90^, ^91^ ^]^ (Figure 2e). In the case of MoTe_2_, although 1T′ is a monoclinic structure with a slightly distorted angle (≈93°), *T*_d_ phases exhibit an orthorhombic structure with all three axes being orthogonal. Unlike in the case of the 2H structure, *T*_d_ exhibits metallic behavior similar to 1T′. However, in contrast to 1T′, T_d_ comprises a pair of Weyl cones along the Γ‐X direction while exhibiting a gap opening along the Γ‐S direction.^[^ ^90^, ^92^ ^]^ Additionally, studies have reported that the phase transition can be influenced either by temperature^[^ ^93^ ^]^ or the pressure.^[^ ^94^ ^]^ Several studies^[^ ^95^, ^96^ ^]^ have reported that the transition temperature can be increased above room temperature by reducing the thickness of the material because the energy of the hole band decreases more with the decreasing thickness.

**Table 2:** Polymorphisms in monolayer TMDs

1H	1T	1T′	1T″	1T‴
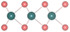	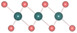	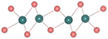	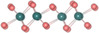	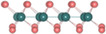
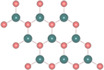	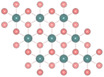	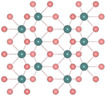	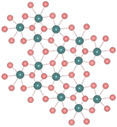	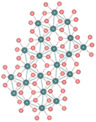

### 2.3 Basic Concept of Thermodynamics in Phase Transitions

In general, phase transition can be thermodynamically explained using the gradient of the free energy function (ΔG)^[^ ^1^, ^4^ ^]^ as follows

**Figure 3:**
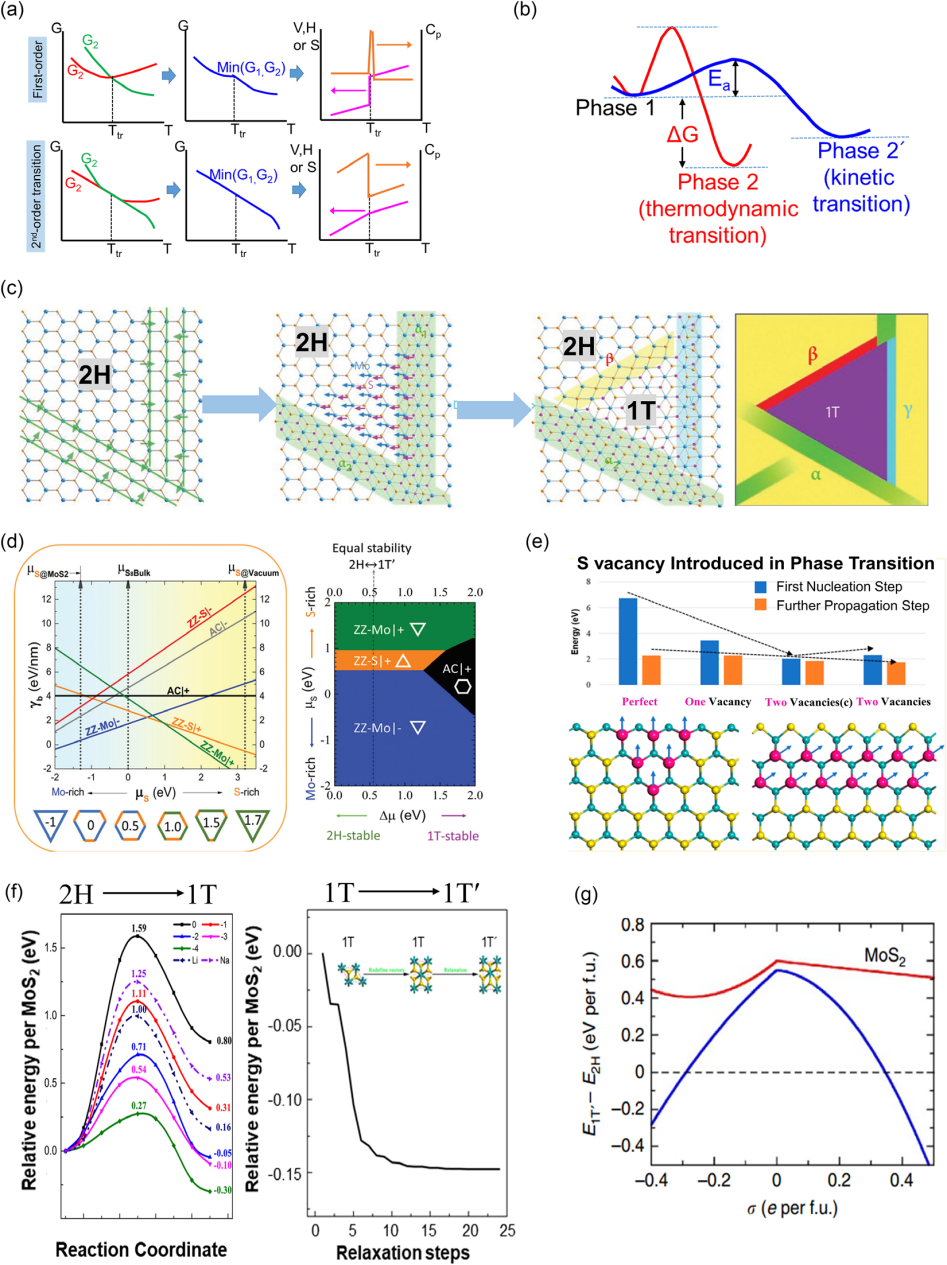
a) Variation of thermodynamic properties at the transition temperature (T_tr_) for first‐ and second‐order phase transitions. b) Phase determined between the thermodynamic and kinetic processes. c) Phase transformation kinetics from the trigonal prismatic structure (2H/1H) to an octahedral structure (1T/1T′) in single‐layer MoS_2_.^[^ ^75^ ^]^ Reproduced with permission.^[^ ^75^ ^]^ Copyright 2014, Springer Nature. d) The boundary energy as functions of the chemical potential of sulfur (left). Diagram of dominated boundaries under different chemical potentials (μS) and driving forces (Δμ). The vertical dashed line at Δμ = 0.55 eV represents equal stability of 2H and 1T′ phases.^[^ ^99^ ^]^ Reproduced with permission.^[^ ^99^ ^]^ Copyright 2017, RSC Pub. e) Sulfur vacancy introduced in the phase transition from 2H/1H to 1T in MoS_2_ nanosheets.^[^ ^101^ ^]^ Reproduced with permission.^[^ ^101^ ^]^ Copyright 2018, American Chemical Society. f) Minimum energy pathways of the phase transition from 2H to 1T in MoS_2_ under different charge/intercalation states (left). Spontaneous atom relaxation from 1T to 1T′ phases (right).^[^ ^65^ ^]^ Reproduced with permission.^[^ ^65^ ^]^ Copyright 2015, American Chemical Society. g) Phase boundary at a constant charge in single‐layer MoS_2._ charged beyond the positive or negative threshold values. MoS_2_ remains stable in the 2H structure when the charge is neutral.^[^ ^79^ ^]^ Reproduced under the terms of the CC BY license.^[^ ^79^ ^]^ Copyright 2016, The Authors, published by Springer Nature.

(1) ΔG=ΔU+pΔV–TΔS

where *U*, *p*, *V*, *T*, and *S* denote the internal energy, pressure, volume, temperature, and entropy, respectively. This indicates that a thermodynamically favored phase state exhibits the lowest energy under a specific condition, whereas other phases may remain unstable and transition into a more stable phase. The transitions can be categorized in terms of *n*, referring to the *n*th‐order derivative of free energy, with respect to *T* and *p* for a discontinuous transition (**Figure** **3**a). The first‐order transition indicates discontinuities in *V*, *S*, enthalpy (*H*), and heat capacity (*C*) at the transition points; here, *C* is defined using the derivative of *H* with respect to *T*. Furthermore, Δ*G*, *S*, and *V* of the systems are continuous in the second‐order transition, whereas only *C* exhibits discontinuity. As the total energy of nano‐ or low‐dimensional materials is affected by surface and interfacial factors more than that observed in bulk materials, the phase transition can be easily induced by modifying the activation energy barrier, the corresponding kinetics, and the thermodynamic potential^[^ ^7^, ^8^, ^97^, ^98^ ^]^ (Figure 3b).

As a representative example, this section briefly introduces the reconstruction process of a normal trigonal prismatic structure (2H/1H) to an octahedral structure (1T/1T′) in single‐layer MoS_2_. More details are presented in subsequent sections. The in situ transmission electron microscopy (TEM)^[^ ^75^ ^]^ indicates that Mo–Mo distance is locally compressed along the intermediate zigzag‐line while displacing the S‐atoms vertically, caused by the increase in temperature owing to the electron beam irradiation (Figure 3c). The author observed that the 2H/1T phase transition involves gliding atomic planes of S and/or Mo and requires an intermediate phase as a precursor. The formation of the intermediate state allows Mo atoms to align in zigzag chains rather than a trigonal configuration, causing local compressive strain in the Mo–Mo distance. The local strain energy induced by displacement is higher at the corners where the zigzag lines meet. Consequently, the built‐in energy should be released by triggering the gliding of the S‐plane to octahedral domains with low density without atomic loss. Zhao et al.^[^ ^99^ ^]^ predicted the thermodynamic stability and propagation behavior of each phase by calculating the formation energy for different interfacial boundary configurations that were deemed plausible. Indeed, the author theoretically demonstrated that the zigzag interface between the 2H and 1T (1T′) phases has the high energy barrier, favoring the triangular shape. The author also reported that the growth rate of the zigzag boundaries is kinetically much slower than the armchair ones due to the high energy barrier. Assuming that the phase transition tendency is derived from chemical potential as proposed by Cao et al.,^[^ ^100^ ^]^ the stability differences between distorted 1T and 2H phases were evaluated as the function of the chemical potential of sulfur and its gradient (Figure 3d). Among the suggested boundaries, S‐rich, Mo‐rich, and Mo‐deficient terminations along the zigzag direction were theoretically determined to exhibit higher stability in terms of formation energy compared with other configurations. Depending on the chemical potential of sulfur, the 1T domain in the 2H/1H phase was predicted to undergo successive changes in form, transitioning to triangular, truncated triangular, and hexagonal structures, in sequence. Additionally, they suggested that the nucleation of the 1T phase is unlikely to be initiated without defects in the site or edges owing to the high formation energy of ideal 2H/1H‐MoS_2_. Furthermore, Jin et al.^[^ ^101^ ^]^ clarified that the energy for the 1T phase is extremely high for it to occur in the ideal 2H/1H basal plane. However, the energy can be remarkably reduced by introducing either one or two S vacancies, which facilitates nucleation or propagation of 1T through the collective rotation of the three S atoms (Figure 3e).

Several researchers have reported theoretical studies on the phase transition induced by ions or electrons in single‐layer MoS_2_.^[^ ^65^, ^79^ ^]^ They suggested that the total energy of 2H/1H‐MoS_2_ becomes greater than that of 1T or distorted 1T phase by providing electrons to the conduction band, promoting the transformation. As stable 1T phases can be established by electron donation through ion intercalation, several studies have explored this topic^[^ ^65^, ^72^, ^102^, ^103^ ^]^ (Figure 3f). Particularly, Gao et al.^[^ ^65^ ^]^ reported that the energy required for transition systemically decreases from 1.59 to 0.27 eV when the injection charge per MoS_2_ unit increases from 0 to −4. The gradual decrease in energy can be associated with the electron‐induced two effects in MoS_2_. First, the entrance of excess electrons can weaken Mo–S bonding through the unequal distribution of electrons. Secondly, when electrons are injected, they preferentially split the *d*‐orbital into two sets: (partially occupied) *d*_*yz*_, _*xz*, *xy*_ and unoccupied $d_{z^{2}}$,$\;d_{x^{2}-y^{2}}$, exhibiting a metallic character. Therefore, the two effects can promote the phase transition from 2H to 1T′. Additionally, several studies have theoretically investigated whether a charge induced by an external electrostatic field can directly trigger a phase transition in MoS_2_.^[^ ^70^, ^79^ ^]^ This was experimentally realized for MoTe_2_ using the ionic liquid method as the phase stability in MoTe_2_ is lower than that of MoS_2_ or WS_2_. In electrostatic doping, injecting more electrons into the conduction band of the 2H phase results in a higher decrease in energy. Therefore, if the doping concentration is greater than the critical value, the total energy of 1T or other distorted 1T phases can be lower than that of the 2H/1H phase, indicating the feasibility of such phase transitions (Figure 3g).

### 2.4 Orthorhombic Pentagonal Structure

Unlike an isotropic planar honeycomb network, the recent study of black phosphorus has revealed that certain puckered structures can enhance the spin–orbit coupling or induce a topological quantum phase transition by breaking the substructure, resulting in the anomalous features such as the in‐plane anisotropic response to polarized light, electric field, or mechanical strain.^[^ ^104^, ^105^, ^106^ ^]^ Also, a new carbon polymorph called penta‐graphene was theoretically predicted to be thermodynamically and mechanically stable as well.^[^ ^107^ ^]^

vdW TMDs with a puckered pentagonal structure belong to another highly desirable class, primarily due to their low‐symmetry lattice structure. Most recently, TMDs based on Pd (PdX_2_) have been reported to possess a stable pentagonal layered structure, both theoretically^[^ ^108^, ^109^, ^110^, ^111^, ^112^, ^113^ ^]^ and empirically.^[^ ^114^, ^115^, ^116^, ^117^ ^]^ In this section, the common features of orthorhombic 2O‐TMDs will be briefly reviewed, with penta‐PdX_2_ serving as the most representative example.

**Figure 4:**
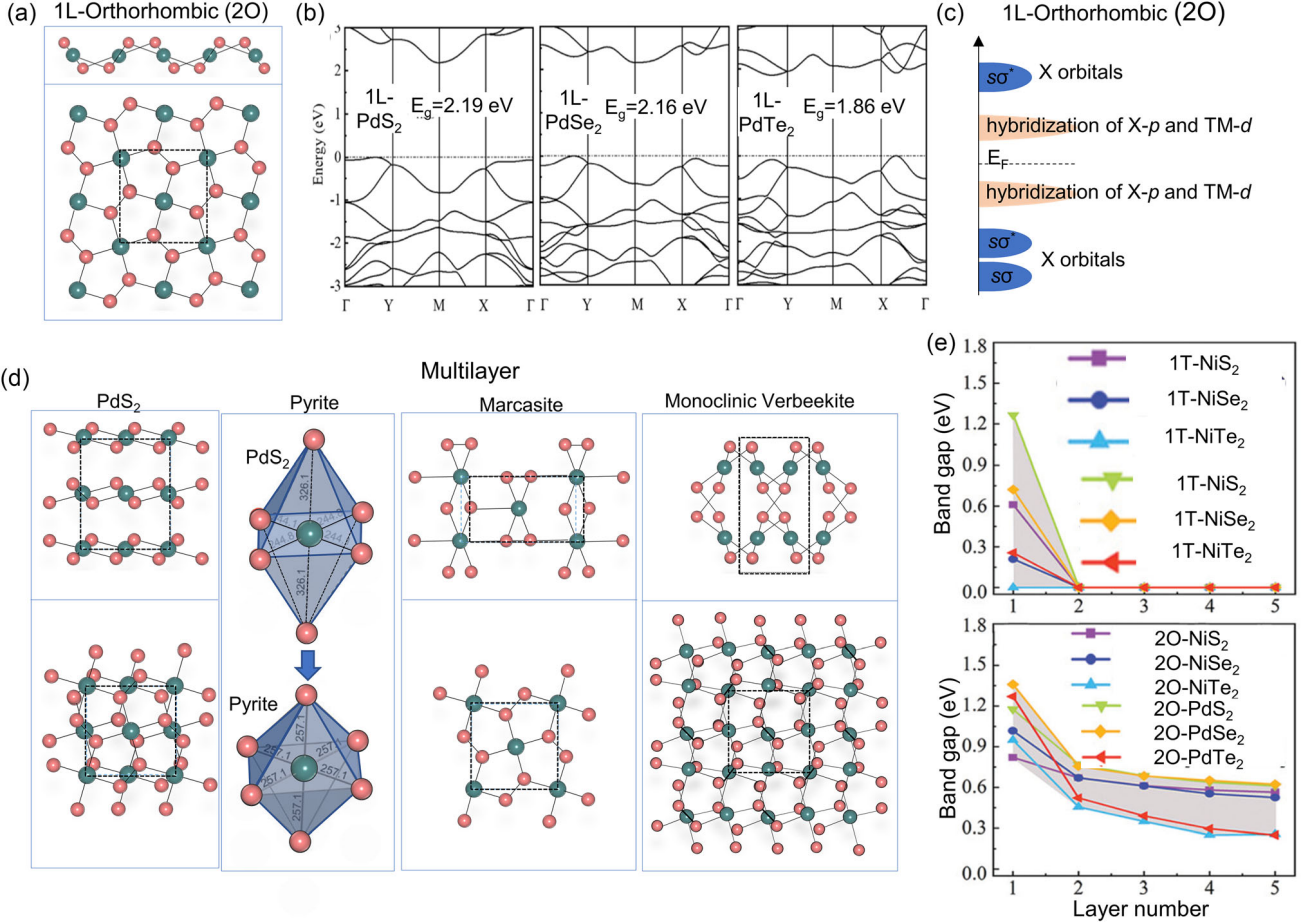
a) Top (bottom) and side (upper) view of a single‐layer PdX_2_ with puckered pentagonal structure. b) Band structure of the pentagonal 1L‐PdX_2_ ((left) PdS_2_, (middle) PdSe_2_, (right) PdTe_2_).^[^ ^113^ ^]^ Reproduced with permission.^[^ ^113^ ^]^ Copyright 2023, Elsevier Ltd. c) The schematic band structure of 2O‐pentagonal group‐X TMDs. d) Polymorphism observable in PdX_2_; the upper and bottom images in the first, third, and fourth column represent the top and side views of the corresponding phase, respectively. e) The layer number‐dependent bandgaps for (top) 1T‐TMX_2_, (bottom) 2O‐TMX_2_, respectively.^[^ ^111^ ^]^ Reproduced with permission.^[^ ^111^ ^]^ Copyright 2019, Royal Society of Chemistry.

**Figure** **4**a illustrates the schematic crystal structures of a single‐layer penta‐PdS_2_ where each Pd atom is linked to four S‐atoms, and that the adjacent S‐atoms are connected to each other to form an anisotropic pentagonal structure. Therefore, each pentagon possesses two Pd atoms and three X atoms. Due to the arrangement of two S atoms at the top and bottom planes of PdS_2_, a tilted dumbbell‐shaped (S_2_)^2−^ anion bonding structure intersects the Pd layer, while (PdS_4_)^2−^ structural units form a networked resembling a square‐planar configuration. As the result, PdX_2_ loses its rotational symmetry and belongs to an orthorhombic structure, known as 2O phase. The unit cell of the monolayer is slightly rectangular as shown in the dashed box as shown in Figure 4a. As suggested in Refs. [118, 119] it is natural for the lattice parameters of penta‐PdX_2_ to increase with the increase of X atoms. Due to the folding height being comparable to the thickness of a single atomic layer, the puckered structure can be considered as a sandwich of four‐coordinated TM atoms between X‐sublayers.^[^ ^120^ ^]^ Therefore, the side lengths and inner angles of the pentagon can be greatly influenced by the bonding strength between TM and X atoms, as well as between X and X atoms.^[^ ^111^, ^113^, ^118^, ^121^ ^]^ Both Lan et al.^[^ ^118^ ^]^ and Xiong et al.^[^ ^111^ ^]^ commonly have suggested a single‐layer penta‐PbX_2_ can remain stable at room temperature, on the basis of the phonon dispersion curves where phonon branch frequency in the Brillouin region exhibits almost no negative value regardless of types of X atoms, providing support for their thermal stability. In addition, Xie et al. have suggested the thermal stability of penta‐PdX_2_ and penta‐PtX_2_ by presenting the relatively high cohesive energy trend of PdS_2_(4.28 eV atom^−1^) > PdSe_2_(4.05 eV atom^−1^) > PdTe_2_(3.61 eV atom^−1^) that meet the thermodynamic stability. Qu et al.^[^ ^121^ ^]^ also have theoretically shown the thermal stabilities of various penta‐TMX_2_ including PdX_2_ by using ab initio molecular dynamics simulation. However, unlike PbS_2_ and PbSe_2_, the synthesis of the pentagonal structure in a single‐layer PdTe_2_ has been challenging so far, except in the high‐pressure environments.^[^ ^122^ ^]^ Feng et al.^[^ ^123^ ^]^ have exhibited that the energy difference between 1T and 2O‐phase in 1L‐PdX_2_ are quite smaller than other 1L‐PdX_2_. This is attributed to the less likely dimerization of Te–Te atoms due to their longer distance compared to other chalcogen atoms. However, since it is easier to shorten the interlayer Te–Te bonding compared to the intralayer Pd–Te bonding under high pressure, the observation of the pentagonal structure can be more feasible.^[^ ^122^ ^]^

Figure 4b displays the electronic band structure calculated for 1L‐penta‐PdX_2_. As indicated in the figure, all PdX_2_s have an indirect bandgap, which decreases in size with the increasing atomic number of a chalcogen atom. As the atomic number of the chalcogen atom increases, CBE shifts from point M to between Γ and X, while VBE is displaced from between Γ and X to between Y and Γ. Figure 4c demonstrates the relative position of CBE and VBM of PbX_2_. As shown in the schematic, the lowest energy band below E_F_ consists of the Se‐*s* states. The second lowest states below *E*_F_ mainly arise from the hybridization of the TM‐*d* and X‐*p* states, with the primary contribution from the TM‐*d* orbital. This raises the energy level in PdSe_2_ more than in PdS_2_. In comparison to sulfides and selenides, tellurides are more strongly hybridized with *d* orbital of TM due to their larger *p*‐orbitals. This results in a greater reduction in the bandgap, or even induces the metallization.^[^ ^124^, ^125^ ^]^ Similarly, the second highest region above E_F_ is also formed by the hybridization of TM *d*‐orbital and X‐ the *p*‐orbital. Finally, the highest energy state above *E*_F_ consists of the antibonding state, which is composed of the *s* and *p*‐orbitals of the X atom.^[^ ^113^, ^125^, ^126^ ^]^

Moving on to multilayer, if the layers of pentagonal TMX_2_ such as PdS_2_ or PdSe_2_ are stacked in a manner similar to the PdS_2_‐structure, it belongs to the distorted pyrite structure (Pbca), as illustrated in the first column of Figure 4d. The 2O‐PdS_2_‐type is similar to the pyrite structure (*Pa* $\bar{3}$), but lower the symmetry by elongating *c*‐axis of (TMX_6_)^4−^ octahedral to square‐planar coordination of TMX_4_, as illustrated at the top of the second column in Figure 4d. Besides, the orthorhombic pentagonal structure can be much more susceptible to the thickness and the stacking type than hexagonal one such as MoX_2_ or WX_2_ due to the completely occupied *d*‐orbitals of TM and the strong coupling between *p*_*z*_‐orbitals of interlayer chalcogen atoms. Therefore, the polymorphisms and properties in multilayer 2O‐TMX_2_ can be significantly modulated by the strong interlayer interaction and the relatively weaker covalent bond strength. To investigate the stabilities of each structure, Feng et al.^[^ ^123^ ^]^ systemically calculated the total energy of PdX_2_ in terms of stacking type and layer number. Based on the calculated lowest total energy, the author suggested that PdS_2_ maintain the PdS_2_‐like 2O‐phase irrespective of its layer number, while PdTe_2_ prefers 1T‐phsae. PdSe_2_ holds its phase from 2O in bulk down to bilayer, but tends to transition to the 1T phase in a single layer. Nevertheless, as suggested by Kempt et al. and Xiong et al.,^[^ ^111^, ^127^ ^]^ the stability of 2O‐PdTe_2_ has been discussed based on the results showing a negligible difference in oxidation state between 1T‐PdTe_2_ and 2O‐PdTe_2_. However, further study is still required to address the issue.

By increasing the energy of the system through pressure or temperature, the stacking type can be altered from 2O‐PdS_2_ to other pentagonal types. As shown in the second column of Figure 4d, the 2O‐PdS_2_ returns to the pyrite structure by reducing the interlayer under the high pressure and high temperature.^[^ ^116^, ^128^, ^129^, ^130^, ^131^ ^]^ Larchev et al.^[^ ^129^ ^]^ and Soulard et al.^[^ ^130^ ^]^ suggested that 2O‐PdS_2_‐type can relax back to Pyrite‐type by providing the energy of ≈38.6 kJ mol^−1^. The third column of Figure 4d shows another orthorhombic pentagonal network known as marcasite which is also obtainable through the dimerization of X atoms and the octahedrally coordinated TM atoms. Since these two phases differ mainly in the orientation of X‐dimers, the marcasite phase often competes with pyrite one, given their marginally small energy difference. Since marcasite is estimated to have a higher energy than 2O‐PdS_2_ type by ≈44.8 kJ mol^−1^ ,^[^ ^129^ ^]^ the energy difference between pyrite and marcasite is as small as 6.2 kJ mol^−1^, which often leads to competition between the two phases. Indeed, Larchev et al. observed the marcasite phase of PdSe_2_ at the environment of 7.5 GPa and 900 °C, whereas the pyrite‐phase was preferred above this temperature.^[^ ^127^, ^129^ ^]^ CoTe_2_^[^ ^132^ ^]^ and FeTe_2_^[^ ^133^ ^]^ are found as the orthorhombic marcasite‐like mineral, as well as in the 1T‐like layered structure. However, a strong interlayer Te–Te interaction causes a relatively small layer separation and a small c/a lattice ratio, significantly increasing the cleavage energies. Due to the strong interaction in marcasite crystal, achieving a single‐layer TMD through exfoliation is not feasible. Lastly, as illustrated in the fourth column of Figure 4d, 2O‐PdSe_2_ can undergo another evolution to monoclinic structure (called as *M*‐phase or verbeekite phase, *I2/a*), despite the scarce reports of direct synthesis on a substrate under ambient conditions.^[^ ^131^, ^134^ ^]^ Selb et al. first explored Verbeekite‐type PdSe_2_ and estimate that the energy of system is just a little higher by 1.4 kJ mol^−1^ in compared with 2O‐PdS_2_ type.

For the electronic structures of multilayer TMX_2_, Xiong et al.^[^ ^111^ ^]^ compared the layer number‐dependent trend in bandgap difference between 1T and 2O‐TMX_2_. In compared with the top of Figure 4e where the bandgap in the 1T‐phase rapidly decreases to zero with increasing thickness, the 2O‐pentagonal TMX_2_ exhibits a relatively mild reduction in bandgap (the bottom of Figure 4e). On the other hand, Feng et al.^[^ ^123^ ^]^ theoretically predicted that while 2O‐PdS_2_ and PdSe_2_, as well as 1T PdTe_2_ would exhibit semi‐metallic features in multilayer, 2O‐PdS_2_ and 1T‐PdSe_2_ in few layer would be semiconducting. Similarly, a few early theoretical computation results suggested that the bulk 2O‐PdS_2_ and 2O‐PdSe_2_ may be either (semi)metallic or have a tiny, small bandgap, but these have been attributed to the general underestimation of the bandgap and inappropriate description of interlayer interactions using PBE function in the DFT simulations.^[^ ^114^, ^123^ ^]^ Nonetheless, a reduction in bandgap or even closure was calculated with increasing layer number in the all PbX_2_ materials, as suggested in Xiong et al.^[^ ^111^ ^]^ Besides, unlike WS_2_ or MoS_2_, no shift from an indirect to a direct bandgap was observed. Kempt et al.^[^ ^127^ ^]^ reported that PdSe_2_ exhibits semiconducting properties in both the both pyrite and marcasite structures, while closing the bandgap under the high pressure. On the other hand, pyrite PdTe_2_ is reported to be metallic. Also, verbeekite PdSe_2_ is anticipated to have a direct bandgap as large as 0.85 eV.

## 3 Characterization Methods for Detecting Phase Transitions in TMD Nanosheets

### 3.1 High‐Angle Annular Dark‐Field (HAADF) TEM and Scanning TEM (STEM)

**Figure 5:**
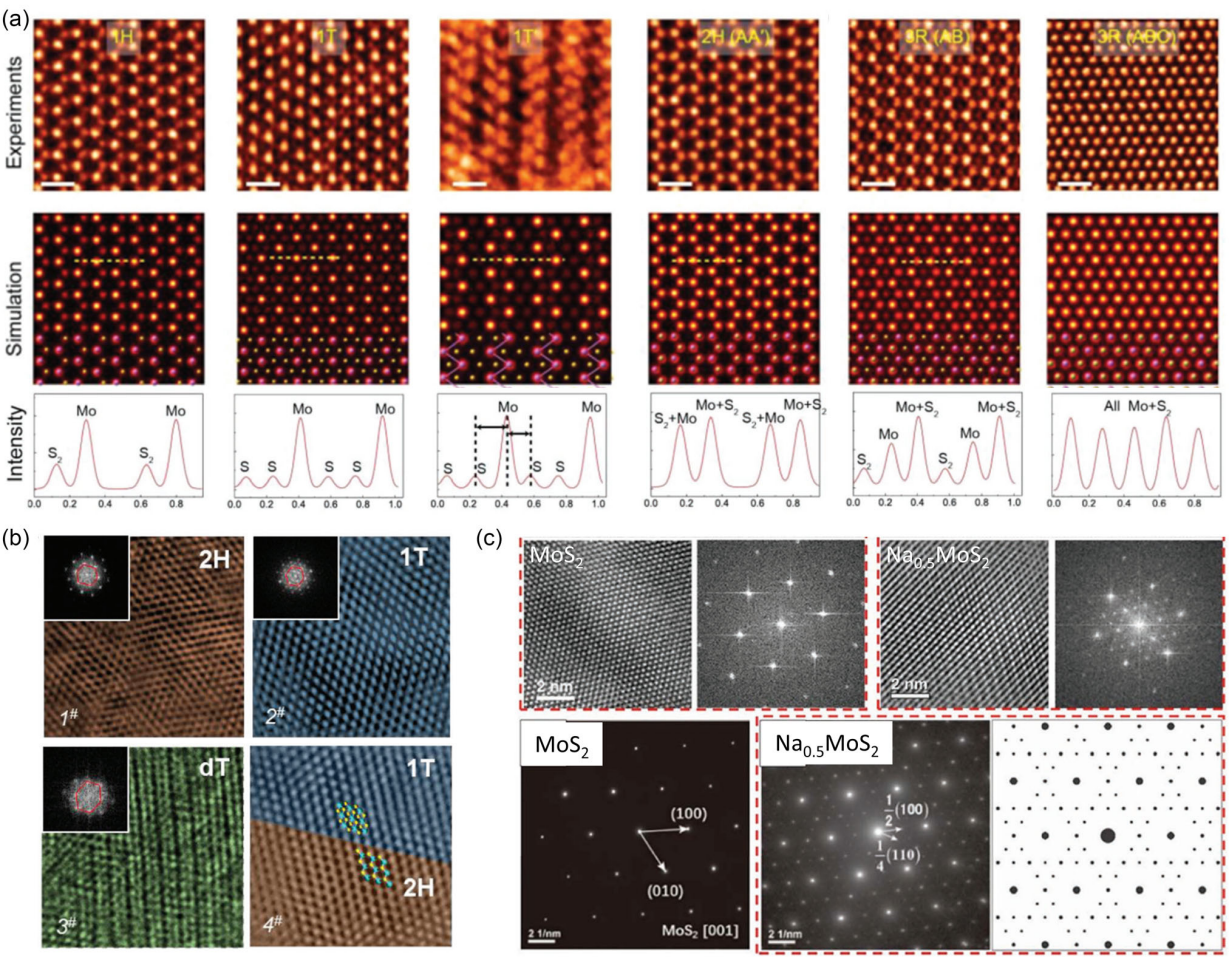
a) Atomic‐resolution ADF‐STEM images of a single layer of 1H, 1T, 1T′, bilayer 2H (AA′), bilayer 3R (AB)‐stacked, and trilayer 3R (ABC)‐stacked MoS_2_ (from left to right).^[^ ^145^ ^]^ Reproduced under the terms of the CC BY license.^[^ ^145^ ^]^ Copyright 2016, The Authors, published by Wiley‐VCH. b) High‐resolution TEM (HR‐TEM) images and FFT patterns of intercalated 2H‐MoS_2_ with different discharge potentials.^[^ ^143^ ^]^ Reproduced with permission.^[^ ^143^ ^]^ Copyright 2017, RSC Pub. c) HR‐TEM images and electron dispersion pattern of pristine 2H‐MoS_2_ and intermediate 2H‐Na_0.25_MoS_2_ phases.^[^ ^152^ ^]^ Reproduced with permission.^[^ ^152^ ^]^ Copyright 2017, Wiley‐VCH.

As phase transitions are characterized by a change in a macroscopic order parameter, such as conductivity or magnetization, knowing the individual components is essential to understand the different phases and mechanisms of phase transitions. Therefore, to analyze the structure of different phases, various electron‐beam‐based microscopy techniques have been developed, including TEM, high‐angle annular dark‐field (HAADF), and STEM. Additionally, as stacking faults can be associated with structural phase transitions, distinguishing between the structural phase shift and local defects is necessary. TEM presents an intuitive real‐space image at an ultrafine scale with adequate temporal resolution;^[^ ^135^, ^136^, ^137^ ^]^ therefore, it is often deemed the most convenient technique for monitoring morphological and structural variations.^[^ ^138^, ^139^, ^140^, ^141^, ^142^, ^143^, ^144^ ^]^ Furthermore, the improved resolution of HAADF combined with STEM renders it a popular tool for imaging vdW materials at the atomic scale. Zhao et al^[^ ^145^ ^]^ identified various polymorphs and stacking polytypes of MoS_2_ formed under different growth environments using HAADF‐STEM analysis with an appropriate image processing method. The authors reported that the microscopy images are consistent with the previously predicted simulations, indicating the reliability of TEM in studying the structures of vdW materials (**Figure** **5**a). Garcia et al.^[^ ^146^ ^]^ investigated the surface structures of MoS_2_ by varying the energy of an electron beam. They determined that the surface nearly remains intact at an electron beam voltage below 80 kV. This was ascertained by calculating the energy transfer from the electron beam to the surface and comparing it with the displacement threshold energies of Mo and S. As phase switching can be driven by perturbing vdW materials with electron beam irradiation during TEM analysis, various in situ TEM analyses have been performed to observe the method of altering the 2H/1H phase into 1T phase in the absence^[^ ^75^ ^]^ or presence^[^ ^147^, ^148^ ^]^ of defects. Consequently, the structural deformation observed by TEM is commonly interpreted as the macroscopic phase transition phenomenon that minimizes the accumulated strain energy caused by the structural distortions.

Despite the enhanced resolution of real‐space imaging, the contrast can be affected by sample thickness and objective lens aberrations, rendering accurate evaluations of atomic positions challenging and potentially inaccurate in multiple cases.^[^ ^149^, ^150^ ^]^ To address this issue, STEM is often performed using selected area electron diffraction (SAED) or fast Fourier transform (FFT) analyses, which can provide clearer and statistically significant information on the atomic structure.^[^ ^90^, ^141^, ^143^, ^151^, ^152^, ^153^, ^154^ ^]^ Xia et al.^[^ ^143^ ^]^ evaluated the electrochemical intercalation of Li ions into 2H‐MoS_2_ at different discharge and recharge potentials using TEM images in conjunction with FFT analysis. The authors revealed both the trajectory of the structural changes and the required threshold potential (Figure 5b). Huang et al.^[^ ^152^ ^]^ analogously investigated the sodium‐intercalation‐induced structural transformation of MoS_2_ using in situ TEM techniques, including FFT/SAED analysis (Figure 5c).

### 3.2 X‐Ray Diffraction (XRD)

**Figure 6:**
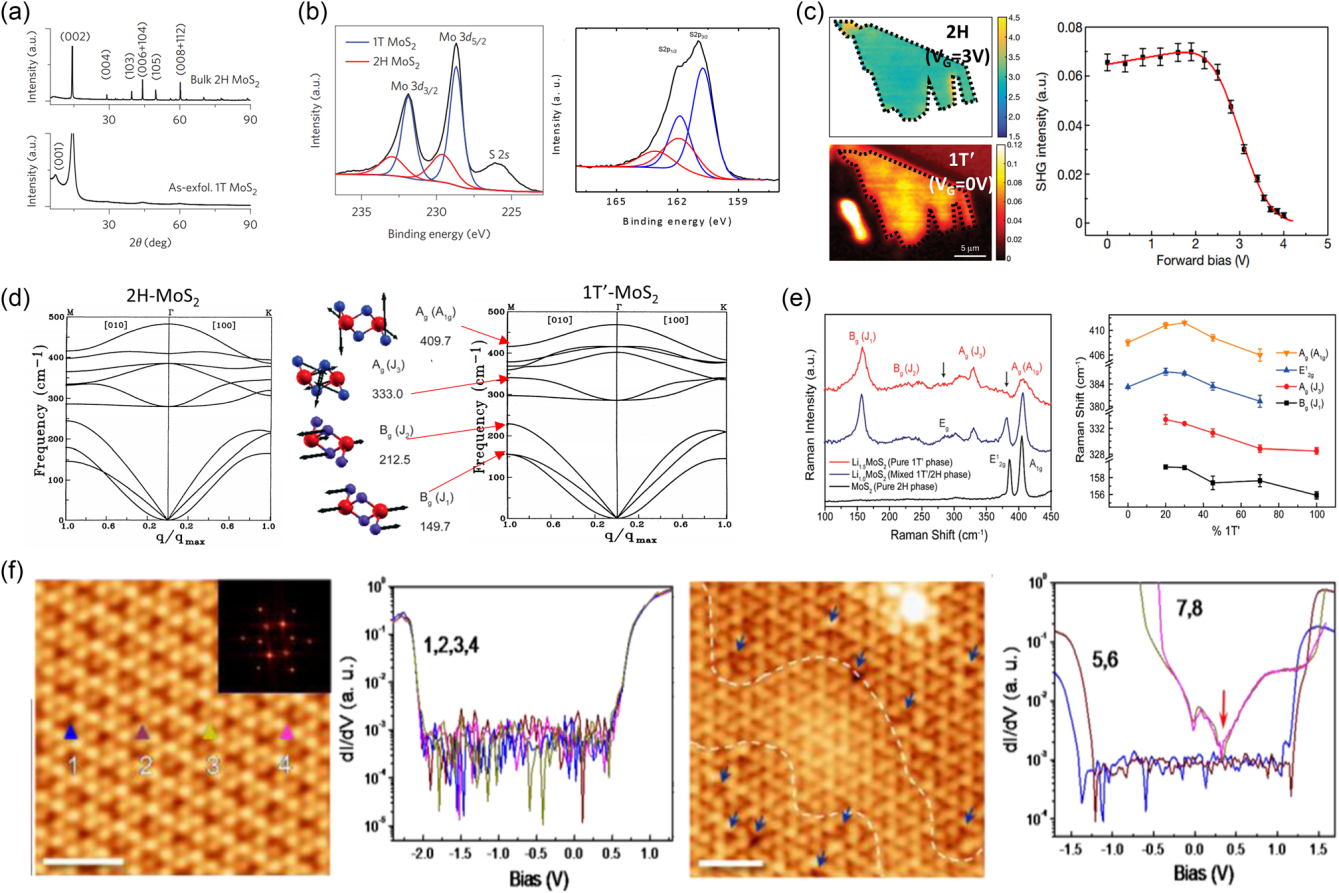
a) XRD of bulk MoS_2_ compared with as‐exfoliated MoS_2._^[^ ^155^ ^]^ b) X‐Ray photoelectron spectrum from (left) Mo3*d* and (right)S2*p* from the as‐exfoliated MoS_2._^[^ ^155^ ^]^ a,b) Reproduced with permission.^[^ ^155^ ^]^ Copyright 2015, Springer Nature. c) Spatial mapping of phase transitions in a MoTe_2_ single layer by SHG spectroscopy.^[^ ^168^ ^]^ Reproduced with permission.^[^ ^168^ ^]^ Copyright 2017, Springer Nature. d) Calculated phonon dispersion curves along the [010] and [100] directions of single layer (left)1H‐MoS and (right)1T‐MoS_2_. The schematic representations of the corresponding phonon modes 1T‐MoS_2_ are illustrated together.^[^ ^80^, ^175^ ^]^ Reproduced with permission.^[^ ^80^ ^]^ Copyright 1991, American Physical Society. e) The evolution of Raman modes with increasing lithium content, *x* in Li_*x*_MoS_2._^[^ ^175^ ^]^ d,e) Reproduced with permission.^[^ ^175^ ^]^ Copyright 2018, American Chemical Society. f) Atomic and electronic structure of local phase transition in single‐layer MoS_2_. Resolved by STM/STS before (left) and after (right) plasma treatment.^[^ ^181^ ^]^ Reproduced with permission.^[^ ^181^ ^]^ Copyright 2017, American Chemical Society.

X‐Ray diffraction (XRD) has traditionally been used as a straightforward and nondestructive tool for analyzing materials based on their crystal structure and atomic spacing using Bragg's law. Therefore, the characteristic peaks in the XRD spectrum represent the mutual distance between the atom layers, which in turn represents the crystallographic lattice. However, in contrast to their bulk counterparts, vdW materials often pose intrinsic challenges in probing their phase transitions owing to their small size and thinness, severely restricting the applicability of XRD to vdW nanosheets. Therefore, grazing‐incidence XRD (GIXRD) is considered a more suitable technique for characterizing the crystal structure of multiple microflakes or nanosheets of TMDs.^[^ ^139^, ^155^, ^156^, ^157^, ^158^ ^]^ According to Acerce et al.,^[^ ^155^ ^]^ the characteristic (002) peak obtained from restacked MoS_2_ decreases throughout the intercalation process, whereas the (001) peak newly appears, suggesting a larger interlayer expansion (**Figure** **6**a). Similarly, XRD analysis has shown that the interlayer spacing of MoS_2_ flakes can be expanded by adsorbing water molecules on both sides of the surface.^[^ ^139^ ^]^

### 3.3 X‐Ray Photoelectron Spectroscopy (XPS)

X‐Ray photoelectron spectroscopy (XPS) is a surface‐sensitive technique that can quantitatively determine chemical compositions, rendering it suitable for elucidating chemical surface configurations, such as compositions, doping, or surface bonding configurations in terms of electronegativity.^[^ ^139^, ^140^, ^155^, ^158^, ^159^ ^]^ For instance, with respect to semiconducting MoS_2_, the binding energies in both Mo‐ and S‐core levels in the metallic 1T‐MoS_2_ are shifted by ≈1 eV.^[^ ^140^, ^159^ ^]^ Furthermore, in situ XPS can be used to qualitatively analyze the method of inducing the evolution of the distorted 1T phase from the 2H/1H phase based on Li doping.^[^ ^160^ ^]^ However, XPS analysis exhibits certain challenges in discerning the differences among various 1T‐derivative phases. Similar to XRD, XPS can often suffer from the limited spatial resolution of the submillimeter scale despite its analytical accuracy, except for a few advanced cases.^[^ ^161^, ^162^ ^]^ Nevertheless, the thermal stabilities of each phase^[^ ^140^ ^]^ and surface oxidation states^[^ ^159^ ^]^ have been effectively evaluated by using XPS (Figure 6b).

### 3.4 Second‐Harmonic Generator (SHG)

SHG is a second‐order nonlinear optical process, which is particularly effective in detecting the inversion symmetry breaking of materials.^[^ ^163^ ^]^ As this type of symmetry breaking can be more prominent in vdW materials with fewer layers, SHG has been employed to investigate the layer thickness and stacking faults in various TMDs.^[^ ^164^, ^165^, ^166^ ^]^ Moreover, when combined with a pulse‐laser probe, SHG can be used as a more powerful characterization tool to probe the dynamics of symmetry variations as well.^[^ ^167^ ^]^ The inversion symmetry of TMDs can be broken owing to structural or stacking changes.^[^ ^164^, ^168^ ^]^ The phase transition in a MoTe_2_ transistor was reported to have been induced via electrostatic doping,^[^ ^168^ ^]^ accompanied by a change in symmetry (Figure 6c). As more electrons are inserted into the CBE, the lowest available energy states of 2H/1H are shifted by ≈100 meV, switching the ground state to the distorted 1T phase.^[^ ^79^ ^]^ Simultaneously, the SHG signal of 2H decreases owing to the centrosymmetry. Moreover, polarized SHG has been employed to examine the angular dependency of the signal and the difference between crystal phases.^[^ ^168^ ^]^ However, unlike MoTe_2_, detecting the phase transition in MoS_2_ using SHG has been slightly challenging owing to the higher stability of the 2H phase in MoS_2_.^[^ ^79^, ^169^ ^]^

### 3.5 Raman Spectroscopy

Raman spectroscopy is extensively employed to analyze the structural properties of vdW materials in both academic and industrial fields owing to its nondestructive nature and high spatial and spectral resolutions.^[^ ^170^, ^171^, ^172^ ^]^ In the case of TMX_2_, E^1^_2g_ and A_1g_ correspond to vibrational modes of the TM atom against the X atoms along the in‐plane and out‐of‐plane directions, respectively. In general, the frequencies of both E^1^_2g_ and A_1g_ modes exhibit a notable response to even minute structural changes, which can be attributed to the altered restoring force between the atoms.^[^ ^173^, ^174^ ^]^ Such pronounced sensitivity of phonon frequencies demonstrates that Raman spectroscopy can be a reliable tool for determining the number of layers in TMDs by varying the coupling forces dependent on the thickness. Similarly, the absence of frequency shifts in multilayer TMDs can be attributed to the interatomic forces that are saturated against thickness variation. Furthermore, phonon vibrations can be modified by different phases. According to Sandoval et al.,^[^ ^80^ ^]^ 1T‐MoS_2_ exhibits the emergence of additional Raman modes at ≈150 (J_1_), 226 (J_2_), and 333 cm^−1^(J_3_), whereas the characteristic Raman modes typically appear at ≈379 (E^1^_2g_) and 405 cm^−1^ (A_1g_) in 2H‐MoS_2_ (Figure 6d). Moreover, the authors determined that the observed modes were in agreement with the *M*‐points in the newly formed Brillouin zone of the 1T phase. Furthermore, Tan et al.^[^ ^175^ ^]^ reported that J_1_, J_3_, E^1^_2g_, and A_1g_ were softened in different manners with the increasing ratio of the distorted 1T phase to the 2H phase (Figure 6e). Additionally, Chen et al.^[^ ^176^ ^]^ explored the temperature‐dependent polarization configurations using polarized Raman spectroscopy to investigate the crystal phase transition driven by inversion‐symmetry at low temperatures.^[^ ^93^, ^176^ ^]^

### 3.6 Scanning Probe Microscopy (SPM)

In addition to electron‐beam‐based microscopy, various scanning probe microscopy (SPM) techniques,^[^ ^177^, ^178^, ^179^ ^]^ including scanning tunneling microscopy (STM) and atomic force microscopy (AFM), have been proposed as alternative real‐space imaging techniques to investigate the topography in conjunction with various order parameters, such as conductivity or magnetization at ultrafine spatial resolutions.^[^ ^68^, ^180^, ^181^, ^182^, ^183^ ^]^ As numerous two‐dimensional (2D) materials exhibit low stability, their characterization often relies on the ultrahigh vacuum (UHV)‐STM or UHV‐AFM. Furthermore, Kelvin probe force microscopy (KPFM), a derivative of AFM, has often been used to investigate electrostatic properties, such as surface potential, work functions, surface charge, and defect grain boundaries, on the surfaces of vdW nanosheets.^[^ ^184^, ^185^, ^186^ ^]^ The KPFM analysis performed by Precner et al.^[^ ^187^ ^]^ revealed that the work function of metastable states of MoS_2_ is governed by the thermally activated migration of lattice defects near the grain boundary and edges. Thus far, the possibility of phase transition has been investigated under various control factors, such as temperature,^[^ ^68^, ^187^ ^]^ electric field,^[^ ^188^ ^]^ electrochemical potential,^[^ ^77^, ^189^ ^]^ plasma,^[^ ^181^ ^]^ and adspecies,^[^ ^82^, ^182^ ^]^ using STM and AFM tests. Specifically, Zhu et al.^[^ ^181^ ^]^ developed a clean and efficient method for altering the phase in single‐layer MoS_2_ by using Ar‐plasma‐combined UHV‐STM along with scanning tunneling spectroscopy (STS) without exposing the sample to ambient conditions (Figure 6f). Furthermore, Valerius et al.^[^ ^190^ ^]^ investigated the surface state of single‐layer MoS_2_ based on in situ STM and STS, while subjecting the MoS_2_ to repetitive ion irradiation and annealing processes under UHV conditions. The aforementioned studies demonstrated that reversible structural phase transformations from crystalline to amorphous states can be achieved along with reversible conductivity switching between semiconducting and metallic properties. Moreover, the tip of an STM or AFM probe can locally perturb a sample, altering its phase or modifying its individual surface molecule and atoms. This has generated significant interest from both scientific and engineering perspectives as it enables manipulation of the long‐range ordered‐phase states.^[^ ^189^, ^191^, ^192^ ^]^

## 4 Various Families of TMDs

### 4.1 Non‐Group‐VI TMX_2_s

To date, researchers have investigated the hosting and triggering mechanisms of stable phases in various TMDs with respect to phase engineering. Several pioneering studies have systemically reported the available structural phases in various TMDs.^[^ ^43^, ^49^, ^193^, ^194^ ^]^ **Table** **3** summarizes the fundamental properties of different TMDs in terms of their structural and electrical aspects. As the electronic structure and final stable phase are significantly influenced by the atomic structure and *d*‐configuration of TM, TMX_2_s are categorized by the group number of the periodic table corresponding to TM.

**Table 3:** TMD group, phase, method, property

Group	[M]	TMD	Phase	Synthesis method	Properties	Remarks	reference
4	Ti	TiS_2_	1T	CVD	Semimetal		[598]
		TiSe_2_	1T	CVT	Semimetal	CDW	[599]
		TiTe_2_	1T	MBE, mechanical exfoliation(purchased)	Semimetal	CDW	[600]
	Zr	ZrS_2_	1T	CVD	Semiconductor	n‐type, Bandgap(1 L: 2 eV, bulk: 1.7 eV)	[601]
		ZrSe_2_	1T	CVT	Semiconductor	n‐type, Bandgap(1 L, bulk: 0.9–1.2 eV)	[602]
		ZrTe_2_	1T	MBE	Semimetal		[603]
	Hf	HfS_2_	1T	CVD	Semiconductor	n‐type, Bandgap(1 L: 2.67 eV, bulk: 2.0 eV)	[604]
		HfSe_2_	1T	CVT	Semiconductor	n‐type, Bandgap(1 L, bulk: 0.9–1.2 eV)	[602]
		HfTe_2_	1T	MBE	Semimetal		[605]
5	V	VS_2_	2H	CVD	Semiconductor	*p*‐type, Bandgap(0.187 eV)	[606]
			1T	CVD	Semimetal	semimetal	[607]
		VSe_2_	2H	CVD	Semiconductor	*p*‐type, Bandgap(0.19 eV)	[608]
			1T	CVD	Metal	CDW	[609]
		VTe_2_	1T, 1T′	MBE	Metal	magnetism, CDW	[610]
	Nb	NbS_2_	3R	CVD	Metal		[611]
		NbSe_2_	2H	CVD	Metal	CDW, superconductor	[612]
		NbTe_2_	1T″	CVD	Metal	CDW, superconductor	CVD^[^ ^613^ ^]^ STM analysis^[^ ^614^ ^]^
	Ta	TaS_2_	1T	CVD	Metal	CDW, superconductor	[615]
		TaSe_2_	2H, 1T	CVD	Metal	CDW, superconductor	[312]
		TaTe_2_	1T′ (arguable)	CVT	Semimetal	CDW	[616]
6	Cr	CrS_2_	2H, 1T, 1T′	CVD	Semiconductor	1T n‐type, mixed phase *p*‐type, magnetism	Mixed phase^[^ ^617^ ^]^ Pure 1T^[^ ^618^ ^]^
		CrSe_2_	1T	CVD	Metal	magnetism	[619]
		CrTe_2_	1T	CVD	Metal	magnetism	[620]
	Mo	MoS_2_	2H	CVD	Semiconductor	n‐type, Bandgap(1 L: 1.8 eV, bulk: 1.29 eV)	[621]
			1T′	CVD	Metal		[102]
		MoSe_2_	2H	CVD	Semiconductor	n‐type, Bandgap(1 L: 1.57 eV, bulk: 0.85 eV)	[622]
			1T	solvothermal synthesis	Metal		[623]
		MoTe_2_	2H	CVD	Semiconductor	ambipolar, Bandgap(1 L: 1.2 eV, bulk 1.0 eV)	[265]
			1T	CVD	Semimetal		[265]
			1T'	CVD	Metal		[265]
			Td	encapsulated annealing with hBN	Metal		[379]
	W	WS_2_	2H	OHVPD	Semiconductor	n‐type, Bandgap(1 L: 2 eV, bulk: 1.4 eV)	[624]
		WS_2_	1T	hydrothermal synthesis	Metal		[625]
		WSe_2_	2H	PVD	Semiconductor	*p*‐type, Bandgap(1 L: 1.65 eV, bulk: 1.2 eV)	[626]
		WSe_2_	1T'	solution‐phase synthesis	Metal		[267]
		WTe_2_	2H	lithium‐intercalation‐assisted exfoliation	Semiconductor	Bandgap(2.08 eV)	[286]
		WTe_2_	1T'	MBE	Semimetal	1 L Quantum Spin Hall insulator, bulk semimetal	[627, 628]
		WTe_2_	Td	mechanical exfoliation(purchased)	Semimetal	type‐II Weyl semimetal	[629]
7	Tc	TcS_2_	1T”	First‐principles calculations	Semiconductor	*p*‐type, Bandgap (1 L: 1.87 eV, bulk: 1.19 eV)	[630]
		TcSe_2_	1T″	First‐principles calculations	Semiconductor	*p*‐type, Bandgap(1 L: 1.64 eV, bulk: 1.01 eV)	[630]
		TcTe_2_	1T″	First‐principles calculations	Semiconductor	n‐type, Bandgap(1 L: 1.21 eV), *p*‐type, Bandgap(bulk: 0.37 eV)	[630]
	Re	ReS_2_	1T″	CVD	Semiconductor	n‐type, Bandgap(1 L: 1.61 eV, bulk: 1.48 eV)	[631]
		ReSe_2_	1T″	CVD	Semiconductor	*p*‐type, Bandgap(1 L: 1.32 eV, bulk: 1.26 eV)	[632]
8	Fe	FeSe_2_	Hexagonal	CVD	Metal		[231]
		FeTe_2_	Hexagonal	CVD	Metal	intrinsic h‐FeTe_2_ is metal, but n‐type semiconductor behavior due to the presence of discontinuity and/or disorder	[633]
9	Ni	NiS_2_	1T	CVD	Metal		[634]
		NiSe_2_	1T	Selenization of the Ni substrate	Metal		[249]
		NiTe_2_	1T	CVD	Metal		[251]
	Pd	PdS_2_	1T	PE‐CVD	Semiconductor(1 L), semimetal(bulk)	Bandgap(1 L: 1.28 eV, bulk: semimetal)	[246]
		PdSe_2_	1T	Self‐flux method and mechanical exfoliation	Semiconductor(1 L), semimetal(bulk)	Bandgap(1 L: 1.3 eV, bulk: semimetal)	[114]
		PdTe_2_	1T	MBE	narrow gap‐Semiconductor(1 L), Semimetal (bulk)	Bandgap(1 L: 0.14 eV, bulk: semimetal)	[635]
	Pt	PtS_2_	1T	CVT	Semiconductor	Bandgap(1 L: 1.6 eV, bulk: 0.25 eV)	[512]
		PtSe_2_	1T	Mechanical exfoliation(purchased)	Semiconductor(1 L), Semimetal(bulk)	Bandgap(1 L: 1.2 eV, bulk: semimetal)	[514]
		PtTe_2_	1T	Solid‐phase synthesis	Semiconductor(1 L), Semimetal(bulk)	Bandgap(1 L: 0.37, bulk: semimetal)	[514]
14 (IVA)	Ge	GeS_2_	T_d_ (orthorhombic)	Solvothermal process, CVT	Semiconductor	*p*‐type, Bandgap(2.8–3.4 eV (direct))	[636, 637]
		GeSe_2_	1T (monoclinic)	CVD	Semiconductor	*p*‐type, Bandgap(≈2.8 eV)	[638]
		GeTe_2_	Tetragonal	CVT	Semiconductor, metal (1 L)	metallic (1 L), Bandgap(≈1.1 eV)	[639]
	Sn	SnS_2_	2H	CVT	Semiconductor	*p*‐type, Bandgap(≈2.2 eV (indirect))	[257]
		SnSe_2_	2H	CVT	Semiconductor	n‐type, Bandgap(≈1.0 eV (indirect))	[640]
		SnTe_2_	1T	CVT	Metal	metallic	[331, 641]
Alloy	Ternary	MoS_2(1−*x*)_Se_2*x*_	2H	CVD	Semiconductor	*p*‐type, Bandgap(≈1.5–1.8 eV (with 0 < *x* < 1))	[642, 643]
		Mo_(1−*x*)_W_*x*_S_2_	2H	CVD	Semiconductor	n‐type, Bandgap(≈1.9–2.0 eV (with 0 < *x* < 1))	[329, 644]
		Mo_1−*x*_W_*x*_Se_2_	2H	CVT	Semiconductor	n‐type, Bandgap(≈1.56–1.65 eV (with 0 < *x* < 1))	[645]
		WS_2(1−*x*)_Se_2*x*_	2H	CVD	Semiconductor	*p*‐type, Bandgap(≈1.6–2.0 eV (with 0 < *x* < 1))	[646, 647]
		MoS_2−*x*_Te_*x*_	2H + 1T′	CVD	Semiconductor	*p*‐type. Bandgap (≈1.35–1.85 eV (with 0 < *x* < 0.3))	[648, 649]
		MoSe_2−*x*_Te_*x*_	2H, 1T′, T_d_	CVD	Semiconductor	*p*‐type, Bandgap (≈1.38–1.55 3 eV (with 0 < *x* < 2))	[337, 650]
		WS_2(1−*x*)_Te_2*x*_	2H + 1T′	CVD	Semiconductor/metallic	*p*‐type, Bandgap (≈1.67–1.97 eV (*x* < 0.5)) metallic (*x* > 0.5)	[332]
	Quaternary	Mo_*x*_W_(1−*x*)_S_2*y*_Se_2(1−*y*)_	2H	CVD	Semiconductor	*p*‐type, Bandgap (≈1.6–1.85 eV (with 0 < *x* < 1))	[343]

Group‐VI TMX_2_s with empty *d*‐orbital (*d* ^0^) exhibits 1T structure, where Fermi level is close to or even above CBE. Such a strong n‐type or degenerated state is likely a consequence of doping, resulting from the deficiency in X atoms or excess concentration of TM. Lucovsky et al.^[^ ^195^ ^]^ empirically verified that group‐4 (Ti, Zr, and Hf)‐based TMX_2_s can be identified as the 1T phase. Ti‐based TMX_2_s are metallic owing to the significant overlapping between *p*‐orbitals of X and *d*‐orbitals of TM, whereas Zr‐ or Hf‐based TMX_2_s reveal the semiconducting properties for X = S and Se despite their 1T structure.

Although group‐5 TMX_2_s with a *d* ^1^ configuration prefer the 1T phase over the 2H phase, the energy difference between the two phases reduces, generally in the order of a few tens of mV, owing to the increased stability of the 2H phase. This phenomenon enables the coexistence of both phases.^[^ ^196^, ^197^ ^]^ However, regardless of the structural phase, most group‐5 TMX_2_s exhibit metallic behavior. While Nb‐ and Ta‐based TMX_2_s are commonly reported to be metallic even in free‐standing environments,^[^ ^43^, ^47^, ^48^, ^192^, ^198^, ^199^ ^]^ V‐based TMX_2_s often exhibit the metal–insulator transition with a concomitant structural change as observed in 1H‐VS_2_^[^ ^200^, ^201^ ^]^ or 1H‐VSe_2_.^[^ ^202^ ^]^ In contrast to the metallic 1T phase in bulk VS_2_ or VSe_2_, the energy of 1H in a single layer slightly decreases when compared to that of 1T, leading to a phase transition to 1H. This transition opens the bandgap owing to the weakening intraband coupling in the single layer.^[^ ^201^, ^203^, ^204^ ^]^

Group‐VII TMX_2_s with *d* ^3^ electron configuration generally exhibit a distorted 1T structure, as demonstrated by Re‐^[^ ^205^, ^206^, ^207^, ^208^, ^209^, ^210^ ^]^ and Tc‐based^[^ ^211^, ^212^, ^213^, ^214^ ^]^ TMX_2_s, which facilitates semiconducting behavior. Re‐based TMX_2_s exhibit a bandgap that is similar to the direct bandgap, regardless of the layer thickness, which is advantageous for optoelectronic applications.^[^ ^215^ ^]^ Particularly, ReS_2_^[^ ^210^, ^216^ ^]^ and ReSe_2_^[^ ^217^ ^]^ are known for their pronounced in‐plane anisotropy resulting from the bulked chalcogenide layer and zigzag Re‐chain within the plane.^[^ ^210^ ^]^ Moreover, they have been reported to exhibit other stable phases, such as a low‐symmetry distorted 3R CdCl structure with a moderate semiconducting bandgap, under ambient conditions.^[^ ^218^ ^]^ Chen et al.^[^ ^219^ ^]^ reported that the 1T″ phase can remain stable in Re‐based TMX_2_s, resulting in anisotropic crack behavior at the edge. The study further empirically realized that the strain at the crack edges can reset the 1T″‐lattice structure, thereby pivoting the anisotropic behavior of ReX_2_ via strain‐induced effects.

Unlike other group TMX_2_s, group‐8 TMX_2_s exhibits a different type of polymorphism as observed in Fe‐based TMX_2_s, which tend to crystallize either in the marcasite structure (*Pnnm*) or pyrite structure (*Pa* $\bar{3}$), depending on subtle variations in the chemical composition ratio, pressure, and temperature.^[^ ^220^, ^221^, ^222^, ^223^ ^]^ Despite their similar structures, the octahedra are elongated in the *ab*‐plane in the former, whereas they are regular in the latter. Additionally, the pyrite lattice structure is characterized by a center‐facing TM atom, which is octahedrally coordinated by six X atoms; however, marcasite is composed of X‐atom chains that share their edges. Although both marcasite and pyrite structures exhibit certain structural differences, they commonly possess an indirect bandgap^[^ ^220^, ^224^, ^225^ ^]^ that narrows with the increasing atomic period of X.^[^ ^226^ ^]^ However, as the cleaving energy tends to increase in the order of (111) < (100) < (110) in the structures, the (111) plane with the least energy is considered the predominant facet,^[^ ^227^, ^228^ ^]^ significantly restricting the exfoliation unlike that observed in conventional vdW materials. Beiranvand et al.^[^ ^229^ ^]^ predicted that if FeSe_2_ adopts a hexagonal 1T structure in a single‐layer form, it would exhibit metallic properties owing to the hybridization between Fe‐*d* and Se‐*p* orbitals. Moreover, since Zhang et al. suggested the possibility of vdW Fe‐based TMX_2_s,^[^ ^230^ ^]^ there has been a renewed interest in synthesizing their nanosheets. Subsequently, Liu et al.^[^ ^231^ ^]^ reported the synthesis of multilayer vdW FeSe_2_ using chemical vapor deposition (CVD), exhibiting its highly conductive metallic properties. Nair et al.^[^ ^232^ ^]^ recently reported 2D ferromagnetic metal properties by synthesizing few‐layer 1T‐FeS_2_ nanosheets.

Unlike group‐VIII TMX_2_s, group‐9 TMX_2_s based on Ir or Co with *d* ^5^ configuration do not tend to form marcasite structures; they prefer to exist as vdW‐layered TMDs with strong interlayer contacts.^[^ ^132^ ^]^ Therefore, a direct growth approach was employed to synthesize TMX_2_ nanosheets as an alternative to the exfoliation method. Recently, Ma et al. synthesized well‐oriented CoTe_2_ nanosheets with a tunable thickness, wherein the orientation is normal to the [001] direction. These nanosheets adopted a hexagonal 1T phase with favorable metallic conductivity.^[^ ^233^ ^]^ Unlike other IrX_2_s with pyrite structures, IrTe_2_ possesses a layered 1T structure composed of edge‐sharing IrTe_6_ octahedra.^[^ ^234^ ^]^ Furthermore, owing to various interactions and orderings,^[^ ^235^, ^236^, ^237^ ^]^ bulk 1T‐IrTe_2_ exhibits temperature‐dependent multiple charge‐ordering states and structural transformation while retaining its metallic nature.^[^ ^237^, ^238^ ^]^ Moreover, the interlayer distance in 1T‐IrTe_2_ is notably shorter than that in other typical vdW materials, rendering it an ideal model system for analyzing the interlayer coupling effect as a function of the number of layers.^[^ ^237^, ^239^, ^240^ ^]^ Hwan et al. revealed a unique insulating dimer ground state (2 × 1) in a single layer, which contradicted the metallic behavior observed in bilayer‐to‐bulk 1T‐IrTe_2_.^[^ ^241^ ^]^ Authors have also reported that the interlayer coupling between Te atoms can modify the phonon and charge susceptibilities in IrTe_2_, inducing a metal‐to‐insulator transition from a bilayer to single layer.

Group‐10 (Ni, Pt, and Pd) TMX_2_s with *d* ^6^ configuration exhibit the most stable 1T structure in the case of both bulk and single layer, while 3R is metastable.^[^ ^242^ ^]^ Owing to strong interlayer hopping along the *c*‐axis for *p*_*z*_ states as opposed to that observed in *p*_*x,y*_ states, group‐10 TMX_2_s commonly exhibit strong *k*_*z*_‐dispersion and large bandwidth for *p*_*z*_‐bands.^[^ ^243^, ^244^ ^]^ Similar to VS_2_ or VSe_2_, a few group‐10 TMDs, such as Pt and Pd, exhibit a thickness‐dependent metal–insulator transition because of the strong interlayer interactions that occur when the thickness is reduced to a single layer.^[^ ^114^, ^120^, ^242^, ^245^ ^]^ Additionally, PdS_2_^[^ ^108^, ^246^ ^]^ and PdSe_2_^[^ ^114^, ^115^, ^247^ ^]^ can uniquely host orthorhombic pentagons as the most stable structure, resulting in several anisotropic characteristics. These exotic structures are characterized by a strong interlayer interaction with a low energy barrier, which promotes interlayer diffusion of Se vacancies and leads to the formation of puckered pentagonal structures.^[^ ^248^ ^]^ Moreover, unlike black phosphorus, which is unstable in ambient conditions, these materials are relatively stable in open air despite their strong anisotropy, increasing their prospects for practical applications.^[^ ^114^ ^]^ Unlike NiS_2_ with a pyrite structure, NiSe_2_ and NiTe_2_ exhibit a 1T‐vdW‐layered structure with a metallic band structure.^[^ ^249^, ^250^, ^251^, ^252^ ^]^ Zhao et al. synthesized highly uniform 1T‐NiTe_2_ nanosheets with excellent electric conductivities, which linearly increase with the increase in thickness.^[^ ^251^ ^]^

Finally, owing to the similarities in structural features with TMDs, phase engineering research has been predominantly extended to metal chalcogenide nanosheets (MX or MX_2_, where M = Sn or Ge); therefore, we briefly review them here. The phase tuning between MX and MX_2_ can be easily achieved using methods that have been demonstrated in studies on the phase engineering of TMDs. Moreover, unlike TMDs, both Sn‐ and Ge‐based chalcogenides are semiconducting although their bandgap size can be varied by the chemical composition ratio.^[^ ^253^ ^]^ For instance, tin sulfides with chemical formulas of SnS_2_, SnS, and Sn_2_S_3_ exhibit several subpolytype structures. The single‐layer SnS_2_ can crystallize into either a trigonal 1T or hexagonal 1H phase,^[^ ^254^ ^]^ whereas the multilayer SnS_2_ can exhibit polytypism in 1T,^[^ ^255^, ^256^ ^]^ 2H,^[^ ^257^, ^258^, ^259^ ^]^ and 4H,^[^ ^257^, ^258^, ^259^ ^]^ structures. Saho et al.^[^ ^254^ ^]^ reported that potassium halides can effectively promote the in‐plane growth of 1T‐SnS_2_ nanosheets by reducing the surface migration barriers and increasing the adhesive force between the adsorbed SnS_2_ molecules and substrate. On the contrary, 2H‐halide was determined to be more suitable for the growth of 2H‐SnS_2_ nanosheets. Unlike SnS_2_, SnS can possess an orthorhombic (*Pnma*) or a cubic phase (*Cmcm*).^[^ ^260^ ^]^

### 4.2 Group‐VI TMX_2_s for Phase Engineering

Despite numerous studies on various TMX_2_s from different groups, research on phase engineering, including that on group‐VI TMX_2_s, has not reached a mature level yet. This is particularly observed with respect to 1) the correlation between structural and electronic transformations and 2) the relatively wider engineering margin, which are notably pronounced in group‐VI TMX_2_s when compared with that of TMX_2_s from other groups.

**Figure 7:**
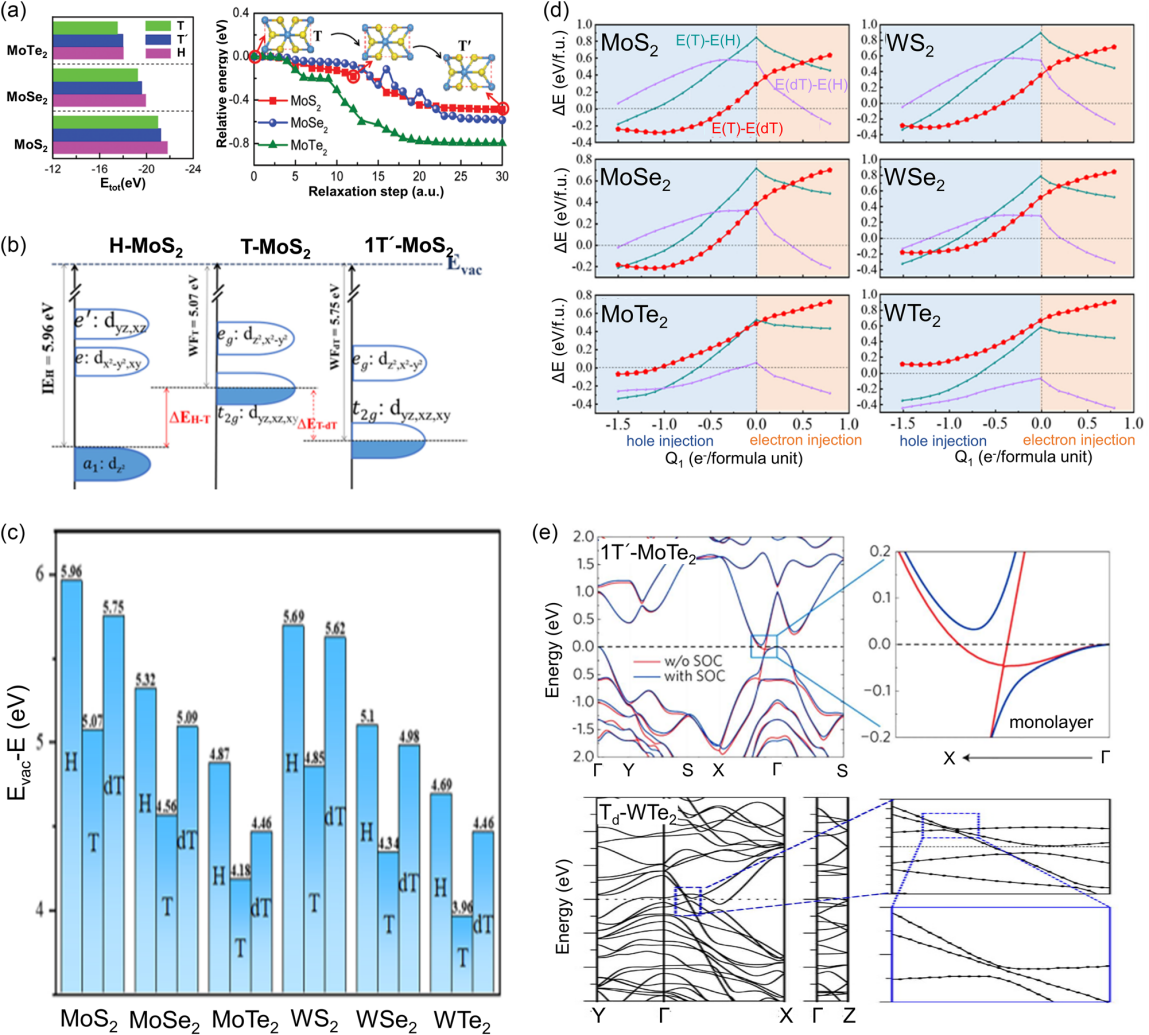
a) Energy comparison of single‐layer TMX_2_ in the three phases, namely, H, T, and 1T′ (left). Energy profiles of TMX_2_ monolayers as a function of relaxation steps (right).^[^ ^263^ ^]^ Reproduced with permission.^[^ ^263^ ^]^ Copyright 2020, Royal Society of Chemistry. b) Ionization energy of the H phase and work function of 1T and 1T′ phases of MoS_2_ and^[^ ^87^ ^]^ c) all group‐VI TMDs.^[^ ^87^ ^]^ d) Energy differences as a function of electron and hole injections among the three phases.^[^ ^87^ ^]^ b–d) Reproduced with permission.^[^ ^87^ ^]^ Copyright 2022, American Physical Society. e) Band structures of MoTe_2_ (top) and WTe_2_ (bottom).^[^ ^264^, ^271^ ^]^ Reproduced with permission.^[^ ^264^ ^]^ Copyright 2015, Springer Nature Limited. Reproduced under the terms of the CC BY 4.0 license.^[^ ^271^ ^]^ Copyright 2015, The Authors, published by Springer Nature.

Among group‐VI TMX_2_s, Cr‐based TMDs, such as CrS or Cr_2_S_3_, are sufficiently metastable to spontaneously transform to the lower oxidation state. On the contrary, Mo‐ and W‐based TMX_2_s with *d* ^2^ configuration primarily exhibit a semiconducting 2H/1H structure as their most stable phase;^[^ ^261^ ^]^^]^ except for WTe_2_, which exhibits the 1T′ phase.^[^ ^262^ ^]^ Additionally, as the counterparts of the 1T phase exhibit metallic characteristics, 1T can be considered the metallic phase in group‐VI TMDs. However, the 1T phase is often unstable, thereby requiring self‐stabilization through additional distortion via the Peierls transition; this is also referred to as a type of CDW. Several studies have reported that the relative stabilities among 2H (1H), 1T, and 1T′ phases exhibit a monotonic trade‐off relationship with the period number of a chalcogenide atom, which is X for both Mo and W.^[^ ^87^, ^99^ ^]^ In other words, the energy difference between 1H and 1T (or 1T′) decreases with the increasing period of X, whereas that between 1T and 1T′ exhibits a monotonic increase (**Figure** **7**a). Zhou et al. demonstrated that the phase stability of Mo‐based TMX_2_s is governed by Mo‐4*d* orbitals and *p‐d* hybridization between the Mo and X atoms.^[^ ^263^ ^]^ As the Mo‐*t*_2g_ orbital is occupied by injected electrons, the degree of *p‐d* hybridization progressively increases, decreasing the energy barriers required for phase transition. Consequently, the energy barrier for a phase transition is the highest for MoS_2_ and the lowest for MoTe_2_. A similar result was reported by Li et al.,^[^ ^87^ ^]^ where electron injection caused a phase transition from 1H to 1T (Figure 7b). Moreover, Li et al. provided a more unified interpretation of charge‐induced phase transformation in group‐VI TMX_2_s (Figure 7c). The authors proposed that the work function of TMX_2_ increases with the increase in the atomic number of X for both Mo and W. Therefore, hole‐injection (or electron‐removal) can trigger a phase transition from 2H/1H to 1T for MoS_2_, WS_2_, MoSe_2_, and WSe_2_ (Figure 7d). Conversely, electron injection leads to a phase transition from 1H to 1T′ owing to the smaller work function of 1T in comparison with that of 1T′. Therefore, the energy difference between 1H and 1T′ lies within the range of a few hundred millivolts, where both phases are accessible and reliably switchable.^[^ ^264^, ^265^ ^]^ Most experimental investigations of TMDs with sulfides and selenides have revealed stable 2H/1H states with only a few exceptions.^[^ ^266^, ^267^ ^]^ Researchers have also demonstrated that WTe_2_ consistently displays the 1T phase under ambient conditions, whereas both 2H/1H and 1T′ phases coexist in MoTe_2_, with the ratio dependent on the specific environmental conditions and preparation methods. The formation of 1T′‐MoTe_2_ is known to be more favorable at higher quench^[^ ^265^ ^]^ or annealing temperatures in a Te‐rich atmosphere.^[^ ^268^ ^]^ At present, growing sizable 2H‐WTe_2_ with stability in ambient conditions is considered challenging because of the small energy difference.^[^ ^76^ ^]^ This interpretation has broad applicability to other experimental observations.^[^ ^264^, ^269^ ^]^

Typically, MoTe_2_ and WTe_2_ are regarded as distinctive subsets among group‐VI TMX_2_s owing to their diverse polymorphism and uniquely intriguing properties that can be readily manipulated by a marginal energy difference. For instance, the 1T′ phase of MoTe_2_ or WTe_2_ can be stacked in two different structures: 1) monoclinic 1T′(β‐phase) and 2) orthorhombic *T*_d_ (γ‐phase). Despite similar AA‐stacking, the β‐phase exhibits a tilting of the axis (≈4°) along the vertical direction with respect to that in the γ‐phase.^[^ ^270^, ^271^ ^]^ According to Santosh et al.,^[^ ^272^ ^]^ the unusual transition behavior can be explained by the competition between the ligand field stabilization energy and CDW energy. The modest energy difference in MoTe_2_ enables a relatively smooth phase transition to the *T*_d_ phase. Furthermore, the greater elongation of the distance between TM and X in WTe_2_ causes a more significant decrease in the ligand field stabilization energy and increases the CDW energy, establishing the *T*_d_ phase as a more favorable state.^[^ ^272^ ^]^ Therefore, both *T*_d_‐MoTe_2_ and *T*_d_‐WTe_2_ commonly exhibit semi‐metallic structures,^[^ ^264^, ^271^ ^]^ as indicated in Figure 7e. Additionally, a narrow indirect bandgap opening (≈50 mV) occurs in 1T_d_‐WTe_2_ with the decrease in the number of layers, resulting in differences between the thickness‐dependent electrical and thermal voltages.^[^ ^273^ ^]^ The unique electronic band structures of T_d_ are derived from the significant *p‐d* hybridization, leading to the splitting of *t*_2g_ along the Γ‐Y direction and resulting in the minimum density of state at the Fermi level. Additionally, the electron‐ and hole pockets intersect the Fermi energy, further enhancing the semi‐metallic properties.^[^ ^273^, ^274^ ^]^ Moreover, the adjustment of growth factors, such as pressure,^[^ ^94^ ^]^ temperature,^[^ ^96^ ^]^ and Te vacancy,^[^ ^275^ ^]^ results in mutually convertible 1T′ and 1T_d_ phases. Rhodes et al.^[^ ^276^ ^]^ demonstrated that the T_d_ phase of MoTe_2_ can be stabilized even at room temperatures through tungsten doping. Furthermore, Kim et al.^[^ ^277^ ^]^ investigated the reasons for the stability of the 1T_d_ phase in WTe_2_ even under ambient conditions, while the same phase was absent in MoTe_2_ above ≈250 K.^[^ ^278^ ^]^ The authors attributed the difference in phase stability to the valence electron configuration dependent on interlayer bonding as VBE is composed of two different *p‐d* hybridizations. In β‐MoTe_2_, higher electron occupancy tends to lower the energy by changing the phase from *β* to *γ* at room temperature. However, owing to the limited contribution of Te *p‐*orbitals to VBE in WTe_2_, the corresponding transition is rarely observed. Moreover, the atomic layers of T_d_‐MoTe_2_ and T_d_‐WTe_2_ are featured as type‐II Weyl semimetals,^[^ ^279^, ^280^, ^281^ ^]^ rendering them suitable for the spin Hall effect.^[^ ^282^ ^]^ As various phases have been discovered in single layers of 1T′‐group‐VI TMX_2_s, such as WTe_2_ and MoTe_2_ that include topologically protected edge states^[^ ^283^, ^284^ ^]^ and quantum spin Hall gaps,^[^ ^285^ ^]^ their potential application in spintronic devices has been extensively investigated.

## 5 Strategies for Phase Engineering

As discussed in previous sections, vdW TMDs are highly susceptible to both surface kinetics and thermodynamics,^[^ ^98^ ^]^ enabling the TM and X atoms in various TMX_2_s to spontaneously and diversely coordinate without introducing defects or impurities. Therefore, appropriate energy should be provided to overcome the energy barrier between the competing phases either using growth parameters or post‐growth treatments. To date, various methods have been explored to selectively regulate phase behavior; they can be broadly classified into two categories: 1) direct growth and 2) post‐growth methods. We first discuss CVD in the direct growth category, considering the frequency and adaptability to applications. Second, we review the development of various post‐growth treatment methods, including intercalation methods, for phase engineering.

### 5.1 CVD Growth

Direct growth methods, such as CVD, chemical vapor transport (CVT), and molecular beam epitaxy (MBE) methods, exhibit certain advantages in obtaining high‐quality vdW materials at a sizable scale. Moreover, these methods can trigger polymorphic phase transitions by finely tuning the reaction kinetics using the growth parameters. The recent progress in CVD has opened up new avenues for realizing phase engineering in low‐dimensional materials, such as TMD nanosheets, while maintaining a high degree of crystallinity. Therefore, the related advancement and its prospective are reviewed in this section.

#### 5.1.1 Key Factors for Growth of the Phase‐Regulated MoTe_*2*_

For CVD‐grown vdW TMDs, the final state is dependent on the activation energy barriers for the phase transition, which can be controlled using growth parameters, such as promoter, temperature, atmosphere, or other growth media. Particularly, because MoTe_2_ exhibits moderate energy of ≈40 and 50 mV for 1T′ and 1T, respectively, with respect to 2H,^[^ ^76^ ^]^ MoTe_2_ has been identified as a primary platform to feasibly perform phase modulation through direct synthesis. Although WTe_2_ is a possible secondary candidate, the sizable growth of a stable 2H phase is often affected by the extremely small energy difference (≈–10 mV),^[^ ^286^ ^]^ which enables the synthesis of 1T′ and T_d_ phases.

**Figure 8:**
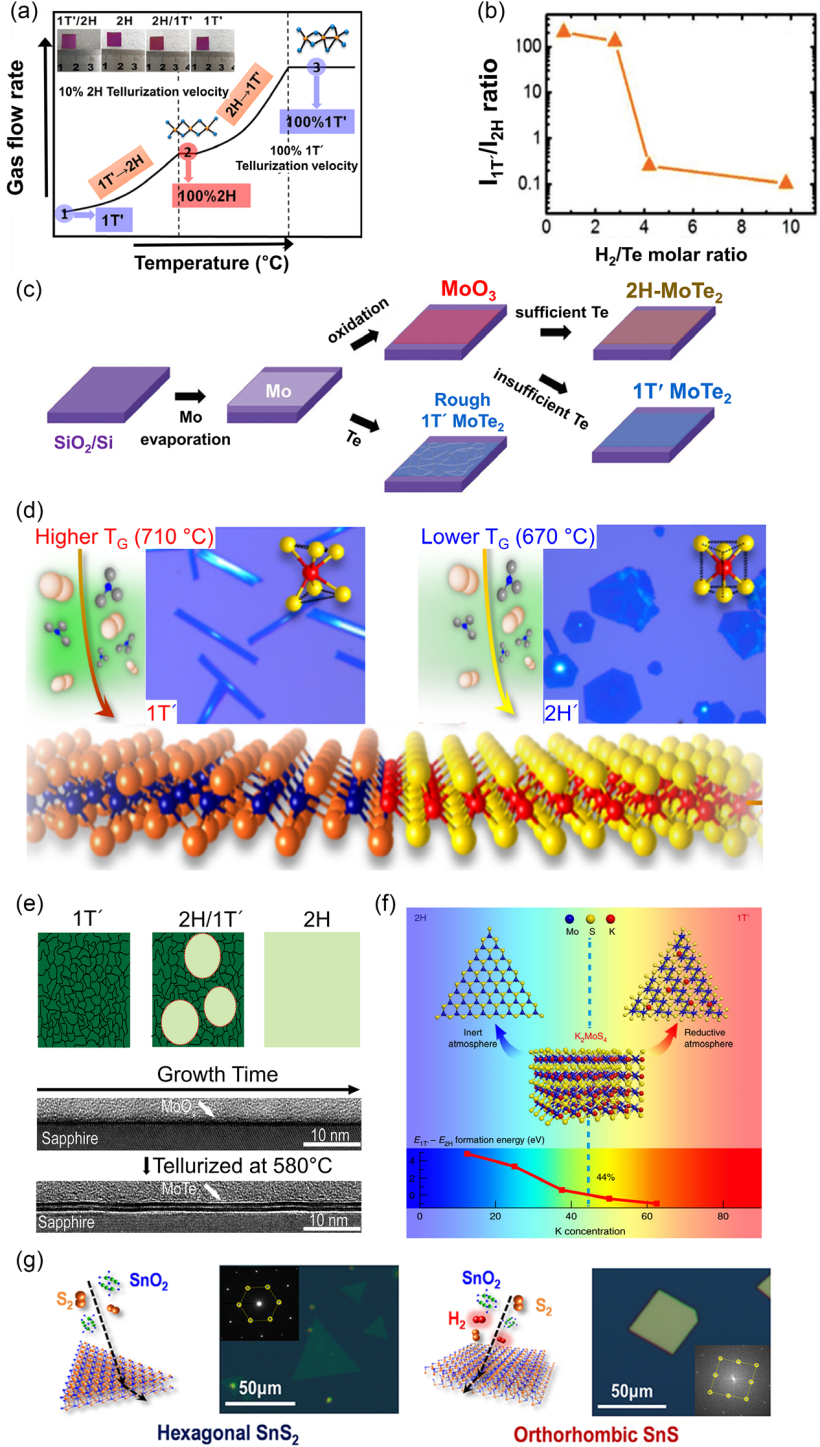
a) Phase evolution of CVD‐grown MoTe_2_ modulated by carrier gas flow rate and tellurization temperature.^[^ ^287^ ^]^ Reproduced with permission.^[^ ^287^ ^]^ Copyright 2017, American Chemical Society. b) Raman spectra of the phase evolution of MoTe_2_ films with the increase in the H_2_‐to‐Te molar ratio.^[^ ^290^ ^]^ Reproduced with permission.^[^ ^290^ ^]^ Copyright 2018, Wiley‐VCH. c) Schematic of the growth process for 1T′‐ and 2H‐MoTe_2_ using Mo and MoO_3_ as precursors.^[^ ^293^ ^]^ Reproduced with permission.^[^ ^293^ ^]^ Copyright 2016, Wiley‐VCH. d) Polymorphic integration of few‐layer 1T′/2H‐MoTe_2_ within the same atomic plane modified by temperature.^[^ ^297^ ^]^ Reproduced with permission.^[^ ^297^ ^]^ Copyright 2017, Springer Nature Limited. e) Nucleation and growth of the 2H phase from 1T‐MoTe_2_.^[^ ^301^ ^]^ Reproduced with permission.^[^ ^301^ ^]^ Copyright 2021, American Chemical Society. f) The phase‐controlled synthesis of K_*x*_MoS_2_ as a function of the concentration of K.^[^ ^102^ ^]^ Reproduced with permission.^[^ ^102^ ^]^ Copyright 2018, Springer Nature. g) Growth schematics and representative optical microscope images of hexagonal SnS_2_ in N_2_ (left) and orthorhombic SnS in N_2_−H_2_ (right).^[^ ^255^ ^]^ Reproduced with permission.^[^ ^255^ ^]^ Copyright 2015, American Chemical Society.

In principle, the highly enriched tellurization can be beneficial for stabilizing the 2H phase in MoTe_2_ while preventing Te vacancies. However, insufficient telluride can facilitate the formation of the 1T′ phase by reducing the free energy of 1T′ via Te deficiency. Therefore, the feeding rate of Te atoms is considered the first key factor in phase engineering. Several studies^[^ ^287^, ^288^ ^]^ directly revealed that a simple change in Te flux or velocity leads to the phase transition in few‐layer MoTe_2_; the formation of the 2 phase is more probable with high Te flux (or velocity), whereas the 1T′ phase is preferred under lower Te conditions (**Figure** **8**a). Xu et al.^[^ ^289^ ^]^ investigated the evolution of the nucleated 2H phase to the adjacent 1T phase. According to the literature, prolonged growth times favor the formation of a larger 2H phase unless the decomposition is initiated by Te vaporization at high temperatures. Furthermore, as H_2_ gas is generally used as a reducing agent in the CVD growth of 2D TMDs, Kim et al.^[^ ^290^ ^]^ investigated the phase engineering of few‐layer MoTe_2_ films by controlling the H_2_ molar flow rate; they determined that the molar flow rate can play a vital role in compensating for the effect of Te flux. In other words, a low H_2_ flux results in mixed phases of 1T′ and 1T′–2H, while the 2H phase is achievable at a high H_2_ flux (Figure 8b). Similarly, the phase of WTe_2_ can be adjusted by harnessing the reaction rate of Te with W film using H_2_Te as an intermediate in the presence of H_2_ gas.^[^ ^291^ ^]^

Second, the type of metal precursor, including its volatility or intermediate state, can play a critical role in controlling the phase of TMDs during the CVD process. Zhou et al.^[^ ^292^, ^293^ ^]^ revealed that MoO_3_ reacts more easily with Te and forms 2H‐MoTe_2_, whereas Mo or MoO_3−*x*_ (0 < *x* < 3) is more likely to produce 1T′ (Figure 8c). This phenomenon can be explained based on the drastic volume change observed when Mo or MoO_2_ transitions to MoTe_2_, which favors the 1T′ phase to compensate for the strain energy. By contrast, the relatively mild change in volume from MoO_3_ to MoTe_2_ is considered tolerable even with 2H‐MoTe_2_. Fraser et al.^[^ ^294^ ^]^ employed the mixture of Fe and Te for their analysis rather than the typical Te powder, resulting in the availability of Te vapor only above approximately 600 °C. Therefore, while the Mo‐seed layer leads to the formation of semiconducting 2H‐MoTe_2_, MoO_3_ leads to the formation of 1T′‐MoTe_2_ during a single CVD reaction process. Additionally, Mo(W)‐Cl bonding of MoCl_5_ (WCl_5_) exhibits substantially lower binding energy than Mo–O(W–O); this feature can be useful for decreasing the growth temperature. Based on the aforementioned difference, Zhou et al.^[^ ^291^ ^]^ reported that 1T′‐MTe_2_ and 1T′‐WTe_2_ can be synthesized on a large scale. Moreover, Yang et al.^[^ ^295^ ^]^ reported the growth of the 3R phase, wherein the independent heating of MoCl_5_ at a lower temperature plays a vital role in fine‐tuning the growth mechanism of MoTe_2_. This enables the formation of 3R‐MoTe_2_ at low temperatures, suppressing the phase transition to 1T′. The protocol for precursor supply is also essential as the type of precursor. In a typical one‐step CVD process, both the chalcogen and metal precursors are positioned along with the target substrate under a single heating zone. The deposition of gas‐phase TMD molecules onto the substrate benefits the formation of stoichiometric vdW TMD. In the case of MoTe_2_, the growth is typically performed as a one‐step process based on the reaction of gas or liquid between the Te vapor and Mo sources, such as MoO_*x*_ or Mo intermediate products.^[^ ^292^, ^295^ ^]^ In comparison with the one‐step CVD, the Mo precursor in the two‐step CVD is deposited in advance as a seed on the substrate. Subsequently, the seed is transformed into 1T′‐ and 2H‐MoTe_2_ under the Te atmosphere.^[^ ^294^ ^]^ This method can avoid contamination or the alloying effect by depositing the vaporized chalcogen atoms on the transition metal film. It also presents more versatile and flexible options for fabricating vdW TMDs in terms of composition and thickness.

Third, the temperature can be a key factor in directly controlling the phase‐selective growth of vdW TMDs in thermodynamic methods, such as CVD. The temperature‐driven phase transitions are particularly effective for telluride TMDs, as they typically cause the loss of Te atoms because of the weak chemical bonding between Mo and Te^[^ ^264^ ^]^ (Figure 8d). Several studies have analyzed the effect of reproducible growth temperature on the phase in MoTe_2_.^[^ ^287^, ^296^ ^]^ Moreover, the cooling process exhibits a considerable effect on the final ground state of MoTe_2_. This phenomenon has been consistently observed in the one‐step CVD process,^[^ ^297^ ^]^ wherein high growth temperatures (>710 °C) facilitate the growth of 1T′‐MoTe_2_ and low growth temperatures (<670 °C) are preferred for 2H‐MoTe_2_. According to Empante et al.,^[^ ^265^ ^]^ 2H, 1T, and 1T′ phases of MoTe_2_ can be selectively produced by controlling the growth and quenching temperatures during the one‐step CVD process; here, the slow cooling to 450 and 350 °C followed by quenching led to 1T and 1T′ phases, respectively, whereas cooling to 100 °C produced the 2H phase. The results revealed that the 1T′ phase is more stable at high temperatures (approximately above 900 °C), whereas the phase is only metastable at room temperature. Additionally, the 1T phase can be stabilized if the cooling process can be performed precisely. Hoang et al. reported that the final state of MoTe_2_ depends on the dwell time.^[^ ^298^ ^]^ However, serious caution is necessary with respect to temperature compared with other factors, such as the precursor, strain, or deficiency, because the temperature can affect the other factors in a complex manner. For instance, decreasing the growth temperature may not always result in the synthesis of 2H‐MoTe_2_ because an inadequate flux of Mo and Te can occur at temperatures lower than their critical melting points, eventually leading to the growth of the 1T′ phase. Owing to these complex thermal issues, catalysts such as NaCl and KCl can be utilized to reduce the melting points of Mo precursors and increase the feeding rate of Mo.^[^ ^299^ ^]^ Cui et al. used a mixture of NaCl and MoO_3_ to synthesize MoTe_2_, wherein the 1T_d_ phase could be maintained even at room temperature.^[^ ^300^ ^]^

Finally, the substrate effect can be considered a key factor impacting the phase‐controlled growth. For the vdW epitaxial growth, the seed film is expected to undergo substrate‐insensitive tellurization and transition from 1T′ to 2H phase. However, as reported by Hynek et al.,^[^ ^301^ ^]^ the lateral expansion of MoTe_2_ films is more prominent on sapphire rather than on SiO_2_ owing to the distinct adsorption energy difference between the substrates (Figure 8e). Similar observations have been reported for the diffusivity of MoO_3_ on amorphous Al_2_O_3_ versus SiO_2_ substrates.^[^ ^302^ ^]^ The results indicate that the type of substrate can significantly impact both the quality and final phase of MoTe_2_ films.

#### 5.1.2 CVD‐Based Phase Engineering in Other TMDs

Using the strategies demonstrated for MoTe_2_, the phase of other vdW TMDs can be similarly regulated. The 1T′ phase was obtained from MoSe_2_^[^ ^303^ ^]^ and WSe_2_,^[^ ^304^ ^]^ whereas the 3R phase was obtained from MoS_2_^[^ ^305^ ^]^ and WS_2_.^[^ ^306^ ^]^ However, in comparison to MoTe_2_, converting the phase in most vdW TMDs typically requires high energy, which renders the process challenging and may necessitate additional assistance. Liu et al.^[^ ^102^ ^]^ reported that the phase of MoS_2_ can be more readily converted by varying the concentration of K in the K_*x*_MoS_4_ precursor under a reductive atmosphere. When the concentration of K is below a critical concentration, 2H‐MoS_2_ is formed in the Ar atmosphere owing to the significantly higher formation energy of the 1T′ phase than that of the 2H phase (Figure 8f). When the concentration of K exceeds a critical value, the 1T′ phase is favored in a mixture of H_2_ and Ar atmosphere as it is more stable compared to the 2H phase in K_*x*_MoS_2_. Moreover, this method can be used for synthesizing other 1T′‐phase TMDs, such as 1T′‐WS_2_, by using K_2_WS_4_ as the growth precursor. TMDs in other groups (NbS_2_ (2H,^[^ ^307^, ^308^ ^]^ 1T,^[^ ^309^ ^]^ and 3R^[^ ^310^ ^]^) and TaSe_2_ (2H,^[^ ^311^ ^]^ 1T,^[^ ^312^ ^]^ and 3R^[^ ^312^ ^]^) also exhibit phase‐selective growth. However, their phase tuning diversity is relatively narrow compared with that of *M*‐ or W‐based TMDs, and the corresponding changes in the electronic band structures are less distinct.

Owing to the similarities in structural features of TMDs, the proposed phase‐selective CVD synthesis methods can be applied to controlling the phase of metal chalcogenide nanosheets (MX or MX_2_). For instance, the phase tuning between MX and MX_2_ can be easily achieved by adjusting the growth temperature. Furthermore, unlike TMDs, both Sn‐ and Ge‐based chalcogenides are semiconducting although their bandgap sizes can vary depending on the chemical composition ratio.^[^ ^253^ ^]^ During the CVD process, SnS_2_ flakes are typically synthesized by evaporating the SnO gas molecules and reacting them with sulfur atoms on a substrate, resulting in a diverse range of structures. Remarkably, Ahn et al.^[^ ^255^ ^]^ successfully synthesized both hexagonal 2H‐SnS_2_ and orthorhombic SnS and demonstrated that the final crystal structure can be controlled based on the thermodynamic conditions by inserting H_2_ gas during growth. Furthermore, thermodynamic calculation revealed that by adding H_2_ gas at a constant temperature, Gibbs free energy for SnS can become more negative compared with that of SnS_2_ (Figure 8g). Notably, SnS has been reported to be a *p*‐type semiconductor owing to the generated Sn vacancies, whereas SnS_2_ has been considered to be n‐doped.^[^ ^255^, ^257^ ^]^ Additionally, Price et al.^[^ ^313^ ^]^ reported that CVD growth at low temperatures ranging from 300 to 500 °C promotes the formation of SnS_2_, whereas higher temperatures (approximately 545 °C) favor the formation of SnS. Huang et al.^[^ ^314^ ^]^ also confirmed a similar thermal effect in tin selenide(s) by verifying that the final crystal structure changes from square‐shaped SnSe to triangular SnSe_2_ along with the decreasing temperature gradient.

#### 5.1.3 Hybridization with Different Atoms

**Figure 9:**
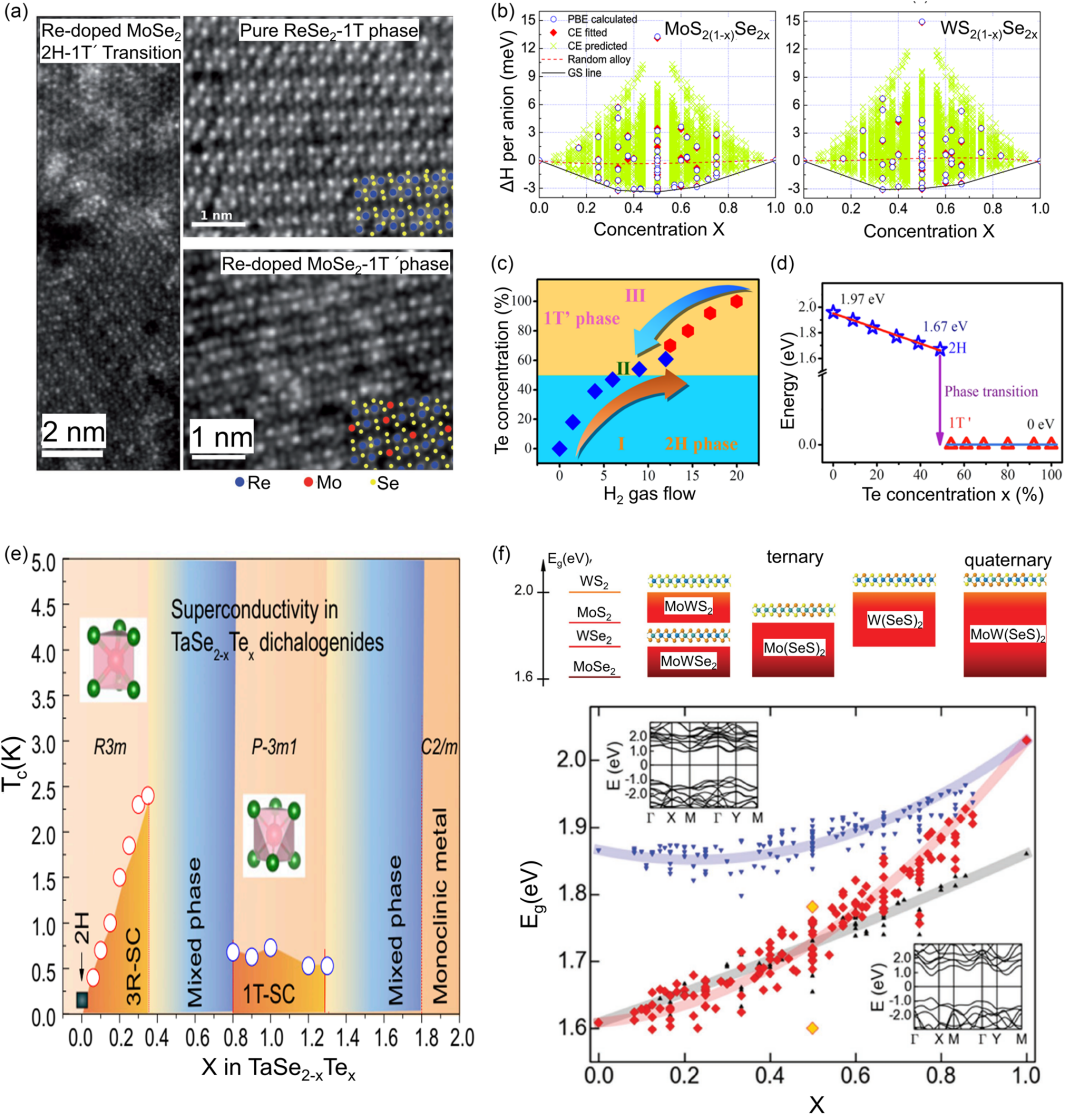
a) HAADF‐TEM image of Re‐doped single‐layer MoSe_2_ and ReSe_2_.^[^ ^324^ ^]^ Reproduced with permission.^[^ ^324^ ^]^ Copyright 2017, Wiley‐VCH. b) First‐principle calculated formation enthalpies for MoS_2(1−*x*)_Se_2*x*_ and WS_2(1−*x*)_Se_2*x*_.^[^ ^327^ ^]^ Reproduced with permission.^[^ ^327^ ^]^ Copyright 2013, AIP Publishing. c) The phase evolution driven by Te concentration and H_2_ flow.^[^ ^332^ ^]^ d) Optical bandgap values of 2H and 1T′ phases in WS_2(1−*x*)_Te_2*x*_ alloys.^[^ ^332^ ^]^ c,d) Reproduced with permission.^[^ ^332^ ^]^ Copyright 2020, Elsevier. e) The phase diagrams of TaSe_2−*x*_Te_*x*_ dependent on the concentration of Te and temperature.^[^ ^342^ ^]^ Reproduced under the terms of the CC BY license.^[^ ^342^ ^]^ Copyright 2015, The Authors, published by PNAS. f) Bandgaps of TMX_2_ (TM = Mo and W; X = S and Se) and their alloys (top). Calculated bandgaps of Mo_1−*x*_W_*x*_Se_2(1−*x*)_S_2*x*_ (large red diamonds), Mo_1−*x*_W_*x*_S_2_ (small blue downward arrows), and MoSe_2(1−*x*)_S_2*x*_ alloys (black upward triangles; bottom).^[^ ^343^ ^]^ Reproduced with permission.^[^ ^343^ ^]^ Copyright 2017, Wiley‐VCH.

As demonstrated in MoTe_2_, vdW TMDs can slightly distort their intrinsic phases by introducing foreign atoms or vacancies, which can be attributed to the diverse *d*‐orbital electron configuration. Foreign atoms can be incorporated into the parent crystal structure by utilizing the flexibility of TMDs and replacing the intrinsic atoms or filling the vacancies. In other words, CVD can serve as a facile approach to synthesizing dopant‐induced polynary TMD in a controllable manner. For instance, the primary carrier type in MoS_2_ is an electron with doping of Fe,^[^ ^315^ ^]^ Co,^[^ ^316^ ^]^ or Au^[^ ^317^ ^]^ atoms; however, it can be changed to a hole by doping with Ta^[^ ^318^ ^]^ or Nb.^[^ ^319^ ^]^ In general, doping TMX_2_ with either Na or Rb, which belong to the alkali metal group, can transform it into an n‐type semiconductor with substantial lattice expansion mainly along the c‐direction, which can also induce phase transformations. Kang et al.^[^ ^320^ ^]^ revealed that the bandgap of 2H‐TMX_2_ (MoS_2_, MoSe_2_, WS_2_, and WSe_2_) can be modulated in the range from ≈0.8 to ≈2.0 eV through in situ surface doping of Rb at the concentration of ≈10^12^ cm^−2^. This band tunability can be attributed to the structural change caused by the formation of an electric dipole within the surface bilayer, which breaks inversion symmetry and leads to spin splitting of valence bands. Recently, William et al.^[^ ^321^ ^]^ theoretically explored the influence of six different dopants (Mo, Ni, Sc, Ti, V, and W) at metal site of TMX_2_ in terms of the structural and electrical properties. The authors found that the Ni and Sc can most significantly distort the X–X bonding length or X–*M*–X bonding angle in 1L‐MoTe_2_ and WTe_2_, respectively, which leads to a significant reduction in semiconducting bandgap or lower the stability. Additionally, such distortions can activate the optical conductivities at much lower energy than those of the pure parent system. On the contrary, Mo or W dopants have only a minor impact on the density of state due to their similar atomic radii (0.79 and 0.80 Å, respectively) and oxidation states (+6), except for *p*‐type doping. The results reflect that substitutional doping of transition metal atoms from group IV to group XII brings better effects due to their similar atomic radii and oxidation states. Onofrio et al.^[^ ^322^ ^]^ suggested a general doping strategy for single‐layer TMX_2_ using DFT calculations. The authors suggested that *E*_F_ would be lowered to VBE via the substitutional dopants in early TMs (i.e., Sc, Ti, Zr, V, and Nb), resulting in p‐type behavior. As for mid TMs (Tc, Ru, and Rh), as the *d*‐orbital is occupied, *E*_F_ increases toward CBE, leading to a transition from p‐type to n‐type behavior. When doped with late TMs, a large number of gap states are anticipated with poor transport properties. Furthermore, Ho et al.^[^ ^323^ ^]^ reported that the phase of vdW TMDs can be altered by doping different transition metal atoms. In the literature, 1T phase was observed in intrinsic 2H‐WSe_2_ by doping Cr during the CVD process, and the portion increased with the concentration of Cr owing to electron doping caused by Cr_W_−V_Se_ complexes. Moreover, Kochat et al. reported that Re‐doping in TMDs can be used to tailor the mixing ratio of 1T and 1H phases during the CVD process because Re atoms operate as nuclei to trigger the transformation to 1T^[^ ^324^ ^]^ (**Figure** **9**a). Similarly, Li et al.^[^ ^325^ ^]^ synthesized single‐layer TMX_2_ doped with either Re or V through the CVD process. The authors observed that a semiconductor‐to‐metal transition can be adjusted by varying the doping density. Interestingly, while V dopants were uniformly dispersed within the MoS_2_ structure without phase segregation, the distorted 1T‐ReSe_2_ phase appeared among MoS_2_ structure after the Re‐doping density exceeded ≈25% and the phase separation became more pronounced at higher Re doping concentration.

The alloying of vdW TMDs is a unique, but powerful process to tailor the structure, wherein different components can be mutually soluble in a wide range of proportions. The alloying methods can be divided into three types: transition metal replacement, chalcogenide replacement, and metal and chalcogenide replacement. Specifically, similar structures of TMX_2_s are exploited, using TM (or X) atoms from the same group (or the same period) as dopant atoms to form stable ternary alloy phases.^[^ ^326^ ^]^ Kang et al.^[^ ^327^ ^]^ theoretically predicted the stability and band bowing effects of various group‐VI TMX_2(1−*x*)_ X′_2(1−*x*)_ alloys. Lattice constants of TMX_2_ alloys based on MoX_2_ or WX_2_ alloys follow a simple linear rule of mixtures: Therefore, the final lattice constants of the alloy can be calculated as the weighted average of the lattice constants of the individual components. Similarly, the author assumed that the band edge positions and bandgap values of alloys also follow an analogous linear combination, with a slight modification to account for the volume deformation effect on the electronic band structure in low‐dimensional system. Based on the assumption, the bandgap of MoS_2*x*_Se_2(1−*x*)_ alloys can be described as: *E*_g_(*x*) = *xE*_g_(MoS_2_) + (1−*x*)*E*_g_(MoSe_2_)– *bx*(1−*x*), where *b* is the bowing parameter. The value was reported as 0.05 for 1 L‐MoS_2*x*_Se_2(1−*x*)_.^[^ ^327^, ^328^ ^]^ As the result, the authors proposed that (S, Se) alloys can be more stable at a critical concentration of *x*, whereas (Se, Te) and (S, Te) alloys lack ordered configurations (Figure 9b). Additionally, Kang et al.^[^ ^327^ ^]^ reported that the gradient of VBE becomes greater than CBE with increasing *x*. Similarly, Chen et al.^[^ ^329^ ^]^ also exhibited a large bowing effect in CBE, while the VBM showed no bowing. Similarity, Gan et al.^[^ ^330^ ^]^ suggested that the CBE exhibited a bowing effect, while the VBE changed linearly with *x* in Mo_(1−*x*)_W_*x*_S_2_. Dong et al.,^[^ ^331^ ^]^ addressed such distinct behaviors of CBE and VBE to the different orbital configuration in the band. According to the literature, the linear shift of VBE is attributed to *d*_*xy*_ and $d_{x^{2}-y^{2}}$ orbitals of W‐doing element, resulting in an increase with the doping concentration of W. On the contrary, while CBE is mainly contributed by $d_{3z^{2}-r^{2}}$in MoX_2_, it is influenced by $d_{x^{2}-y^{2}}$, $d_{3z^{2}-r^{2}}$ and *d*_*xy*_ orbitals in the case of WX_2_. Therefore, while the W dopant can cause only a slight upshift of CBE below *x* < 0.24, its contribution becomes dominant, further enhancing the upshift above *x* > 0.24. Compared to the case of transition metal, the bandgap of the MS_2(1−*x*)_Se_2*x*_ alloy monotonically shows a red‐shift with increasing replacement concentration of chalcogen atom, Se. On the other hand, telluride‐based alloy shows a much more dramatic dependence on chalcogen replacement compared to others. It has been reported that WS_2(1−*x*)_Te_2*x*_ exhibits bipolar behavior with a structural change from 2H phase to 1T′, when Te is alloyed with chalcogen site. According to Wang et al.,^[^ ^332^ ^]^ for doping concentration below 0.5 at%, WS_2(1−*x*)_Te_2*x*_ behaves as a p‐type semiconductor, while exhibiting n‐type characteristics at higher doping concentrations. This is attributed to the phase transition behavior associated with Te which shows stronger metallicity and higher melting point than other chalcogen atoms.

As introduced in the previous MoTe_2_‐section (Section 5.1.1), modifying the ratio or temperature of the metal or gas precursor is a common strategy used for alloying with different compositions in experiments.^[^ ^333^, ^334^ ^]^ Additionally, H_2_ flow can be used in conjunction with a chalcogenide gas to regulate the portion of the 1T′ phase^[^ ^332^, ^334^ ^]^ or to increase its stability^[^ ^102^ ^]^ (Figure 9c). Mathew et al.^[^ ^335^ ^]^ determined that the final crystal structure of the alloy can vary from the 2H to T_d_ phase, depending on the growth temperature and cooling rate.

Typically, the bandgap in dopant‐induced polynary TMDs is not tunable beyond a certain value (few hundred meV) owing to the constraints imposed by the band structure of the parent TMD. However, the tuning range in the TMD alloy can be significantly expanded from the semiconducting 2H/1H to the metallic 1T′, as observed in MoS_2−*x*_Se_*x*_,^[^ ^336^ ^]^ MoS_2−*x*_Te_*x*_,^[^ ^337^ ^]^ MoSe_2−*x*_Te_*x*_,^[^ ^333^ ^]^ WS_2−*x*_Te_*x*_,^[^ ^332^, ^334^ ^]^ and Mo_*x*_W_1−*x*_Te_2_^[^ ^270^, ^338^, ^339^, ^340^ ^]^ (Figure 9d). As non‐group‐VI TMD alloys, ReS_2*x*_Se_2−*x*_,^[^ ^326^ ^]^ SnS_2*x*_Se_2−2*x*_,^[^ ^341^ ^]^ and TaSe_2−*x*_Te_*x*_^[^ ^342^ ^]^ have also exhibited remarkable tunability depending on their composition.^[^ ^343^ ^]^ Interestingly, Cui et al.^[^ ^326^ ^]^ demonstrated that the conductivity of 1T in ReS_2*x*_Se_2(1−*x*)_ alloys can be tuned from n‐type to bipolar and p‐type by varying the composition while modulating the bandgap size. Lue et al.^[^ ^342^ ^]^ reported that the structure of TaSe_2−*x*_Te_*x*_ can be significantly altered from 2H to 3R, 1T, and monoclinic distorted 1T by tuning the composition ratio. They also demonstrated the significant influence of Te‐doping density on the formation of CDW and superconductivity (Figure 9e). Moreover, Susarla et al.^[^ ^343^ ^]^ synthesized a quaternary TMD alloy (Mo_*x*_W_1−*x*_S_2*y*_Se_2(1−*y*)_) using CVD, wherein the composition of alloys was controlled by adjusting the growth temperature. Based on theoretical calculations and empirical observations, their analysis determined that the gaps of quaternary alloys can span an energy range wider than that of existing ternary TMDs (Figure 9f). Sequentially, the author also^[^ ^344^ ^]^ revealed that increase in the number of components can introduce a miscibility gap, through which an alloy can decompose into a thermodynamically stable heterostructure when the phase is unstable. Indeed, by exploiting the miscibility gap in Mo_(1−*x*)_W_*x*_S_2(1−*y*)_Se_2*y*_, the authors segregated the quaternary alloy into a heterostructure of MoS_2(1−*x*)_Se_2*x*_/WS_2(1−*y*)_Se_2*y*_ by annealing. The result provides a direct evidence about a composition‐driven heterostructure formation in 2D atomic layer systems. In addition, Zi et al.^[^ ^345^ ^]^ theoretically demonstrated that a reversible direct‐to‐indirect band transition is possible by alloying W into MoS_2_/MoSe_2_ and forming Mo_1−*x*_W_*x*_S_2_/MoSe_2_ heterostructure. The author attributed such direct‐to‐indirect transition to the rise of competing valence state at the Γ point, caused by the modified orbital hybridization between MoS_2_, WS_2_, and MoSe_2_ layers with increasing W ratio. Consequently, the author anticipated that reversible direct‐to‐indirect bandgap transition can occur when the concentration of W is higher than ≈0.57. Various reported alloying attempts have been summarized in **Table** **3**.

### 5.2 Ion Intercalation

Foreign species have been successfully intercalated into vdW‐layered materials by inserting them into the interlayer space of host vdW materials. As demonstrated in graphite intercalation,^[^ ^346^ ^]^ the liquid exfoliation with intercalants can be a practical and effective method for obtaining a substantial quantity of exfoliated vdW materials, such as TMDs. The use of intercalants can be classified into three categories, namely, alkali metal atoms, transition metals, and organic molecules. Owing to their different atomic and molecular size and electronegativity compared with the host TMDs, the appropriate intercalation method can vary between wet chemical and electrochemical methods. Specifically, the latter process can be applied to the field of commercial Li‐ion batteries as it is relatively reversible.

In general, intercalation can affect phase stability in two aspects: 1) the release of stored free energy from the charge injection^[^ ^65^, ^152^ ^]^ and 2) the introduction of built‐in strain caused by intercalation.^[^ ^76^, ^347^ ^]^ As intercalation can alter the band‐filling state and Fermi level, it can facilitate the formation of novel charge orderings, such as band splitting, superconductivity, and CDWs. Despite its powerful properties, the stability and yield of intercalation depend on the degree of intercalation and the environment. Therefore, further analysis is required for broader applications.

#### 5.2.1 Alkali Metal

**Figure 10:**
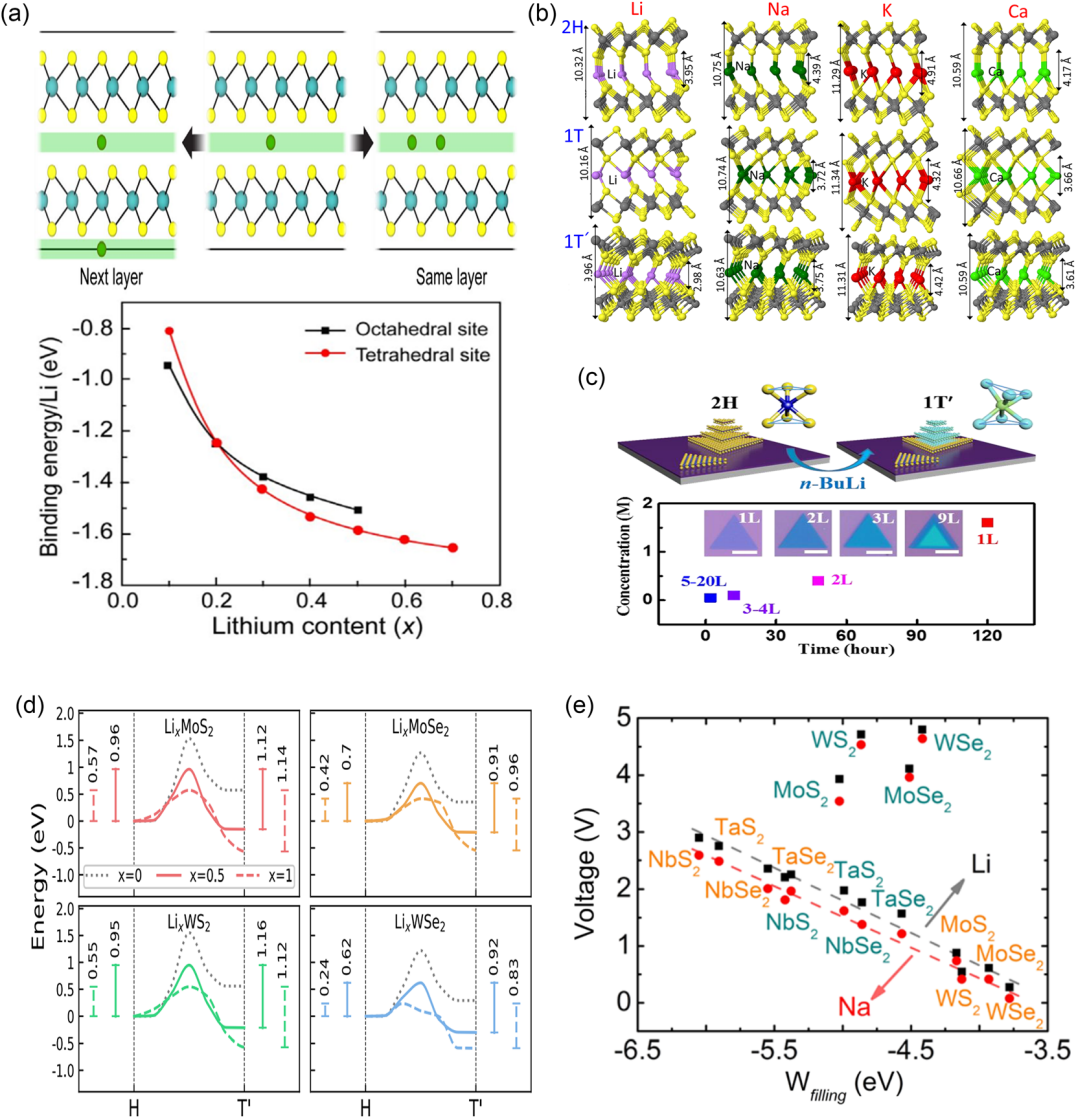
a) Schematic of the possible sequential intercalation behaviors of Li ions (top). The binding energy of Li as a function of Li content during sequential in‐plane lithiation at octahedral (black) and tetrahedral (red) sites (bottom).^[^ ^375^ ^]^ Reproduced with permission.^[^ ^375^ ^]^ Copyright 2020, Wiley‐VCH. b) Side views of Li‐, Na‐, K‐, and Ca‐intercalated 2H‐, 1T‐ and 1T′‐MoS_2_ bilayers.^[^ ^371^ ^]^ Reproduced with permission.^[^ ^371^ ^]^ Copyright 2021, Optical Society of America. c) Schematic of the layer‐dependent phase transition of MoS_2_ flakes (top). Critical phase transition conditions for MoS_2_ flakes depending on the number of layers (bottom).^[^ ^378^ ^]^ Reproduced with permission.^[^ ^378^ ^]^ Copyright 2018, American Chemical Society. d) The energy difference and barrier between the H and T phases of Li_*x*_TMX_2_ (TM = Mo, W; X = S, Se) for selective *x*. The segments on the left and right sides indicate the transition barrier height from the H to T and T to H phases, respectively.^[^ ^376^ ^]^ Reproduced with permission.^[^ ^376^ ^]^ Copyright 2019, American Physical Society. e) The relation between the energy gained for electrons to transfer into empty states of TMX_2_ (W_filling_) and the voltage of the Li–Na intercalation.^[^ ^384^ ^]^ Reproduced with permission.^[^ ^384^ ^]^ Copyright 2017, American Chemical Society.

Intercalation can play a crucial role in introducing another phase into vdW TMDs. Somoano et al.^[^ ^348^ ^]^ and a few pioneering researchers^[^ ^83^, ^349^ ^]^ demonstrated that the interaction of alkali metals (Li, Na, K, Rb, and Cs) into various TMDs^[^ ^348^ ^]^ facilitates the transition from the 2H to 1T phase. Such intercalations are known to cause additional reactions between TMDs and alkali compounds. To date, various alkali metal compounds, including alkali metal butyl,^[^ ^139^, ^140^, ^168^, ^350^, ^351^, ^352^, ^353^, ^354^, ^355^, ^356^, ^357^ ^]^ alkali‐metal‐ammonia,^[^ ^358^, ^359^, ^360^, ^361^, ^362^ ^]^ alkali/alkali halide mixture,^[^ ^66^, ^358^, ^363^, ^364^ ^]^ and pseudo‐alkali metallocenes,^[^ ^365^, ^366^ ^]^ have been introduced. Among them, alkali‐metal butyl‐based compounds (n‐BuLi,^[^ ^139^, ^140^, ^350^, ^351^, ^353^, ^354^, ^355^, ^356^ ^]^ t‐Buli,^[^ ^352^ ^]^ and LiBH_4_^[^ ^83^, ^357^ ^]^) involve considerably cleaner procedures than the other compounds. After separating the TMDs using the alkali compounds, a further hydrolysis process is applied to cleave each layer apart,^[^ ^66^ ^]^ which can be accelerated further using sonication,^[^ ^367^ ^]^ milling,^[^ ^368^ ^]^ or electrochemical treatment.^[^ ^369^, ^370^ ^]^ Numerous theoretical and experimental studies have been performed to investigate the intercalation mechanism.^[^ ^69^, ^72^, ^271^, ^371^, ^372^, ^373^, ^374^, ^375^, ^376^ ^]^ With respect to multilayer 2H‐MoS_2_, Parida et al.^[^ ^375^ ^]^ theoretically predicted that Li ions preferentially occupy the octahedral sites until the concentration of inserted Li reduces below approximately 30%. Subsequently, the remaining tetrahedral sites are filled by the excessive Li ions. After all available sites in one vdW gap are filled, the additional insertion is promoted by reducing the binding energy against the inclusion of Li until all tetrahedral sites in the interlayer are filled. As a layer of 2H‐MoS_2_ is sandwiched by two lithiated vdW gaps, the spontaneous transformation to the 1T phase is favored (**Figure** **10**a). Furthermore, the intercalation behavior can be extended to other ions, such as Na, K, and Mg, in multilayer TMDs. Shuai et al.^[^ ^377^ ^]^ reported that Li, Na, and Mg ions exhibit a preference for binding to the octahedral sites of 2H‐MoS_2_, with both the intercalation energy and binding energy decreasing in the order of Li < Mg < Na. The results indicate that the intercalation of larger monovalent Na is more facile than that of the smaller divalent Mg. Moreover, Mehmood et al.^[^ ^371^ ^]^ examined the lattice distortion of TMDs considering different intercalation atoms (M = Li, Na, K, and Ca) and reported that structural changes reduce the work function by facilitating the charge transfer (Figure 10b). These results are consistent with those reported by Gao et al.,^[^ ^65^ ^]^ which indicated that Li–Na intercalation is equivalent to the electron injection, causing a phase transition owing to the reduction in the energy barrier through charge injection (Figure 3f). Sun et al.^[^ ^378^ ^]^ determined that the critical electron injection concentration and the time required for the phase transition increase with the decrease in TMD thickness. Recently, Ryu et al.^[^ ^379^ ^]^ reported a similar trend in the MoTe_2_ phase transition, where the phase transition occurred by heating while encapsulated in hBN. The 2H‐T_d_ phase transition temperature is higher in the thinner MoTe_2_. The results imply that the phase transition encounters more difficulties in vdW TMDs with fewer layers (Figure 10c). Although this hurdle can be partially circumvented using the aforementioned methods,^[^ ^367^, ^368^, ^369^ ^]^ high‐yield production of phase‐controlled TMDs requires further research. Furthermore, Patil et al.^[^ ^376^ ^]^ calculated the energetic pathways of various single‐layer TMX_2_s (where TM = Mo, W; X = S, Se) as the function of inserted Li. They reported that the insertion of Li commonly decreases the energy barrier for the 2H‐to‐1T phase transition, increasing the energy of 2H higher than that of 1T above a certain critical concentration (Figure 10d). However, as indicated in the figure, the kinetic energy barrier remains above the level of thermal noise, indicating that the phase transition to 1T is not feasible through thermal agitation alone. Therefore, additional assistance^[^ ^367^, ^368^, ^369^, ^370^ ^]^ is required to trigger the phase transition.

Kan et al.^[^ ^72^ ^]^ demonstrated the phase transition trajectory in 1H‐MoS_2_ based on the electron transfers from the intercalated alkali Li. 1H‐MoS_2_ begins to deform to the 1T phase when the intercalated Li is greater than ≈20%; the stability becomes more solidified with the increase in Li concentration. Finally, the 1T″ phase becomes achievable at ≈100% of Li concentration. During the extraction process, the 1T″ phase reverts to the 1T′ phase, and eventually to 1H‐MoS_2_ in its fully delithiated state. Peng et al. achieved a high yield of 1T′‐MoX_2_ monolayers with over 97% phase purity and an unprecedentedly large size of up to tens of micrometers through Li intercalation.^[^ ^380^ ^]^ Zhang et al.^[^ ^381^ ^]^ reported that intercalation through the surface is more reversible and stable than that through the edge state. Based on the density functional theory (DFT) calculation, they exhibited that the energy barrier can be significantly reduced when alkali ions penetrate the Mo vacancies on the 2H‐MoS_2_ surface.

Additionally, the intercalation‐induced energy difference between different phases has been investigated for various TMDs.^[^ ^382^, ^383^ ^]^ Yang et al.^[^ ^382^ ^]^ determined that Na_*x*_TiS_2_ and Na_*x*_NbS_2_ do not undergo a phase transition and retain their structures regardless of Na adsorptions. Fan et al.^[^ ^384^ ^]^ reported that the alkali ion intercalation is energetically more favorable in group‐VI TMDs in comparison with NbX_2_. They further showed that the energy gain for electron injection into an empty state can describe the intercalation‐dependent phase behavior of TMDs. The interlayer spacing of vdW TMDs can be electrochemically controlled by varying the lithiation voltage^[^ ^370^ ^]^ (Figure 10e). Recently, Wu et al.^[^ ^197^ ^]^ fabricated a 2H‐1T homojunction on monolayer TaS_2_ using the Li‐solution process. The atomically sharp interface and well‐aligned electronic band structure were achieved with clear lithiation domain boundaries by precisely manipulating the lithiation time.

#### 5.2.2 Other Intercalation Species

Apart from alkali metals, various charge dopants have been developed as intercalation species because electron doping is known to drive phase conversion in TMDs. First, transition metals (V, Cr, Mn, Fe, Co, Ni, and Cu)^[^ ^358^, ^385^, ^386^, ^387^, ^388^, ^389^ ^]^ have been considered to tailor the phase of TMDs as they can effectively donate charges to TMD crystals. Specifically, the intercalation of 3* d*‐transition metal^[^ ^358^, ^385^, ^386^, ^387^, ^388^, ^389^ ^]^ not only changes the phase of TMDs but also often presents an interesting magnetic moment owing to their unique 3* d*‐orbital electrons.^[^ ^390^ ^]^ For instance, Fe‐intercalated 2H‐TaS_2_ and 1T‐TiS_2_ possess vertical ferromagnetic ordering with substantial magnetic resistance because of the unquenched orbital magnetic moments of Fe,^[^ ^391^, ^392^ ^]^ whereas other Fe‐intercalated TMDs possess the antiferromagnetic order, as observed in Fe_*x*_TaS_2_.^[^ ^390^ ^]^ This intriguing phenomenon is based on the Ruderman–Kittel–Kasuya–Yosida (RKKY) type Fe–Fe interactions in different *d*‐orbital electronic configurations.^[^ ^390^ ^]^ Cava et al.^[^ ^393^ ^]^ reported that the intercalation of Cu into TiSe_2_ induces superconductivity, establishing the correlation between CDW and the transition to superconductivity. Additionally, Zhao et al. analyzed the self‐intercalation of Ta into vdW TaS_2_ crystals and reported that even the intercalation of nonmagnetic atoms can generate magnetism.^[^ ^394^ ^]^

Alternatively, organic molecules (amines, pyridine, and ammonia)^[^ ^358^, ^365^, ^395^, ^396^, ^397^ ^]^ have been used for phase engineering owing to their charge injection effect. They can afford a wide tuning range of interlayer spacing to vdW TMDs owing to their diverse sizes and structures.^[^ ^398^ ^]^ This weakens the interlayer coupling, making them electrically behave more like isolated single layers, eventually enabling the adjustment of the interlayer electron–phonon coupling.^[^ ^56^ ^]^ Studies have also reported that the intercalated ammonium ions^[^ ^359^, ^399^ ^]^ or hydrazine^[^ ^400^ ^]^ hydrate can assist in the stabilization of the 1T phase in MoS_2_ and WS_2_. Zhang et al.^[^ ^401^ ^]^ reported that the mixing of liquid ammonia with K ions can induce both superconductivity and polymorphism. Furthermore, they exhibited that the inserted concentration of K drives both structural and superconductivity phase transitions. Therefore, superconductivity in MoS_2_ can be initiated from all three phases (2H, 1T, and T′ phases); however, the critical temperatures of superconductivity are significantly affected by the type of phase.

#### 5.2.3 Intercalation Methods

As mentioned earlier, solutions with diverse alkali metals dissolved in them have been used in wet‐chemical methods, considering the relative atomic size, molecular size, and electronegativity of the intercalants compared with the target TMD. Therefore, apart from alkali metals, co‐intercalants such as ammonia and metal‐amides are inserted into the interlayer space of target TMDs. Gamble et al.^[^ ^398^ ^]^ achieved a large interlayer spacing greater than ≈5 nm by inserting n‐octadecylamine into TaS_2_, enabling the emergence of superconductivity. When compared with the method involving liquid ammonia, that with n‐BuLi^[^ ^139^, ^140^, ^350^, ^351^, ^353^, ^354^, ^355^, ^356^ ^]^ provides cleaner surfaces, rendering it a more favorable option. However, the yield for pure 1H‐ or 1T′‐microflakes is not sufficiently high, and their metastability often hinders the utilization of the entire surface area for active sites.^[^ ^102^, ^143^ ^]^ The zero‐valent transition metals (Ag, Au, Cu, Co, Fe, Ni, In, and Sn) have also been used for the disproportionation redox reactions or hydrazine reduction under solution‐based environments. For instance, TiS_2_, NbSe_2_, MoSe_2_, and Bi_2_Se_3_ exhibit significant changes driven by the deintercalation of Cu/Sn/Hg in various aspects.^[^ ^402^, ^403^ ^]^ Moreover, this disproportionation technique does not require drastic changes in the host vdW TMD to enable a high degree of intercalation. Therefore, it can be advantageous in terms of reversibility as the crystal is not excessively damaged.

Finally, the electrochemical intercalation of metal ions is typically performed in an electrochemical cell combined with alkali‐metal (anode), vdW TMD (cathode), and metal ion‐contained electrolyte (intercalant). This process promotes the exfoliation of individual vdW layers from their bulk counterparts in large quantities. As either cations or anions are electrochemically intercalated into the vdW TMDs, the de‐intercalation process can be adjusted by controlling the polarity and magnitude of the applied voltage.^[^ ^77^, ^143^, ^189^ ^]^ Unlike the zero‐valent intercalation method, this is derived from the change of redox state in the host vdW TMDs.^[^ ^361^ ^]^

### 5.3 Electron Injection

Apart from the ion intercalation, the phase of TMDs can be influenced by electrons. As depicted in Figure 7,^[^ ^263^, ^277^ ^]^ the energy barrier required to activate a structural phase transition in TMDs can be influenced by the electron occupation of the orbital, which in turn can impact the final state of TMDs. Therefore, phase engineering can be achieved by directly injecting charges into TMDs, using interfacial electrons, electron bombardment, or electrostatic field. Although ion irradiation can be considered a potential tool for phase engineering, its applicability is relatively limited, except for several plasma cases,^[^ ^404^, ^405^ ^]^ owing to the risk of excessive defect generation.

#### 5.3.1 Electron Doping Through Interfacial Contact

In comparison with the surface chemical treatment, charge transfer resulting from the alignment of bands between contacting materials is a more effective method for performing high‐density doping in phase engineering.

**Figure 11:**
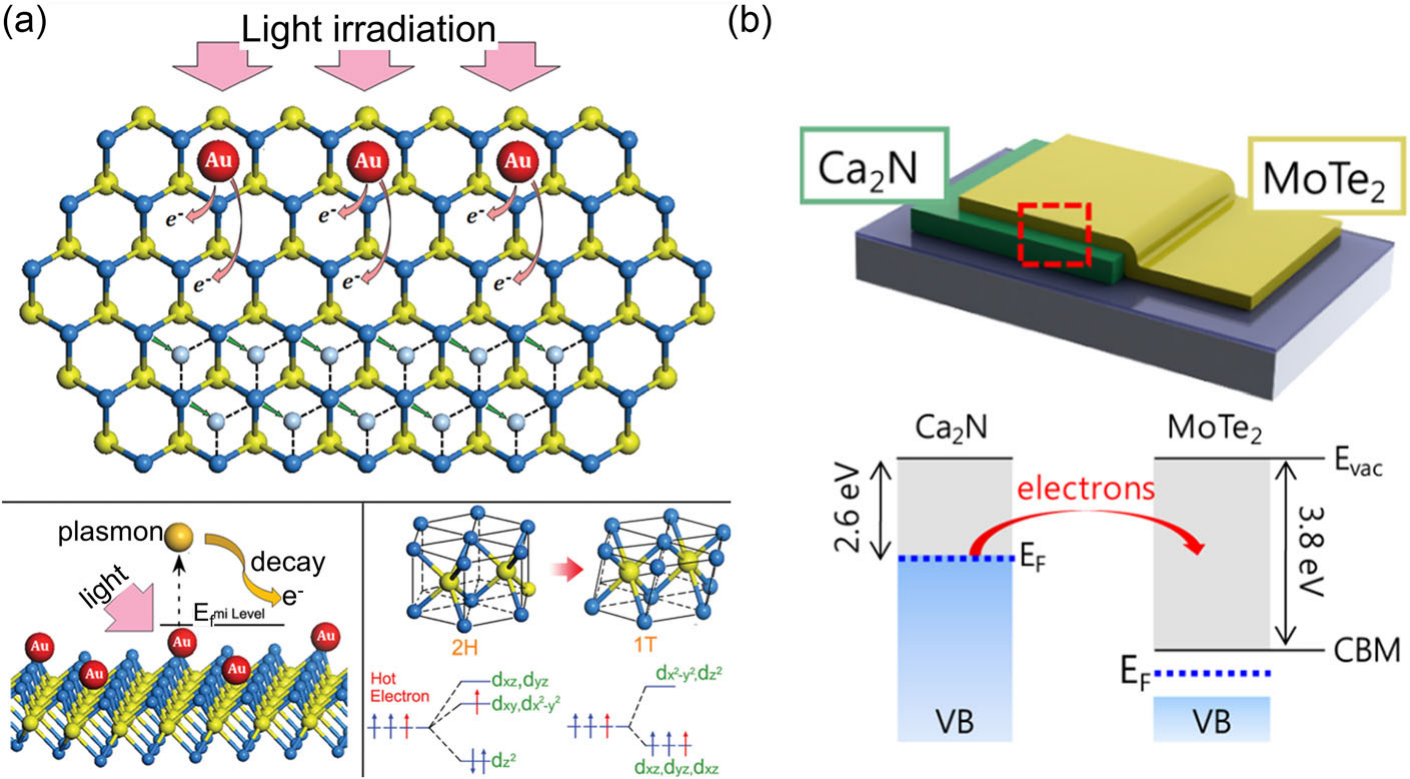
a) Illustration of phase engineering in single‐layer MoS_2_ using hot electrons generated from Au‐nanoparticle (top). The Au‐nanoparticle plasmon decay into hot electron–hole pairs with high energy above the Fermi level (bottom‐left). The transition between the 2H and 1T phases caused by the splitting of the Mo 4*d*‐orbitals caused by the crystal field (bottom‐right).^[^ ^406^ ^]^ Reproduced with permission.^[^ ^406^ ^]^ Copyright 2014, Wiley‐VCH. b) Illustration of the MoTe_2_/[Ca_2_N]^+^·e^−^ vertical heterostructure. Fermi electrons in Ca_2_N exhibit a substantially higher chemical potential than those in MoTe_2_ at the MoTe_2_/[Ca_2_N]^+^·e^−^ interface.^[^ ^408^ ^]^ Reproduced with permission.^[^ ^408^ ^]^ Copyright 2017, American Chemical Society.

If hot electrons are generated by surface plasmon at the interface between MoS_2_ and a metal with low work function, they can be transferred to MoS_2_ through plasmon‐assisted energy transfer. Consequently, the electronic properties of MoS_2_ drastically change owing to the Fermi level modulation caused by the doping of charges from the metal side. Kang et al.^[^ ^406^ ^]^ reported that a reversible phase transition can occur in single‐layer 2H/1H‐MoS_2_ coated with Au nanoparticles because the generated hot electrons can be easily transferred to the conduction band of MoS_2_ owing to the low Schottky barrier height. Therefore, the doping of MoS_2_ destabilizes its lattice, resulting in a local structural transition to the 1T phase and a reconfiguration of its *d*‐orbital state (**Figure** **11**a). Initially, this type of local transition is temporally stable and tends to revert to 1H. However, the injection of numerous hot electrons ultimately stabilizes the 1T phase permanently. Moreover, photoluminescence analysis indicated that electron doping acts as a driving force for phase transition in MoS_2_, depending on both light intensity and area. A subsequent study demonstrated that the transition from 1H to 1T phase in MoS_2_ significantly enhances the efficiency of hydrogen evolution reaction (HER) with the plasmonic hot electron, increasing hydrogen production.^[^ ^407^ ^]^

Kim et al.^[^ ^408^ ^]^ reported that the phase transition is triggered when MoTe_2_ comes in contact with a novel layered electrode ([Ca_2_N] + e). Similar to the previously reported results on hot electrons, the authors interpreted that the large difference in work function between Ca_2_N and MoTe_2_ enables the movement of electrons from Ca_2_N to MoTe_2_, with the doping length being longer than the conventional depletion width of the metal–semiconductor interface (Figure 11b).

#### 5.3.2 Electron Beam Irradiation

**Figure 12:**
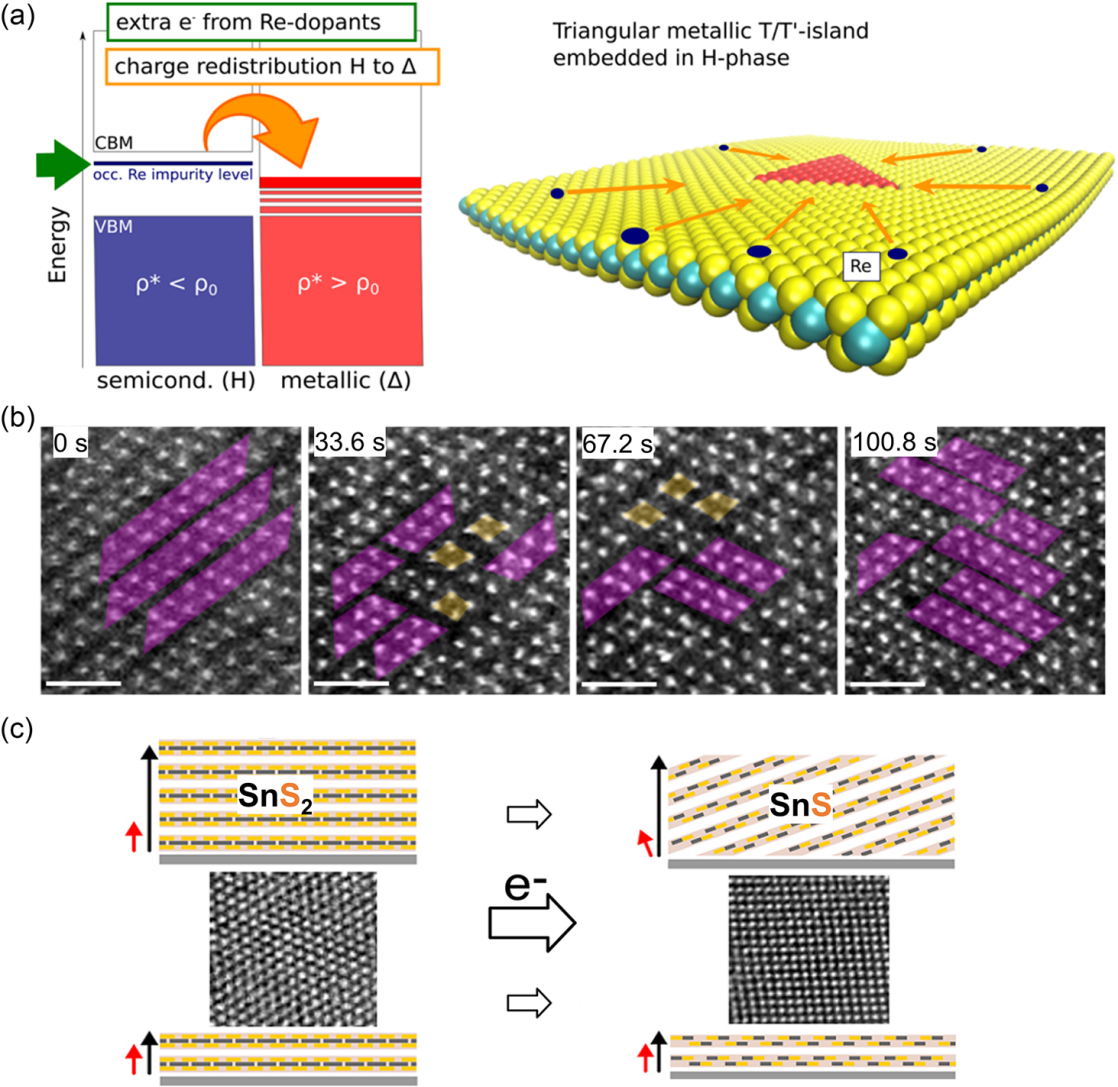
a) Schematic of charge transfer at the interface where electrons from Re‐dopant impurities can partially move from the conduction band edge (CBE) to the vacancy‐related states. The triangle region of the metallic 1T′ phase becomes energetically favorable over the reference configuration with the vacancy line.^[^ ^410^ ^]^ When exposed to an electron beam, the charge redistribution is further promoted, resulting in a higher electron density within the 1T′ phase island. Reproduced with permission.^[^ ^410^ ^]^ Copyright 2017, American Chemical Society. b) Series of high‐angle annular dark‐field (HAADF)‐STEM images showing the reorientation of the zigzag chains in single‐layer WS_2_.^[^ ^357^ ^]^ Reproduced with permission.^[^ ^357^ ^]^ Copyright 2016, American Chemical Society. c) Schematic of rhombohedral layered SnS_2_ that can be transformed into highly anisotropic orthorhombic layered SnS using an electron beam.^[^ ^415^ ^]^ Reproduced with permission.^[^ ^415^ ^]^ Copyright 2016, American Chemical Society.

High‐energy electron beam irradiation can cause structural transformations through knock‐on collisions that can lead to atom displacement, bond breaking, or local charge build‐up. As demonstrated by Lin et al.,^[^ ^75^ ^]^ the incident electron beam from TEM can induce a phase transition in 1H‐MoS_2_ via the electron beam bombardment, ultimately leading to the lattice‐plane gliding. The authors determined that there exists a threshold electron dose below which the phase transition does not occur prior to the formation of intermediate states. The phase conversion can be initiated if the electron dose surpasses the threshold, and the transformed area increases with a higher electron dose. Furthermore, their analysis demonstrated that the kinetics can be improved by thermal treatment and Re‐doping. However, despite the surprising result, because it was reported that vacancies in MoS_2_ tend to cluster and form linear shapes resulting in a significant strain in the atomic sheet, further explanations should be provided considering the strain effect.^[^ ^409^ ^]^ Kretschmer et al.^[^ ^410^ ^]^ used first‐principle calculations to analyze the stability of phases in MoS_2_ that contains charge, strain, and defective vacancies. They suggested that a combination of factors, including strain, vacancies, local charging, Re impurities, and electronic excitations, is the key to inducing the phase transitions observed in MoS_2_ under electron beam irradiation rather than solely attributing them to a single factor. Therefore, the authors proposed a scenario wherein vacancies are created by the knock‐on collisions of electron beam clusters into defect lines at high temperatures, leading to mechanical strain and the formation of the 1T′ phase through the gliding of the sulfur plane. They also reported that the presence of Re impurities and electron excitation renders the 1T′ phase regions energetically more favorable (**Figure** **12**a). To date, various studies have been conducted using the in situ TEM for other TMX_2_s as well. Amara et al.^[^ ^411^ ^]^ reported that electron beam irradiation onto single‐layer WS_2_ can induce structural evolution along the one‐dimensional (1D) zigzag chains (Figure 12b). The metal–chalcogen bond can be weakened owing to the charge build‐up during STEM observation, resulting in the emergence of tetramer clusters and zigzag chains with a new orientation. Particularly, the TEM analysis of single‐layer MoTe_2_ indicated that point defects in one Te layer tend to form single vacancy lines, whereas defects in both Te layers predominantly result in extended structures composed of column Te vacancies, including rotational trefoil‐like defects.^[^ ^412^ ^]^ This type of defect dynamics can be significantly suppressed when MoTe_2_ is encapsulated in graphene layers, forming a quantum dot or wired structure, associated with defect ordering.^[^ ^148^ ^]^ Unlike TMDs comprising multiple competing phases with identical compositions, other vdW nanosheets can stabilize their phases by adjusting their stoichiometry. For instance, tin‐based chalcogenides are known to exhibit stable phases in both Sn^4+^ (SnS_2_ and SnSe_2_) and Sn^2+^ (SnS and SnSe) ions, where the variations dependent on the oxidation state impact the electronic band structure and lattice properties, such as bandgap or anisotropy.^[^ ^413^, ^414^ ^]^ Stutter et al.^[^ ^415^ ^]^ performed in situ TEM analysis for SnS_2_ and SnSe_2_ under electron beam irradiation (Figure 12c). They reported that the electron beam can eliminate the chalcogen atoms in a controlled manner at both room temperature and elevated temperatures. This resulted in the structural conversion to highly anisotropic orthorhombic layered SnS or SnSe. Theoretical calculations have determined that S/Se vacancies can be thermodynamically arranged into ordered lines, resulting in the conversion of SnS_2_/SnSe_2_ through an intermediate Sn_2_S_3_/Sn_2_Se_3_ state. Additionally, microwave plasma treatment has been reported to convert 2H‐MoS_2_ to 1T‐MoS_2_.^[^ ^404^, ^405^ ^]^ This phenomenon can be attributed to the momentum transfer from the plasma ions to the MoS_2_, where the chalcogen plane eventually results in sliding through the phase transformation.

#### 5.3.3 Electrostatic Gating

**Figure 13:**
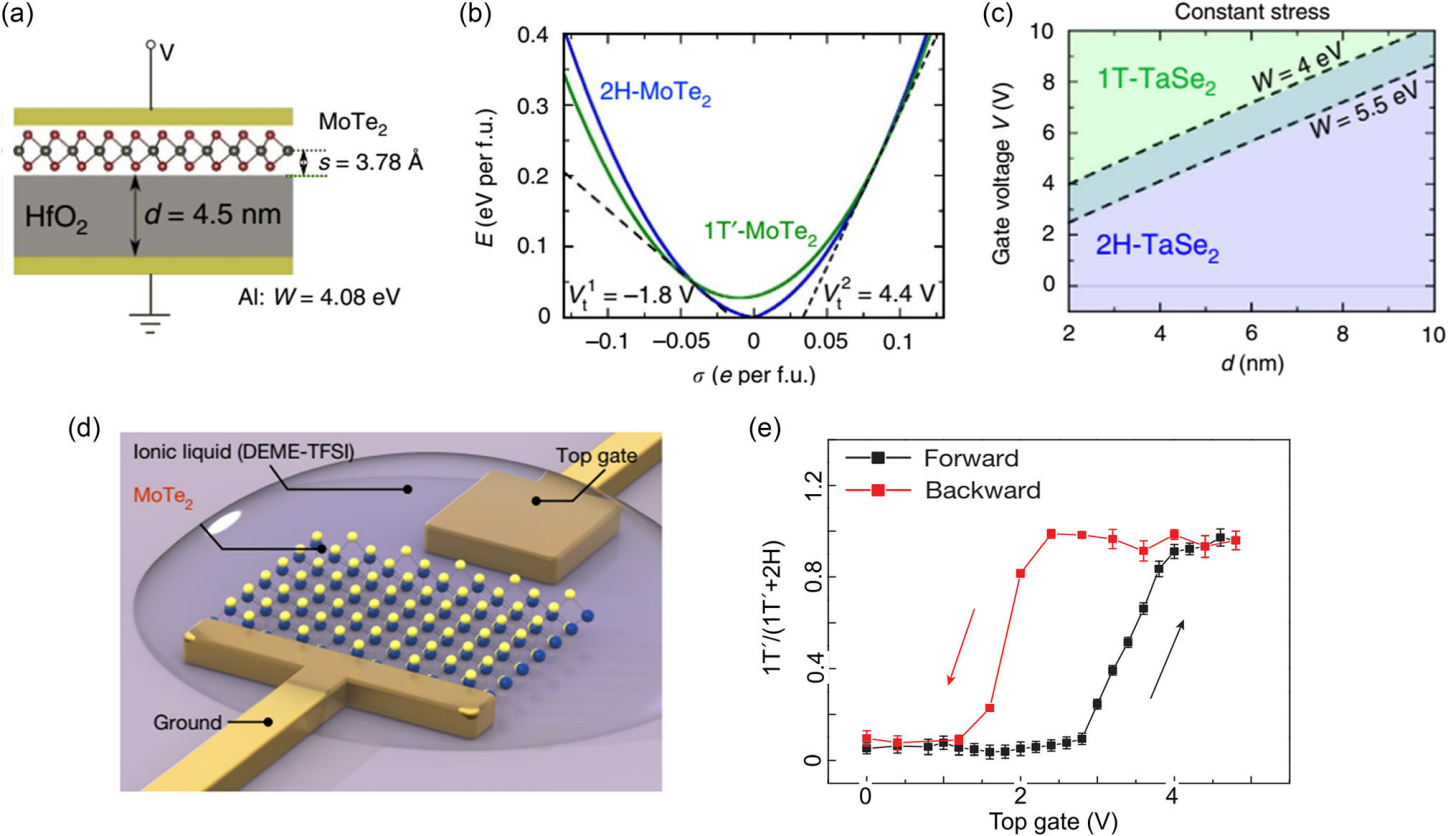
a) The capacitor schematics comprising a single layer of MoTe_2_ deposited on dielectric HfO_2_.^[^ ^79^ ^]^ b) The total energy of the capacitor plotted as a function of charge density on single‐layer MoTe_2_.^[^ ^79^ ^]^ c) Phase control of single‐layer TaSe_2_ through gate voltage and dielectric thickness at constant stress.^[^ ^79^ ^]^ a–c) Reproduced under the terms of the CC BY license.^[^ ^79^ ^]^ Copyright 2018, The Authors, published by Springer Nature. d) Schematics and measurement configuration of single‐layer MoTe_2_ field‐effect transistor.^[^ ^168^ ^]^ e) Gate‐bias‐dependent Raman intensity ratios in single‐layer MoTe_2_.^[^ ^168^ ^]^ d,e) Reproduced under the terms of the CC BY license.^[^ ^168^ ^]^ Copyright 2017, Macmillan Publishers Limited, part of Springer Nature.

Zhuang et al.^[^ ^70^ ^]^ determined that both n‐type and *p*‐type doping significantly decrease the energy difference between 1H and 1T for MoS_2_ and other single‐layer TMDs. The authors suggested that the charge doping effect also affects the energy barrier from 1T to 2H, rendering the transition reversible. Therefore, electrostatic gating has been tested as a doping control method. As theoretically expected for MoTe_2_,^[^ ^79^, ^416^ ^]^ the phase stability can be directly adjusted using the external gate bias, which can be attributed to the total energy difference between TMD phases that varies with the charge density. Li et al.^[^ ^79^ ^]^ examined the phase control behavior via electrostatic gating for MoS_2_, MoTe_2_, Mo_*x*_W_1−*x*_Te_2_, and TaSe_2_. They reported that the gate bias of voltage with several degrees of magnitude can drive the semiconductor‐to‐metal phase transition in monolayer MoTe_2_ when using high‐k HfO_2_ as a dielectric material (**Figure** **13**a,b). By contrast, the phase transition in single‐layer 1H‐MoS_2_ requires a substantially larger charge density than that required for MoTe_2_ in terms of both constant area and force, as indicated in Figure 3g. The electric field required for the phase transition in MoTe_2_ is close to its breakdown field; therefore, the value must be reduced for practical applications. The authors verified that the phase transition can be made more feasible by obtaining an alloy, such as Mo_*x*_W_1−*x*_Te_2_, through the doping of W atoms, which decreases the threshold voltage for the transition. The authors further reported that TaX_2_ remains metallic in both 2H‐ and 1T′ phases without causing significant changes in the electrical properties even when the phase transition is triggered by the electric field (Figure 13c). However, Kim et al.^[^ ^417^ ^]^ observed that the 1H phase is formed on 1T‐TaS_2_ owing to a large bias in the STM tip. A subsequent STM analysis near the phase boundary suggested that the surface *S* atomic sheet experiences perturbation and displacement caused by the STM tip bias. Moreover, Wang et al.^[^ ^168^ ^]^ recently induced a reversible phase transition between 1H and 1T′ in single‐layer MoTe_2_ by employing ionic liquid gating. They analyzed the presence of the hysteretic loop in Raman spectra and determined that the phase transition can be reversed by increasing or decreasing the gate bias voltage (Figure 13d,e).

### 5.4 Mechanical Stress–Strain Engineering

#### 5.4.1 Theoretical Expectations

**Figure 14:**
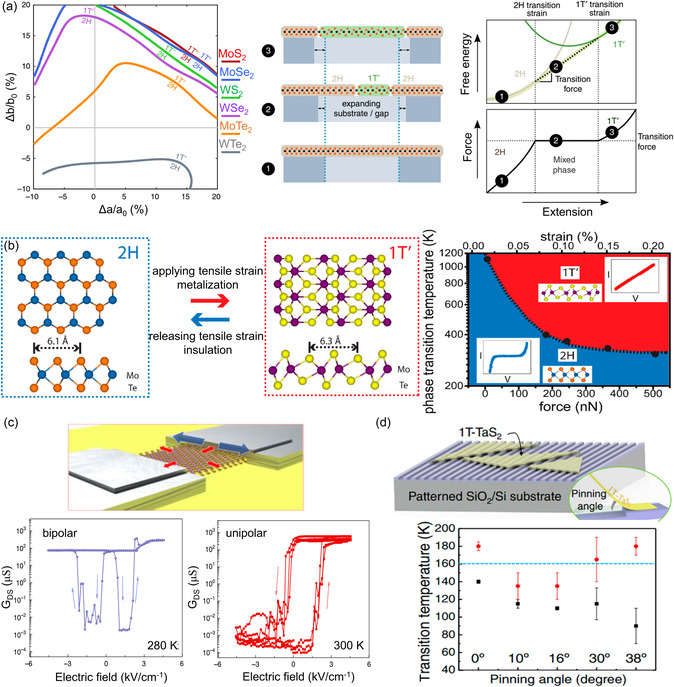
a) The energy contours between 2H and 1T phases under various rectangular lattice constants *a* and *b* (left). Phase coexistence under an applied force or extension (middle and right).^[^ ^76^ ^]^ Reproduced under the terms of the CC BY license.^[^ ^76^ ^]^ Copyright 2014, Springer Nature Limited. b) Polymorph of MoTe_2_ controlled by the in‐plane tensile strain (left). Temperature–force phase diagram for semiconducting 2H and metallic 1T′‐MoTe_2_ (right).^[^ ^423^ ^]^ Reproduced with permission.^[^ ^423^ ^]^ Copyright 2016, American Chemical Society. c) The schematic of device operation under contact metal‐induced strain (top). Both bipolar and unipolar conductivity behaviors observed in the devices owing to the strain evolution (bottom).^[^ ^425^ ^]^ Reproduced under the terms of the CC BY license.^[^ ^425^ ^]^ Copyright 2019, The Authors, under exclusive license to Springer Nature Limited. (d) Schematic of 1T‐TaS_2_ on the patterned SiO_2_/Si substrate (top). Non‐commensurate to commensurate charge density wave (CCDW) transition temperatures of 1T‐TaS_2_ on the patterned substrates based on Raman evolution and pinning angle obtained from the patterned substrates.^[^ ^426^ ^]^ Reproduced with permission.^[^ ^426^ ^]^ Copyright 2017, American Chemical Society.

In addition to charge‐mediated phase engineering, strain can tune the phases in TMDs using both uniaxial and isotropic tensile methods. Johari and Shenoy theorized that TMDs are highly sensitive to both tensile and shear strains, wherein even a small amount of strain can alter their bandgap and potentially lead to a direct‐to‐indirect bandgap transition or a semiconductor‐to‐metal transition.^[^ ^418^ ^]^ Specifically, when high‐pressure techniques are required for manipulation in conventional three‐dimensional (3D) bulk materials, vdW TMD nanosheets can be easily subjected to the planar strain applied along the lateral direction owing to their 2D specifications. The authors’ prediction was experimentally verified by the following experimental results.^[^ ^419^, ^420^, ^421^ ^]^ He et al.^[^ ^420^ ^]^ demonstrated that both the direct and indirect bandgaps of MoS_2_ can be continuously tuned by applying a uniaxial tensile strain with absorption and photoluminescence spectroscopy. Manzeli et al.^[^ ^421^ ^]^ investigated the method of modulating the piezoresistivity and electrical conductivity of the suspended MoS_2_ membrane by applying mechanical force via an SPM tip. Moreover, Duerloo et al.^[^ ^76^ ^]^ presented the phase stability diagrams of Mo‐ and W‐based TMX_2_s under planar strain environmental conditions. According to the DFT calculations reported by the authors, most group‐VI TMDs are capable of undergoing the strain‐induced phase transition from 2H to 1T. However, the critical value required for triggering the transition is the largest and smallest for MoS_2_ and MoTe_2_, respectively, with the order of threshold value being MoS_2_ > MoSe_2_ > WS_2_ > WSe_2_ > MoTe_2_ (**Figure** **14**a). However, WTe_2_ exhibits a different behavior because it possesses 1T′ as the ground state under strain‐free conditions. Therefore, unlike other group‐VI TMDs, the phase transition to 2H in WTe_2_ is possible only under a compressive stress regime.

Additionally, as the substrate–TMD interaction is strongly anisotropic owing to the application of macroscopic uniaxial strain, the authors determined the anisotropy of critical deformation required for phase transition. Based on the rank order of anisotropy as MoS_2_ < MoSe_2_ < WS_2_ < WSe_2_ < MoTe_2_, the authors verified that the phase engineering in MoTe_2_ is more responsive to uniaxial strain and determined the range of tensile strain from 0.3% to 3% at room temperature. Recently, Lee et al.^[^ ^422^ ^]^ presented experimental evidence of strong anisotropy in MoTe_2_ for the 2H‐T_d_ phase transition and investigated its favorable phase transition along the *b*‐axis with laser irradiation. Using in situ pulsed heating in the TEM analysis, the authors identified the anomalous phase transition. The research also indicates that applying tensile load along the *b‐*axis (armchair) direction is a more facile approach to inducing the transition in comparison with the *a‐*axis (zigzag) direction. Moreover, research on phonon vibrations has verified that the temperature has a greater impact on the phase boundary of MoTe_2_ compared with that observed in other TMDs. Therefore, MoTe_2_ has been identified as the most relevant material feasible for strain engineering. Unlike Duerloo et al.,^[^ ^76^ ^]^ who proposed that the phase transition from 2H to 1T′ occurs through the intermediate 1T phase, Huang et al.^[^ ^74^ ^]^ suggested that the strain‐induced transition can occur through the simultaneous sliding of transition metal atom and chalcogen layers without passing through the intermediate 1T phase. This strain effect can be realized on the TMD membrane placed on a trench structure through an AFM tip, which can locally apply adjustable strain to the TMD without being affected by the substrate.^[^ ^421^, ^423^ ^]^

#### 5.4.2 Lateral Strain Effect

According to Song et al.,^[^ ^423^ ^]^ the in situ local current measurement indicated that the phase transition from 2H to 1T′ can be induced in MoTe_2_ by a small tensile strain of 0.2%, and the transition can be reversed to 2H by releasing this strain. The authors further determined that the increased strain reduced the phase transition temperature from ≈900 °C to room temperature (Figure 14b). Berry et al.^[^ ^424^ ^]^ examined the nonuniform development of strain‐induced structural phase transformations to multiphase H‐T′ structures and/or multidomain T′ structures for group‐VI TMDs. They analyzed the time‐dependent thermodynamic issue in phase engineering by estimating the transition time from 1H to 1T′ to be ≈50 s and 0.2 ns for MoTe_2_ and WTe_2_, respectively. The time scale for the 1T′‐to‐1H transition can be shortened when 1H‐domains are preexisting. Additionally, the critical strength required to initiate the phase transition is relatively high for most 2D TMDs; therefore, the intrinsic strength of 2H‐MoS_2_ is approximately 15N m^−1^, close to its maximum stress limit before breaking.^[^ ^423^ ^]^ Although the AFM tip can be advantageous in dynamically patterning localized and reversible T′‐domains, the potential accumulation of defects over repeated bending can hinder reliable strain‐driven switching.^[^ ^424^ ^]^

Unlike the AFM tip, exploiting a substrate is a more efficient strategy for applying strain to the upper TMDs on a large scale. Hou et al.^[^ ^425^ ^]^ fabricated the MoTe_2_‐based field‐effect transistor (FET) on the ferroelectric Pb(Mg_1/3_Nb_2/3_)_0.71_Ti_0.29_O_3_ (PMN‐PT). The authors achieved reversible switching between the metallic 1T′ phase and the semiconducting 2H phase via nanoscale strain engineering of MoTe_2_, wherein the strain is induced by applying a voltage across the ferroelectric substrate. They observed that unipolar or bipolar behavior can be realized by controlling the ferroelectric strain through selective electric‐field sweeping (Figure 14c). Zhao et al.^[^ ^426^ ^]^ reported that applying periodic in‐plane strain to 1T‐TaS_2_ can harness the formation of non‐commensurate and commensurate CDW, referred to as N‐CCDW and CCDW, respectively. They observed that the temperature hysteresis at the transition is significantly influenced by the pinning angle between TaS_2_ and the pattern region, indicating that the metastable CCDW phase can survive higher temperatures at larger pinning angles greater than 30° (Figure 14d). The results revealed that the patterned substrate can provide periodic strain to enable a controllable and reliable phase engineering of TaS_2_, with the potential to adjust CDW phase transitions. Additionally, Wang et al. reported that bilayer 2H‐MoS_2_ grown on patterned substrates has an adjustable bandgap that depends on the strain mode, which can be modulated from tensile to the compressive mode by varying the specific angle between the flat and patterned cone sides.^[^ ^427^ ^]^ Qi et al.^[^ ^428^ ^]^ created an atomic cobalt array that was covalently bound to the distorted 1T‐MoS_2_ nanosheets; they reported that the phase of MoS_2_ can be altered from 2H to distorted 1T by inducing strain owing to the lattice mismatch while forming Co‐S covalent bonds during the assembly.

#### 5.4.3 Pressure

**Figure 15:**
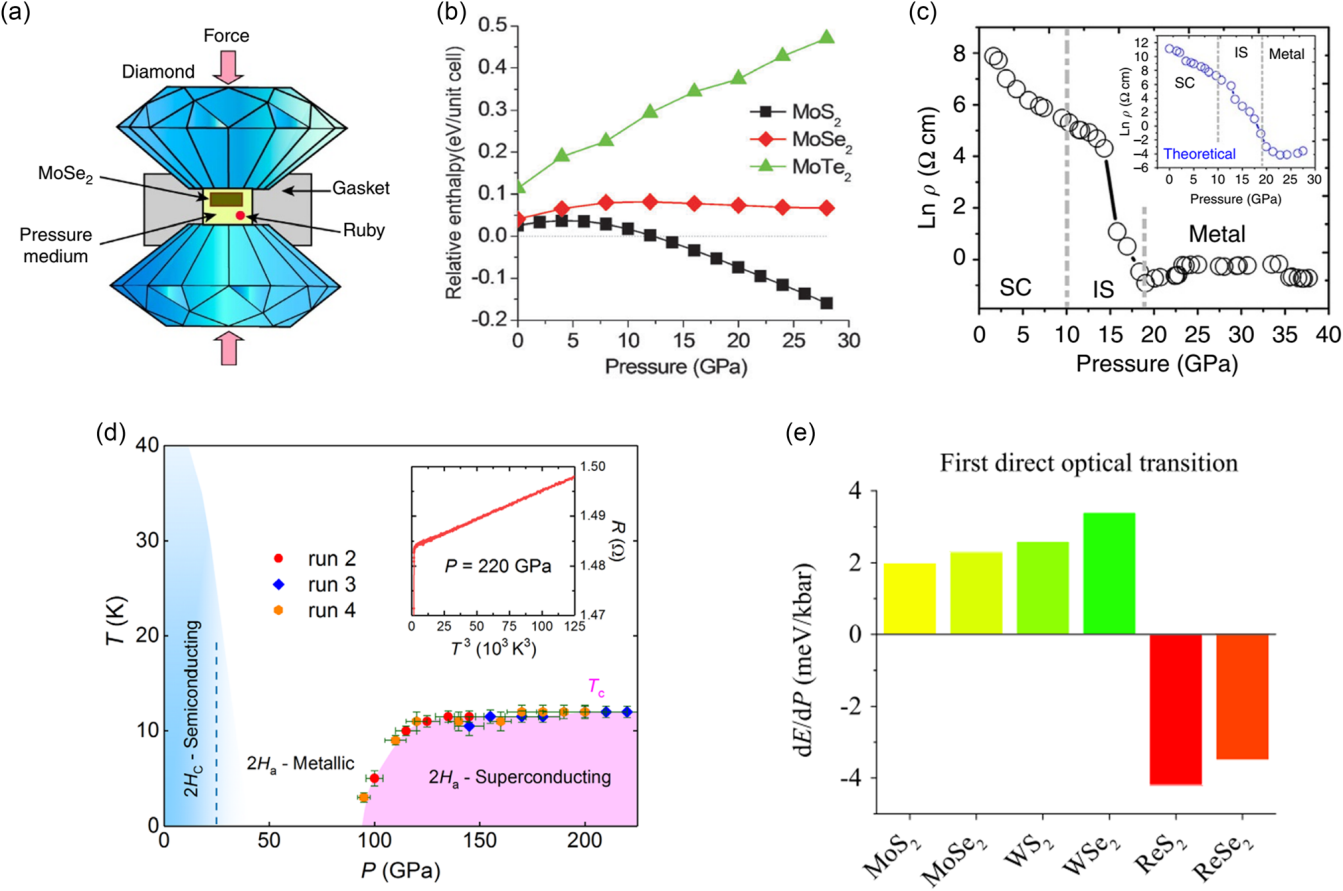
a) Schematic of the high‐pressure DAC setup.^[^ ^61^ ^]^ Reproduced under the terms of the CC BY license.^[^ ^61^ ^]^ Copyright 2015, The Authors, published by Springer Nature. b) The changes in the enthalpy difference between 2H_c_ and 2H_a_ with respect to the function of pressure for Mo‐based TMX_2_.^[^ ^64^ ^]^ Reproduced under the terms of the CC BY 3.0 license.^[^ ^64^ ^]^ Copyright 2016, The Authors, published by RSC Publishing. c) Pressure‐dependent electrical resistivity of MoS_2_ indicating three characteristic regions, namely, semiconducting (SC), intermediate state (IS), and metallic regions.^[^ ^448^ ^]^ Reproduced under the terms of the CC BY license.^[^ ^448^ ^]^ Copyright 2014, Nature Springer Limited. d) Pressure–temperature (P–T) phase diagram of 2H‐MoS_2_ indicating the boundaries among semiconducting, metal, and superconducting regions.^[^ ^429^ ^]^ Reproduced with permission.^[^ ^429^ ^]^ Copyright 2018, American Physical Society. e) Histogram indicating the pressure coefficient of the first direct optical transitions of group‐VI TMX_2_ and ReX_2_ (*X* = S and Se).^[^ ^449^ ^]^ Reproduced under the terms of the CC BY license.^[^ ^449^ ^]^ Copyright 2019, The Authors, published by Springer Nature.

As the interlayer vdW interaction is considerably weaker than the intralayer covalent bonds, interlayer spacing can be more effectively modulated than intralayer structures. Therefore, pressure continues to play a vital role in the field of strain engineering for vdW materials. Diamond anvil cell (DAC) is a widely used tool for applying high pressure to samples, facilitating the in situ investigation of the physical and electrical properties of mechanically modulated vdW materials. Furthermore, the advances in DAC equipment enable the combination of a wide range of methods, including XRD,^[^ ^429^ ^]^ Raman spectroscopy,^[^ ^430^ ^]^ Fourier transform infrared spectroscopy (FT‐IR),^[^ ^430^ ^]^ and absorption spectroscopy,^[^ ^431^ ^]^ with DAC to characterize the TMDs inside DAC based on structural, optical, and electrical measurements (**Figure** **15**a).

Dave et al.^[^ ^432^ ^]^ reported that the resistance of both MoS_2_ and MoSe_2_ gradually decreases with pressure up to 6.5 GPa without any change in their structural phase. They attributed the increased conductivity to the highly activated injection of charge carriers from the valence band to the conduction band carriers. Pressure‐dependent diverse phase transition behaviors have been observed in various vdW TMDs, such as MoS_2_,^[^ ^433^, ^434^, ^435^ ^]^ MoSe_2_,^[^ ^436^ ^]^ WS_2_,^[^ ^437^, ^438^, ^439^ ^]^ WTe_2_,^[^ ^94^, ^440^, ^441^, ^442^ ^]^ VS_2_,^[^ ^201^ ^]^ ReS_2_,^[^ ^443^, ^444^, ^445^ ^]^ and ZrX_2_.^[^ ^446^ ^]^ Among them, the pressure‐driven phase transitions from 2D to 3D structures have been observed in MoSe_2_,^[^ ^436^ ^]^ WS_2_,^[^ ^439^ ^]^ and WSe_2_,^[^ ^430^ ^]^ where Coulombic interaction between chalcogen atoms increased with pressure, eventually converting the interlayer vdW bonding to covalent‐like bonding. Furthermore, the pressure‐driven 2D‐to‐2D structural transformations have been reported to be exemplified by the commonly observed 2H_c_‐to‐2H_a_ transformations in group‐VI TMX_2_s.^[^ ^61^ ^]^ Bhattacharyya et al.^[^ ^447^ ^]^ theoretically predicted that the bandgap of bilayer group‐VI TMDs can be commonly reduced by applying a vertical compressive pressure, which eventually causes a reversible transition from a semiconductor to a metal phase at a critical pressure. The authors interpreted that the transition is associated with the movement of the VBE from the K‐point to Γ‐point as the pressure increases, ultimately approaching the Fermi level. They also proposed that for a specific TM in TMX_2_, the threshold pressure required to trigger the transition decreases as X changes from S to Te owing to weaker interactions between them. Furthermore, as Mo has a lower ionization potential than W, the authors suggested that Mo can donate charges relatively easily, thereby facilitating the X–X interaction and promoting the semiconductor‐to‐metal transition in MoX_2_ at a pressure lower than that observed in WX_2_. Similarly, Fan et al.^[^ ^64^ ^]^ theoretically calculated that the energy barrier for the phase transition from 2H_c_ to 2H_a_ increases with higher pressure as the size of the X atom increases, which differs from the usual phase transition in semiconductors. They suggested that the phase transition from 2H_c_ to 2H_a_ is practically achievable only in MoS_2_ because of the competition between vdW interlayer interaction and Coulomb force (Figure 15b). Riflikova et al.^[^ ^60^ ^]^ reported that, unlike MoS_2_, both MoSe_2_ and MoTe_2_ preserve their 2H_C_ structure even after undergoing metallization by closing their gap under high pressure. The authors predicted that MoSe_2_ and MoTe_2_ could exhibit pressure‐induced metallization at transition pressures of 28–40 GPa for MoSe_2_ and 13–19 GPa for MoTe_2_.^[^ ^60^ ^]^ Nayak et al.^[^ ^448^ ^]^ experimentally demonstrated that a structural transformation of MoS_2_ from its original 2H_c_ to 2H_a_ occurs during DAC compression, accompanied by an electronic transition from a semiconducting to a metallic state at ≈19 GPa (Figure 15c). The authors suggested that metallization is associated with the overlapping between the valence and conduction bands, caused by an increase in the interaction between the chalcogen atom layers facing each other, as the interlayer spacing decreases. Unlike typical phase transitions, this electronic transition does not involve 1T or 1T′ structures. The authors further determined that the critical pressure for the transition from a direct band to an indirect band decreases as the number of layers increases. The same authors also reported that multilayered WS_2_ undergoes a 2D‐to‐2D isostructural transition at ≈22 GPa (280 K), which was validated by the changes in mobility and resistivity, while maintaining the 2H_a_ crystal structure.^[^ ^439^ ^]^ Chi et al.^[^ ^429^ ^]^ determined that a 2D‐to‐2D isostructural phase transition can also be induced by pressure and validated that the phase transition from metallic 2H_a_ to superconducting 2H_a_ can be enhanced by increasing the pressure (Figure 15d). Interestingly, the authors observed that a large magnetoresistance of WTe_2_ is strongly suppressed by pressure, which results in superconductivity.^[^ ^442^ ^]^

Unlike group‐VI TMX_2_s, ReX_2_ exhibits larger variations under pressure owing to its in‐plane anisotropy (Figure 15e).^[^ ^449^ ^]^ The 2D‐to‐2D stacking transitions in ReS_2_ are derived by increasing the pressure. Therefore, ReS_2_ undergoes the intralayer transition at approximately 8 GPa, followed by the interlayer transition from distorted 3R to distorted 1T at ≈15.4 GPa.^[^ ^444^ ^]^ Furthermore, the transition from a distorted‐1T to distorted‐1T′ was observed at ≈7.7 GPa, followed by the semiconductor‐to‐metal transition at ≈38.5 GPa.^[^ ^443^ ^]^ The transition to a tetragonal *I*4_1_/*amd* structure was also observed at ≈90 GPa.^[^ ^445^ ^]^

### 5.5 Chemical Functionalization

Apart from intercalation, which often leads to the partial conversion of phase,^[^ ^450^, ^451^ ^]^ functional groups can be used to alter the phase of TMDs more effectively. If the functional groups are adsorbed on the surface, covalent bonding between the functional groups and TMDs can polarize the TMDs. This destabilizes the original phase and triggers a phase transformation. Thus far, a wide range of inorganic groups has been investigated to facilitate phase engineering. These groups include H,^[^ ^452^, ^453^, ^454^ ^]^ O,^[^ ^452^ ^]^ F,^[^ ^452^ ^]^ Cl,^[^ ^452^ ^]^ H_2_,^[^ ^452^, ^455^ ^]^ NH_2_,^[^ ^452^, ^455^ ^]^ NH_3_,^[^ ^452^, ^455^ ^]^ NO,^[^ ^452^, ^455^ ^]^ NO_2_,^[^ ^452^, ^455^ ^]^ CO,^[^ ^452^, ^455^ ^]^ CO_2_,^[^ ^452^, ^455^ ^]^ N_2_,^[^ ^452^, ^455^ ^]^ O_2_,^[^ ^452^, ^455^ ^]^ and H_2_O.^[^ ^452^ ^]^ Additionally, inorganic functional groups can be considered as functionalization groups, which include CH_3_,^[^ ^455^ ^]^ OCH_3_,^[^ ^455^ ^]^ CF_3_,^[^ ^455^ ^]^ C_2_H_4_INO (2‐iodo‐acetamide),^[^ ^355^, ^450^ ^]^ CH_3_I (iodomethane),^[^ ^355^, ^450^ ^]^ diazonium salts,^[^ ^355^, ^450^, ^451^ ^]^ CHCl_3_ (chloroform),^[^ ^456^ ^]^ CH_2_Cl_2_ (dichloromethane),^[^ ^456^ ^]^ C_6_H_4_Cl_2_ (*o*‐dichlorobenzene),^[^ ^456^ ^]^ C_4_H_8_O_2_ (ethyl acetate),^[^ ^456^ ^]^ C_6_H_5_CH_3_ (toluene),^[^ ^456^ ^]^ C_18_H_36_ (octadecene),^[^ ^456^ ^]^ and C_6_H_14_ (hexane).^[^ ^456^ ^]^

**Figure 16:**
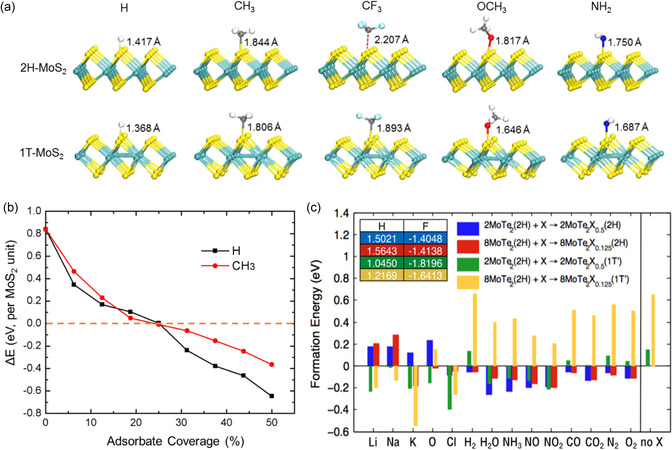
a) Structures of 2H‐ and 1T‐MoS_2_ functionalized by different radical groups: from left to right, −H, −CH_3_, −CF_3_, −OCH_3_, and −NH_2_. The bonding lengths between the sulfur and radical are indicated.^[^ ^455^ ^]^ (b) Energy difference between 1T and 2H phases in MoS_2_ as a function of the adsorption coverage of functional groups.^[^ ^455^ ^]^ a,b) Reproduced with permission.^[^ ^455^ ^]^ Copyright 2015, American Chemical Society. c) Formation energy of various molecular and atomic adsorption on single‐layer 1H‐MoTe_2_, indicating that molecular binding can energetically favor the 2H phase, whereas the binding of Li, Na, K, H, O at higher concentration, Cl, and F can potentially cause phase transition from 2H to 1T′.^[^ ^452^ ^]^ Reproduced with permission.^[^ ^452^ ^]^ Copyright 2015, American Chemical Society.

Tang et al. performed DFT calculations to analyze the phase stability and electronic properties of monolayer 2H/1H‐MoS_2_ and 1T‐MoS_2_, whose surfaces are adsorbed with various functional groups to determine the favorable adsorption sites and the corresponding adsorption energies (**Figure** **16**a). The authors determined that the adsorption of the functional groups on the surface was stronger in the case of 1T‐MoS_2_ than that observed in 2H/1H‐MoS_2_, resulting in a substantially shorter surface bonding length on 1T‐MoS_2_ compared with 2H/1H‐MoS_2_. They attributed the stronger bonding to the metallicity of MoS_2_ and the partially filled Mo 4*d* states. Furthermore, the authors demonstrated that the stability of the 1T phase is significantly enhanced as the adsorption coverage increases, surpassing that of the 2H phase. A monotonic decrease in bandgap is observed beyond approximately 25% coverage (Figure 16b). Similarly, Zhou et al.^[^ ^452^ ^]^ performed DFT calculations to investigate the formation energy of molecules and atoms adsorbed on the surface of 1H‐ and 1T′‐MoTe_2_, where adsorption of the molecules (adatoms) generally stabilizes the 1H and 1T′ phases, respectively. Based on further analysis, the authors concluded that atomic adsorption generally leads to the formation of 1T′ metallic phases, whereas molecular adsorption tends to induce 1H phases. They suggested that such phase sensitivity is associated with the small energy difference (≈31 meV f.u.^−1^) between 2H and 1T′ phases in MoTe_2_ (Figure 16c).

### 5.6 Laser Irradiation

**Figure 17:**
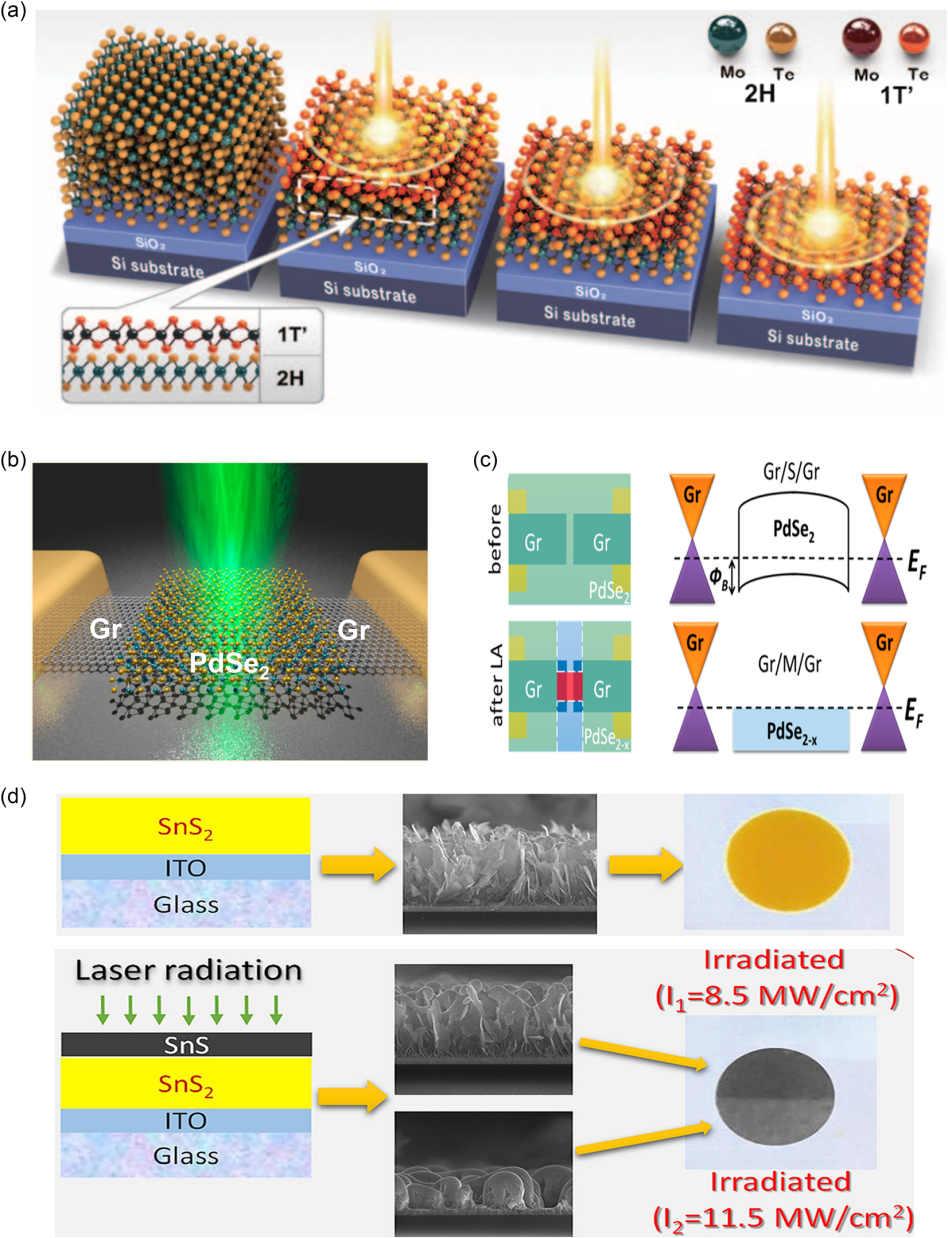
a) Schematic of the laser irradiation patterning process.^[^ ^457^ ^]^ Reproduced with permission.^[^ ^457^ ^]^ Copyright 2015, The American Association for the Advancement of Science. b) Laser annealing of a PdSe_2_ FET with graphene contacts.^[^ ^461^ ^]^ c) Structural schematics for the unmodified PdSe_2_/Gr (top) and laser‐modified PdSe_2−*x*_/Gr devices (bottom) with the corresponding energy band diagrams.^[^ ^461^ ^]^ b,c) Reproduced with permission.^[^ ^461^ ^]^ Copyright 2019, American Chemical Society. d) The evaporation of sulfur from the surface of SnS_2_, leading to the transformation to SnS and Sn_2_S_3_ phases.^[^ ^462^ ^]^ Reproduced with permission.^[^ ^462^ ^]^ Copyright 2016, Elsevier B.V.

Cho et al. reported that when multilayer 2H‐MoTe_2_ is exposed to laser illumination, an irreversible change is observed in the T′ phase, which cannot be understood simply based on the thermodynamic reactions in the phase diagram owing to its irreversibility^[^ ^457^ ^]^ (**Figure** **17**a). The DFT calculations indicated that as the concentration of Te monovacancy exceeds approximately 3% at an elevated temperature, the 1T′ phase becomes more stable than the 2H phase, which is consistent with the STEM results. The authors also created a unique junction between two different phases of the material, namely the semiconducting 2H and metallic 1T′ phases. This junction facilitated a significant reduction in resistance at the interface. Furthermore, the same research team verified that the thickness of monoclinic MoTe_2_ could be selectively reduced in specific areas in a controlled manner by using targeted laser irradiation.^[^ ^458^ ^]^ They used Raman spectroscopy to indicate that the laser‐thinning effect is related to a process referred to as thermal detachment, which is caused by local heating of up to approximately 300–400 °C. However, the transition mechanism remains debatable. In contrast to the suggestion of a Te‐vacancy‐induced phase transition, Sakanashi et al.^[^ ^459^ ^]^ reported that the improved contact resistance is caused by the Te atoms clustered during the photothermal evaporation and not by the generation of the 1T′ phase. Wang et al.^[^ ^459^ ^]^ reported that as a continuous‐wave laser warrants a long heating time for the layer thinning and phase engineering of MoTe_2_, the observed results are significantly influenced by the thermal dissipation of the substrate. Based on this, the authors argued that the accumulated heat is a primary driving force for the phase transition. Peng et al.^[^ ^460^ ^]^ proposed another explanation for the mechanism, indicating that the phase transition of single‐layer MoTe_2_ can be triggered solely by the photoexcitation of carriers. The authors explained that a structural shift can occur because the excited electrons can soften the lattice vibration through a Peierls‐like mechanism within the conduction bands.

Shautsova et al.^[^ ^461^ ^]^ reported that laser irradiation progressively increases the concentration of Se vacancies in PdSe_2_, resulting in phase modification (PdSe_2−*x*_) and the formation of Pd nanoparticles depending on the laser power. Particularly, the phase shifting is induced by Se vacancies at low laser power, whereas Pd nanoparticles are produced owing to the Se evaporation at high laser power. Moreover, the authors fabricated a PdSe_2_‐based FET and improved the device performance by adapting the laser‐ablation technique to ensure contact between Pd‐rich PdSe_2−*x*_ and graphene (Figure 17b,c). Similarly, Vozni et al.^[^ ^462^ ^]^ revealed that the laser irradiation on the SnS_2_ can modify the phase to SnS and Sn_2_S_3_ by evaporating the S‐atoms, and the composition ratio is dependent on the laser power. The authors suggested that no discernible phase separation occurs despite the chemical ratio between Sn and S monotonically depending on the laser power. This implies that the structure of SnS_2_ is not modified by the formation of secondary phases, such as Sn_2_S_3_ and SnS, or other polyphase (Sn_*x*_S_*y*_) (Figure 17d). The authors also fabricated a heterojunction with *p*‐SnS/*n*‐SnS_2_ and another with ITO/Sn_*x*_S_*y*_/Al using laser irradiation and confirmed their diodic and ohmic behavior, respectively, based on their current–voltage (I–V) curves. Additionally, encapsulation may be necessary to suppress the loss of Te by evaporation during the phase conversion of MoTe_2_. Ryu et al.^[^ ^463^ ^]^ exhibited the stable transformation of 2H‐MoTe_2_ into the 1T′ phase. This stable phase‐patterning results in the low contact resistivity (1.13 kΩ μm) in FET based on the seamless heterophase with conductive 1T′ phase.

## 6 CDW

CDW phase is a macroscopic quantum state that occurs when the electronic charge density undergoes a periodic modulation along with periodic distortions of atomic lattices. Although the phenomenon of CDW has been identified in various materials, their mechanisms are the subject of ongoing debates in different materials and dimensions. Specifically, the dimensions can be substantially confined in vdW TMD nanosheets by decreasing the number of layers, leading to more pronounced interlayer interaction effects than that observed in the bulk state. Therefore, the study of CDW can be advantageously simplified in TMD nanosheets. Furthermore, as CDW systems often exhibit unique interplays with other intriguing effects, such as superconductivity, Mott insulation, and spin properties, reducing the dimensionality to an extreme level can present these effects with more clarity and ensure convenient analysis.^[^ ^464^, ^465^, ^466^, ^467^ ^]^

### 6.1 CDW Mechanisms in vdW TMDs

As vdW TMDs have emerged as fertile hosts for inducing CDW, studies related to this aspect are indispensable. Typically, a transition to CDW is observed in group‐5 TMX_2_s (TM = V, Nb, and Ta) and a few group‐4 TMX_2_s (TM = Ti), where the structural phase can be 1T (e.g., VS_2_,^[^ ^468^ ^]^ VSe_2_,^[^ ^469^, ^470^ ^]^ VTe_2_,^[^ ^471^, ^472^ ^]^ TaS_2_,^[^ ^473^, ^474^ ^]^ TaSe_2_,^[^ ^474^, ^475^ ^]^ TiSe_2_,^[^ ^476^ ^]^ NbSe_2_^[^ ^477^ ^]^) or 2H(e.g., TaS_2_,^[^ ^478^ ^]^ TaSe_2_,^[^ ^475^, ^478^ ^]^ NbSe_2_,^[^ ^479^, ^480^ ^]^). As the critical temperature of CDW can significantly increase with dimensional confinement, metallic TMX_2_ nanosheets, namely, TiSe_2_, TaS_2_, and TaSe_2_, have become popular owing to their promising potential in various applications.

**Figure 18:**
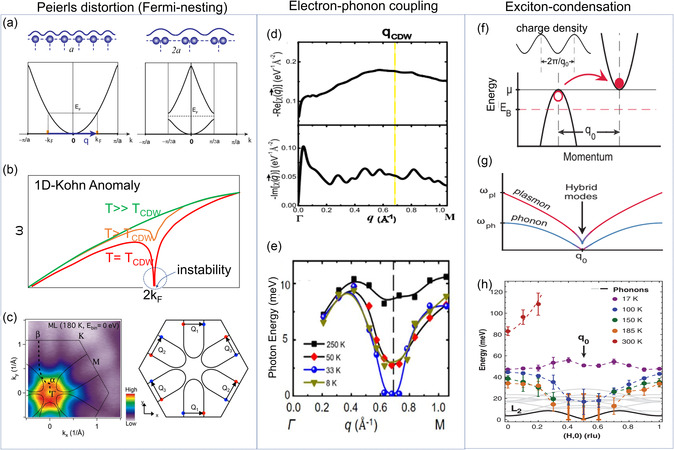
Three possible mechanisms causing the CDW transition in TMDs: a–c) Fermi surface nesting; d,e) electron–phonon coupling; and f–h) exciton condensation. a) Illustration of the Peierls phase transition of a one‐dimensional (1D) chain with lattice constant *a*, which is dimerized during the transition from the normal state to the CDW state.^[^ ^482^ ^]^ b) Nesting induced 1D‐Kohn anomaly, where phonon softening is sharply confined close to the CDW wave vector (2k_F_)q_CDW_. c) Fermi surface mapping of single‐layer VSe_2_ (left). Schematic of the Fermi surface contour of single‐layer VSe_2_ with a nesting wave vector of each pocket (right).^[^ ^482^ ^]^ a–c) Reproduced under the terms of the CC BY 3.0 license.^[^ ^482^ ^]^ Copyright 2022, The Authors, published by IOP Publishing. d) The real and imaginary parts of the susceptibility χ_0_(q) plotted from the experimental data in the Γ‐M direction.^[^ ^485^ ^]^ Reproduced under the terms of the CC BY license.^[^ ^485^ ^]^ Copyright 2008, The Authors, published by IOP Publishing. e) Acoustic phonon softening in NbSe_2_ along the Γ‐M direction for various temperatures.^[^ ^486^ ^]^ Reproduced with permission.^[^ ^486^ ^]^ Copyright 2011, American Physical Society. f) Charge density modulation induced by spontaneous exciton condensation.^[^ ^488^ ^]^ g) The hybrid mode between plasmon and phonons, which softens at T_c_.^[^ ^488^ ^]^ h) Dispersion curves of TiSe_2_ along the (1, 0) momentum direction where only the transverse L_2_ mode softens at T_C_, participating in the phase transition.^[^ ^488^ ^]^ f,g) Reproduced with permission.^[^ ^488^ ^]^ Copyright 2017, The American Association for the Advancement of Science.

To date, several conceivable types of CDWs have been reported in 2D or quasi‐2D TMD nanosheets. First, the transition to the CDW state is typically considered as the consequence of Fermi surface nesting with a doubled wave vector and a bandgap opening at Fermi energy in a quasi‐1D chain.^[^ ^481^, ^482^ ^]^ (**Figure** **18**a). When the temperature reduces below a critical temperature (T_CDW_), the atomic lattice tends to gain additional stability by inducing a periodic dimerization of lattice from *a* to 2*a*. Consequently, the halving of the Brillouin zone generates an energy bandgap at *k *= ±π/2*a* and induces electronic modulation with a wave vector of 2*k*_F_. As the Fermi surface zone‐boundary vector is connected to the nesting vector (**q** = 2*k*_F_) and is equivalent to the CDW vector, the Fermi surface nesting is proposed as a possible mechanism that leads to the emergence of CDW.^[^ ^481^, ^483^ ^]^ Kohn emphasized that this nesting result (referred to as “Kohn anomaly”) facilitates simultaneous softening of coherent lattice vibrations, referred to as phonon softening. Several TMX_2_s have indicated that their phonon energy at **q** = 2*k*_F_ becomes imaginary below T_CDW_ owing to the newly generated lattice structure. In general, imaginary frequency implies phase transition, indicating that Fermi surface nesting can lead to phonon instability. Numerous TMDs^[^ ^470^, ^474^, ^481^ ^]^, including vanadium‐based TMX_2_s exhibit imaginary phonon energy at *q* = *2k*_*F*_ below T_CDW_. At this point, the phonon frequency decreases rapidly and becomes discontinuous, which in turn leads to lattice distortion and reconstruction along with the redistribution of electron density. However, such a sharp dip (Kohn anomaly) becomes rapidly blurred and eventually disappears above T_CDW_ without an overall structural reconfiguration (Figure 18b. Duvjir et al.^[^ ^470^ ^]^ performed angle‐resolved photoemission spectroscopy (ARPES) measurement for single‐layer VSe_2_ and determined that the Fermi surface nesting was perfect (Figure 18c). Therefore, ARPES clarified that Fermi nesting in the parabolic band structure occurs between the two Fermi‐level crossing points (denoted using arrows in the figure), satisfying the Fermi‐nesting condition.

The second possible mechanism that induces CDW can be summarized as follows: CDW is dictated by electron–phonon coupling rather than Fermi nesting. Zhu et al.^[^ ^484^ ^]^ calculated the Lindhard response function using previous ARPES data for 2H‐NbSe_2_^[^ ^485^ ^]^ and plotted its real and imaginary parts separately (Figure 18d). As indicated in the figure, no apparent Fermi surface nesting is observed in either the real or imaginary parts of the susceptibility plotted from the ARPES data along the Γ‐M direction. Additionally, Weber et al.^[^ ^486^ ^]^ obtained the temperature‐dependent Kohn anomaly in the acoustic phonon branch using inelastic X‐Ray scattering (Figure 18e). The renormalized phonon dispersion was plotted along the Γ‐M direction, which exhibited phonon softening at approximately *q*_CDW_ = 0.695 Å^−1^; this indicates the correlation between CDW and the Kohn‐like anomaly. As depicted in the figure, the phonon energy reduced near the *q*_CDW_ above the transition temperature; however, it does not reach zero, similar to the observation in Fermi nesting. Furthermore, the phonon mode is damped over a wide range of Brillouin zone, unlike that observed in the case of Fermi nesting. Moreover, while a weak‐coupling CDW derived from Fermi surface nesting opens a small gap on the Fermi surface, a CDW caused by strong electron–phonon coupling creates a larger gap away from the Fermi surface.^[^ ^487^ ^]^

Another possible mechanism that causes CDW is the exciton condensation, which can be observed either in semimetals or semiconductors. Kogar et al.^[^ ^488^ ^]^ investigated the collective electronic motion in semimetal 1T‐TiSe_2_ using momentum‐resolved electron‐energy‐loss spectroscopy (Figure 18f); they determined that excitons play a vital role in forming CDWs. As observed in the dispersion curves along the (1, 0) momentum direction of TiSe_2_, only the transverse *L*_2_‐phonon mode softens at T_C_, participating in the phase transition. The energy of the electronic mode reaches zero at the nonzero momentum, indicating that the plasmon and *L*_2_ phonon interact at the transition (Figure 18g,h). This type of exciton‐induced phonon softening has been rarely reported in the Peierls distortion‐derived CDW because the metallic screening effect prevents the formation of excitons. Therefore, unlike that observed in exciton condensation, the plasmon does not reach zero in Peierls materials.

### 6.2 Characterizations of CDW in TMDs

To experimentally characterize the features of CDW, ARPES^[^ ^469^, ^470^, ^474^ ^]^ is used to observe the band structure and Fermi contour, which then enables the estimation of electronic susceptibility. Additionally, various other diffraction techniques, such as electron diffraction,^[^ ^489^ ^]^ X‐Ray diffraction,^[^ ^490^ ^]^ and neutron diffraction,^[^ ^491^ ^]^ can be used to examine the structure of CDW materials at different temperatures to identify the critical temperature required for CDW transition.

**Figure 19:**
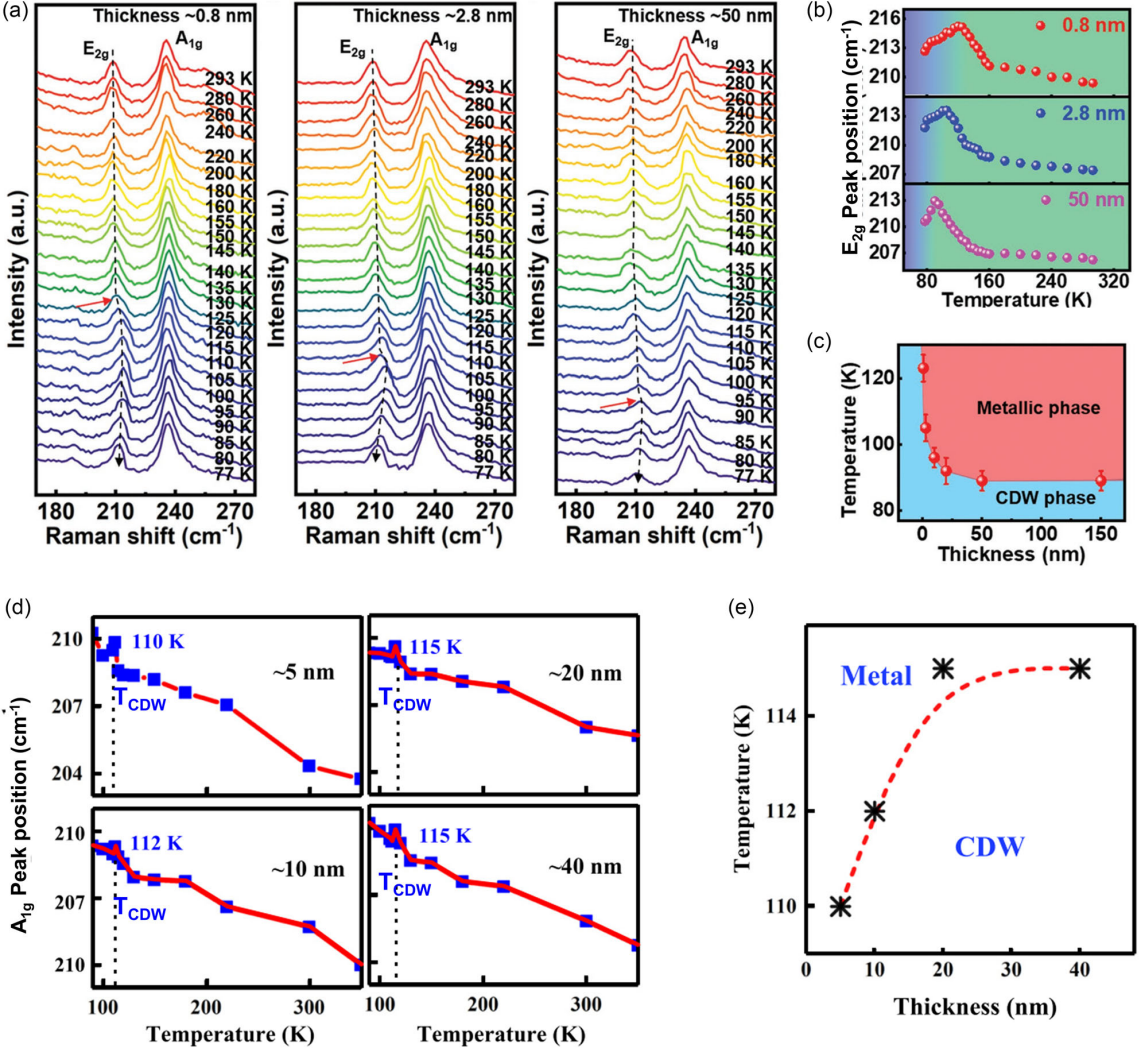
a) Variations in the Raman spectra of single layer of 2H‐TaSe_2_ with different sample temperatures.^[^ ^311^ ^]^ b) The behavior of the E_2g_ mode of 2H‐TaSe_2_ as a function of temperature considering various thicknesses.^[^ ^311^ ^]^ c) Phase diagram of the charge density wave (CDW) transition temperature of 2H‐TaSe_2_ with respect to the layer thickness.^[^ ^311^ ^]^ a–c) Reproduced with permission.^[^ ^311^ ^]^ Copyright 2018, Wiley‐VCH. d) The positions of peaks in the A_1g_ mode of 1T‐VSe_2_ with various thicknesses plotted as a function of temperature.^[^ ^492^ ^]^ e) The phase diagram of the CDW transition temperature of 1T‐VSe_2_ with respect to the layer thickness.^[^ ^492^ ^]^ d,e) Reproduced with permission.^[^ ^492^ ^]^ Copyright 2020, AIP Publishing.

Temperature‐variable Raman spectroscopy can conveniently characterize the CDW behavior of TMDs. Shi et al.^[^ ^311^ ^]^ performed Raman spectroscopy over a single‐layer 2H‐TaSe_2_ film by varying the temperature from 77 to 293 K (**Figure** **19**a,b). Interestingly, the E_2g_ mode (the in‐plane vibration of two Se atoms around a Ta atom) initially undergoes a blue shift owing to phonon hardening. However, the peak shifts back during further cooling, approximately below 125 K, which corresponds to the CDW transition. Additionally, as the thickness increases, T_CDW_ determined from the observed kinks gradually decreases as the temperature decreases from approximately 105 K (nearly 0.8 nm) to 90 K (nearly 50 nm), whereas the A_1g_ mode, corresponding to vertical vibrations, is rather insensitive to the CDW. The authors proposed that the electron–phonon coupling in 2H‐TaSe_2_ nanosheets enhances the CDW phenomenon, as indicated in the phase diagram (Figure 19c). Conversely, in 1T‐VSe_2_, the peak in the A_1g_ mode appears more pronounced in both the normal metallic and CDW phases.^[^ ^492^ ^]^ Interestingly, the peak in the A_1g_ mode corresponding to the vertical phonon vibration shifts to a higher wavenumber during the cooling process owing to phonon hardening. As the temperature decreases further from 110 to 90 K, the wavenumber in the hardened A_1g_ mode unexpectedly reduces near the critical temperature, where the metallic phase transitions into the CDW phase (Figure 19d). The phase diagram of CDW phase transition determined from the critical temperature (Figure 19e) indicates that the transition temperature of CDW gradually decreases with the decreasing thickness of VSe_2_. The presence of excess V atoms in the vdW gap between the layers may be a contributing factor affecting the formation of CDW. Moreover, Samnakay et al.^[^ ^493^ ^]^ characterized the behavior of characteristic Raman peaks in 1T‐TaSe_2_ depending on the evolution of the CDW phase. The authors observed the occurrence of several different CDW phases at different temperatures, including the commensurate CDW (CCDW), nearly commensurate CDW (NCCDW), and incommensurate CDW (ICCDW). The Raman spectra of each phase display unique characteristics.

**Figure 20:**
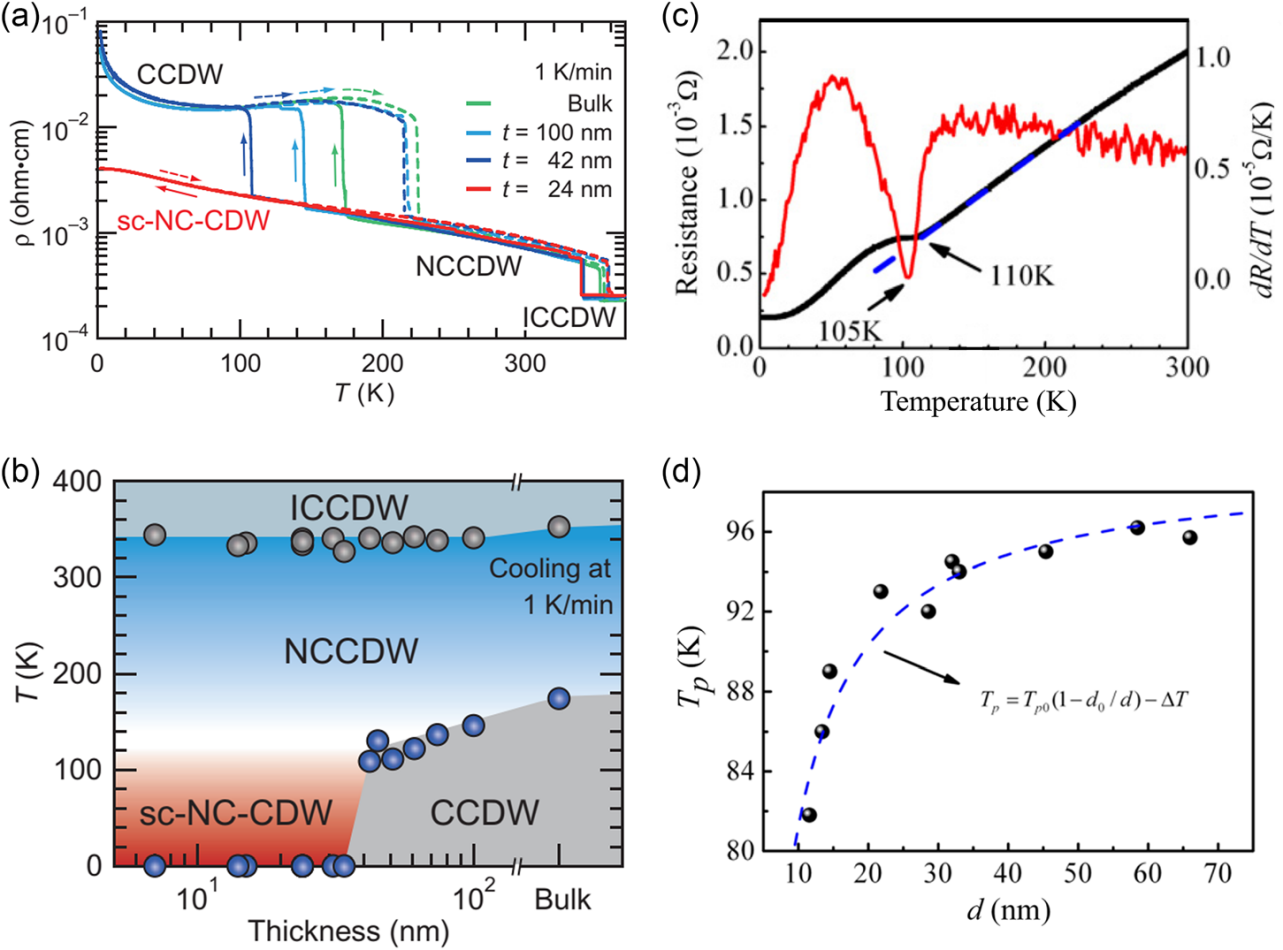
a) Temperature dependence of the resistivity for 1T‐TaS_2_ crystals with different thicknesses. The solid and broken lines represent the cooling and warming cycles, respectively.^[^ ^494^ ^]^ b) Temperature‐thickness phase diagram of 1T‐TaS_2_ nano‐thick crystals.^[^ ^494^ ^]^ a,b) Reproduced under the terms of the CC BY‐NC 4.0 license.^[^ ^494^ ^]^ Copyright 2015, The Authors. c) Temperature dependence of the resistance (black line: left axis) and its differential curve (red line: right axis) of the VSe_2_ single crystal.^[^ ^495^ ^]^ d) The CDW transition temperature as a function of thickness in VSe_2_ nanocrystals.^[^ ^495^ ^]^ c,d) Reproduced with permission.^[^ ^495^ ^]^ Copyright 2014, AIP Publishing.

Temperature‐dependent resistivity measurement is another convenient yet powerful method used for detecting the CDW phenomenon. Yoshida et al.^[^ ^494^ ^]^ observed the memristive phase switching behavior in 1T‐TaS_2_ nanosheets by investigating the temperature dependence of resistivity in 1T‐TaS_2_ considering different thicknesses during cooling and warming cycles (**Figure** **20**a). The authors reported that memristive resistivity switching exhibits a first‐order CDW phase transition, wherein kinetics become extremely slow with reduced thickness, resulting in the emergence of metastable states. Interestingly, the transition from NCCDW to CCDW, which is common in thicker crystals, completely disappears in crystals with a thickness of 24 nm. However, the transition from incommensurate CDW (ICCDW) to NCCDW persists in crystals with a thickness of up to 7 nm. The phase diagram (Figure 20b) indicates that in crystals thinner than 40 nm, the transition from NCCDW to CCDW is abruptly suppressed, stabilizing the supercooled NCCDW state. According to Yang et al.,^[^ ^495^ ^]^ the decrease in temperature in 1T‐VSe_2_ reduces the resistance until an inflection point is reached at 110 K, where an upturn kink becomes visible. The authors addressed the anomaly in the resistance to the onset temperature of the CDW transition (Figure 20c). The resistivity measurement indicated that the T_CDW_ decreases systematically from 105 K in bulk 1T‐VSe_2_ to 81.8 K in the 11.6‐nm‐thick nanosheet. (Figure 20d). The authors further suggested that electron concentration slightly increases with the decrease in thickness, possibly owing to the reduced CDW gap in VSe_2_. A similar tendency is observed in 1T‐TiSe_2_, where the critical temperature continuously increases from 200 to 240 K with a decrement in thickness.^[^ ^481^ ^]^ Ma et al.^[^ ^471^ ^]^ reported that few‐layer 1T‐VTe_2_ nanosheets exhibit an unexpected change in resistance at temperatures of 240 and 135 K, each of which corresponds to two different CDW phase transitions. The transition can be triggered by the temperature and in‐plane electric field because of local Joule heating, indicating that the coupling between electrons and phonons is an important factor impacting the formation of CDW.

**Figure 21:**
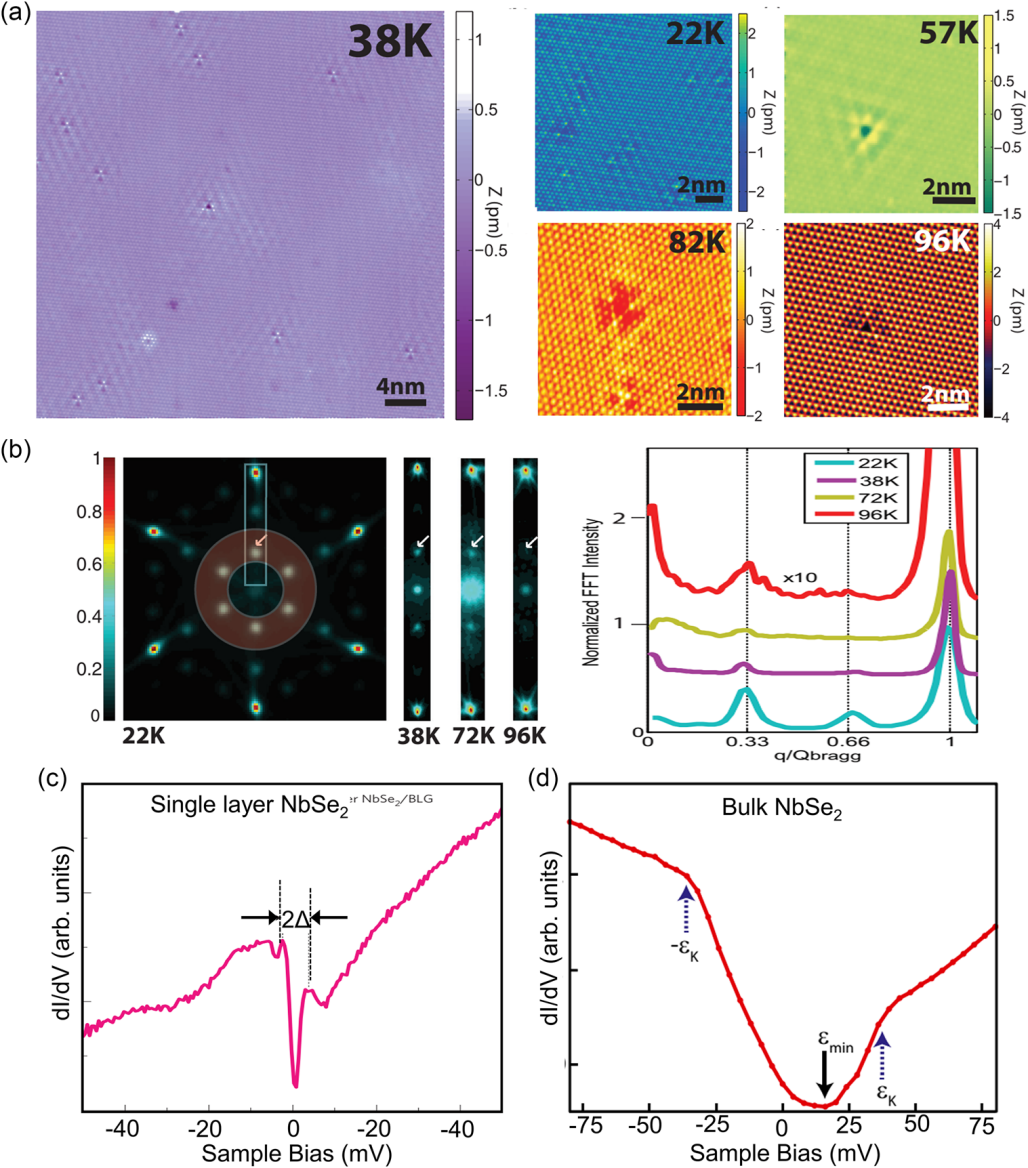
a) STM image of the NbSe_2_ surface at various temperatures below and above T_CDW_ = 33.7 K, where a thermal behavior of CDW is resolved.^[^ ^497^ ^]^ b) Two‐dimensional Fourier transform (2D‐FT) images and line profiles obtained at temperatures corresponding to (a).^[^ ^497^ ^]^ a,b) Reproduced with permission.^[^ ^497^ ^]^ Copyright 2014, American Physical Society. CDW gap of c) single‐layer and^[^ ^487^ ^]^ d) bulk NbSe_2_.^[^ ^496^ ^]^ c) Reproduced under the terms of the CC BY license.^[^ ^487^ ^]^ Copyright 2015, Springer Nature Limited. d) Reproduced under the terms of the CC BY license.^[^ ^496^ ^]^ Copyright 2013, The Authors, published by PNAS.

Temperature‐variable low‐temperature STM and STS are particularly advantageous for directly visualizing the atomic structure and CDW gaps of TMDs in real space. This can determine the wavelength of the charge modulation along with the electronic transition temperature.^[^ ^496^ ^]^ Arguello et al.^[^ ^497^ ^]^ directly observed the CDW transition of 2H‐NbSe_2_ at atomic resolution using STM analysis. When the sample was cooled, the authors initially observed a short‐range CDW with a 3 × 3 atom periodicity that was initiated adjacent to defects at temperatures several times higher than the bulk transition temperature (**Figure** **21**a). The short‐range CDW can be observed using STM up to temperatures of approximately 3T_CDW_, with a gradual decrease in the range and coverage of the CDW with the increasing temperature. The corresponding FFT images reveal that the wavelength of the CDW remains constant despite the temperature variations. However, the intensity of the CDW peak progressively weakens with the increasing temperature and becomes negligible at ≈100 K (Figure 21b). Furthermore, Ugeda et al.^[^ ^487^ ^]^ reported a narrow CDW gap of 4 meV in single‐layer NbSe_2_ at 5 K using STS analysis (Figure 21c). Considering that the CDW gap in bulk 2H‐NbSe_2_ is nearly 70 meV wide (Figure 21d),^[^ ^496^ ^]^ the small‐sized gap in the single layer is particularly unexpected and may be attributed to the removal of bands beyond the Fermi level.

## 7 Applications

Thus far, we have discussed the importance of diverse polymorphic phases of TMDs in inducing their unique properties. In this section, we discuss the range of practical applications considering various phase transformation techniques and interfacial mechanisms that can be used for integrating polymorphic vdW materials to develop high‐performance functional devices.

### 7.1 Electronic Devices

#### 7.1.1 FETs

In contrast to graphene, several vdW TMD nanosheets possess sizable bandgaps of approximately 1–2 eV, paving the way for the development of a new type of FET. Therefore, future TMD‐based FETs should have a high on/off ratio, carrier mobility, and conductivity to enhance their performance. These properties are expected to be derived from the unique features of TMDs, such as the low effective mass, various *d*‐electron effects, and layer‐number‐dependent band structures.^[^ ^50^ ^]^ For instance, FETs fabricated from an uncovered single‐layer MoS_2_ are known to exhibit mobilities ranging from 0.2 to 12 cm^2^ V^−1^ s^−1^, which can be significantly degraded by temperature because of the thermally activated hopping transport.^[^ ^498^, ^499^, ^500^ ^]^ In comparison with these, FETs fabricated from few‐layer MoS_2_ or encapsulated single‐layer MoS_2_ exhibit substantially higher mobilities (60–500 cm^2^ V^−1^ s^−1^)^[^ ^501^, ^502^ ^]^ with band‐like transport. This indicates that the charge transport mechanism strongly depends on extrinsic scattering sources, such as interfacial resistances, adsorbates, and the effects of substrates. To relieve the issue and improve the performance of FET, researchers have attempted to reduce the contact resistance through phase engineering.^[^ ^503^ ^]^ Particularly, since the 1T and 1T’ phases of MoX_2_, WX_2_, group‐V MX_2_ exhibit metallic characteristics, they have been regarded as suitable contact electrode candidates in spite of the Fermi‐level pinning and disordering issues.^[^ ^503^, ^504^ ^]^ Furthermore, it was reported that metallic vdWMs can suppress metal‐induced gap states (MIGS) formed on semiconductors, weakening the Fermi‐energy level pinning effect and the Schottky barrier on the contact surface.^[^ ^503^ ^]^

**Figure 22:**
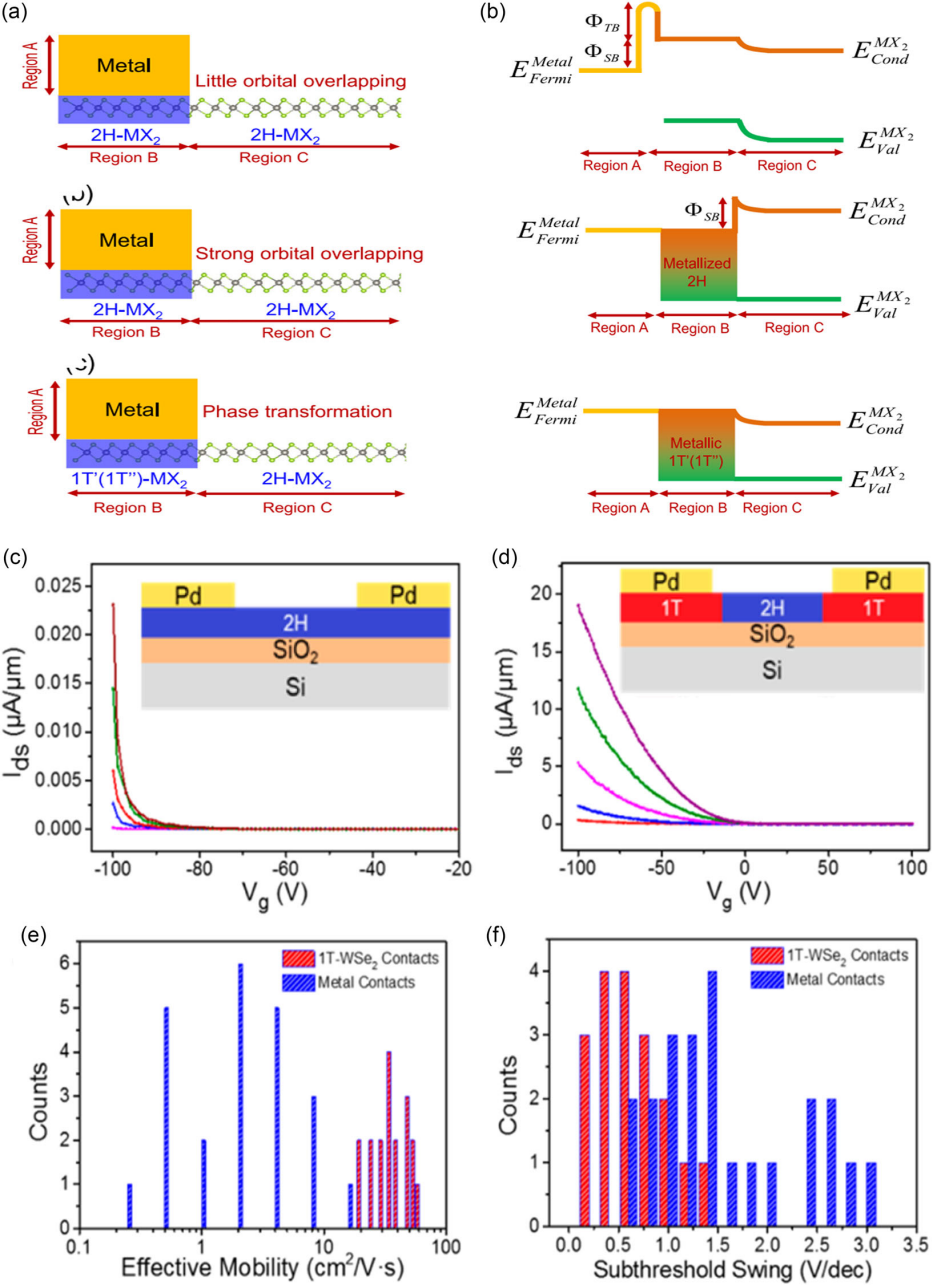
a) Illustration of three types of contact between TMX_2_ and metal electrode.^[^ ^505^ ^]^ b) Their corresponding band structure depending on the degree of orbital overlapping. The tunnel and Schottky barriers are denoted as Φ_TB_ and Φ_SB_, respectively.^[^ ^505^ ^]^ a,b) Reproduced under the terms of the CC BY license.^[^ ^505^ ^]^ Copyright 2018, The Authors, published by Springer Nature. *I*_ds_–*V*_g_ curves for back‐gated 2H‐WSe_2_ FET device with c) direct metal contacts and^[^ ^81^ ^]^ d)1T‐WSe_2_ contact. Inset: Schematic of the back‐gated WSe_2_ FET with metal contacts. The SEM image in (d) indicates the 2H‐WSe_2_ sample with 1T phase in the middle region.^[^ ^81^ ^]^ Statistics of e) effective mobility and f) subthreshold swing (SS) of 2H‐WSe_2_ FETs with metal contacts and 1T‐WSe_2_ contacts.^[^ ^81^ ^]^ c–f) Reproduced with permission.^[^ ^81^ ^]^ Copyright 2015, American Chemical Society.

These variations suggest three possible types of contact between vdW materials and the electrode in terms of the degree of interfacial orbital overlap and charge transfer (**Figure** **22**a,b).^[^ ^505^ ^]^ The first type of contact is observed when a significant lattice mismatch exists between the vdW material and electrode, wherein both the tunnel and Schottky barriers act as contact resistances, impeding the electronic transport from the metal to the TMX_2_ channel. Second, if the lattice coherency and orbital overlap increase to a certain point where the tunneling barrier reduces to a negligible level, the Schottky barrier would contribute to the overall contact resistance. In this case, the optimal contact resistance is determined by the lowest Schottky barrier height regardless of whether it corresponds to electrons or holes.

Third, as phase engineering converts the 2H‐semiconducting type to the 1T‐metallic type, direct electron injection without Schottky barriers can be achieved. This is because the Fermi level of a metallic TMX_2_ is higher than the conduction band edge (CBE) of a semiconducting TMX_2_, which suppresses the formation of a Schottky barrier.

To circumvent this issue, Das et al.^[^ ^506^ ^]^ initially studied the effect of the Schottky barrier on the carrier transport in MoS_2_‐based FETs by varying the contact metal. The authors suggested that the Schottky barrier can be reduced by matching the low work function of metals with CBE of 2H/1H‐MoS_2_, improving the transport behavior. In terms of phase engineering, Kappera et al.^[^ ^507^ ^]^ exploited the e‐beam lithography patterning to partially convert 2H‐MoS_2_ to 1T‐MoS_2_, forming an ohmic homojunction. They synthesized metallic 1T‐MoS_2_ through the organolithium chemical method, which was maintained even after organolithium was removed. They further demonstrated that the resistance value in the metallic 1T phase can be reduced to as low as 200 Ω μm at zero gate bias, resulting in high drive currents, high mobility, low subthreshold swing (SS) values, and high on/off ratios. Ma et al.^[^ ^81^ ^]^ reported that the phase of single‐layer WSe_2_ can be reversibly switchable from the semiconducting 1H phase to metallic 1T phase by repeating the selective n‐BuLi treatment and thermal annealing (Figure 22c–f). The authors suggested that the metallic 1T‐WSe_2_ can suitably operate as an electrode for 2H‐WSe_2_‐based FETs owing to their high mobilities (up to 66 cm^2^ Vs^−1^), high on/off ratios (up to 10^7^), and better SS values (0.658 V dec^−1^) than devices with metal contacts, such as Pd/Ti and Au/Ti. Cho et al.^[^ ^457^ ^]^ reported a laser‐induced phase transformation in MoTe_2_ and fabricated a MoTe_2_‐based transistor with an ohmic heterophase homojunction that exhibited an increase of nearly 50‐fold in mobility compared with the direct metal‐2H‐MoS_2_ junction, as illustrated in Figure 17a. Furthermore, although T_d_‐WTe_2_ exhibits high mobility ranging from 200 to 2000 cm^2^ V^−1^ s^−1^,^[^ ^508^ ^]^ its semi‐metallic property hinders its application in FET devices. To address this issue, doped or alloyed TMDs have been suggested as alternatives to widen the range of available phase engineering options. Wang et al.^[^ ^332^ ^]^ reported that the phase transition of WS_2(1−*x*)_Te_2*x*_ is converted from T_d_ to 2H at a threshold concentration of *x* = 0.5, resulting in an opening of the bandgap. In the 2H phase, the gap size of the material is tunable in the range of 1.67 to 1.97 eV before closing upon transitioning to the 1T′ phase. Therefore, in the 2H‐semiconductor regime, WS_2(1−*x*)_Te_2*x*_ exhibits *p*‐type mobility and on/off ratio of 7.4 cm^2 ^V^−1^ s^−1^ and 10^7^ for *x* = 0; and 4.4 cm^2 ^V^−1^ s^−1^ and 10^7^ for *x* = 0.2, respectively. Guo et al.^[^ ^509^ ^]^ demonstrated that with the increase in the percentage of Se doping, ambipolar behavior in MoSe_2*x*_Te_2(1–*x*)_ changes to n‐type behavior. This can be attributed to the increase in the bandgap caused by the Schottky‐barrier‐governed electron transport. Additionally, the increase in S content in MoS_2*x*_Te_2(1–*x*)_ changes the conduction behavior from *p*‐type to n‐type, increasing the on/off ratio owing to the large bandgap.^[^ ^510^ ^]^

**Figure 23:**
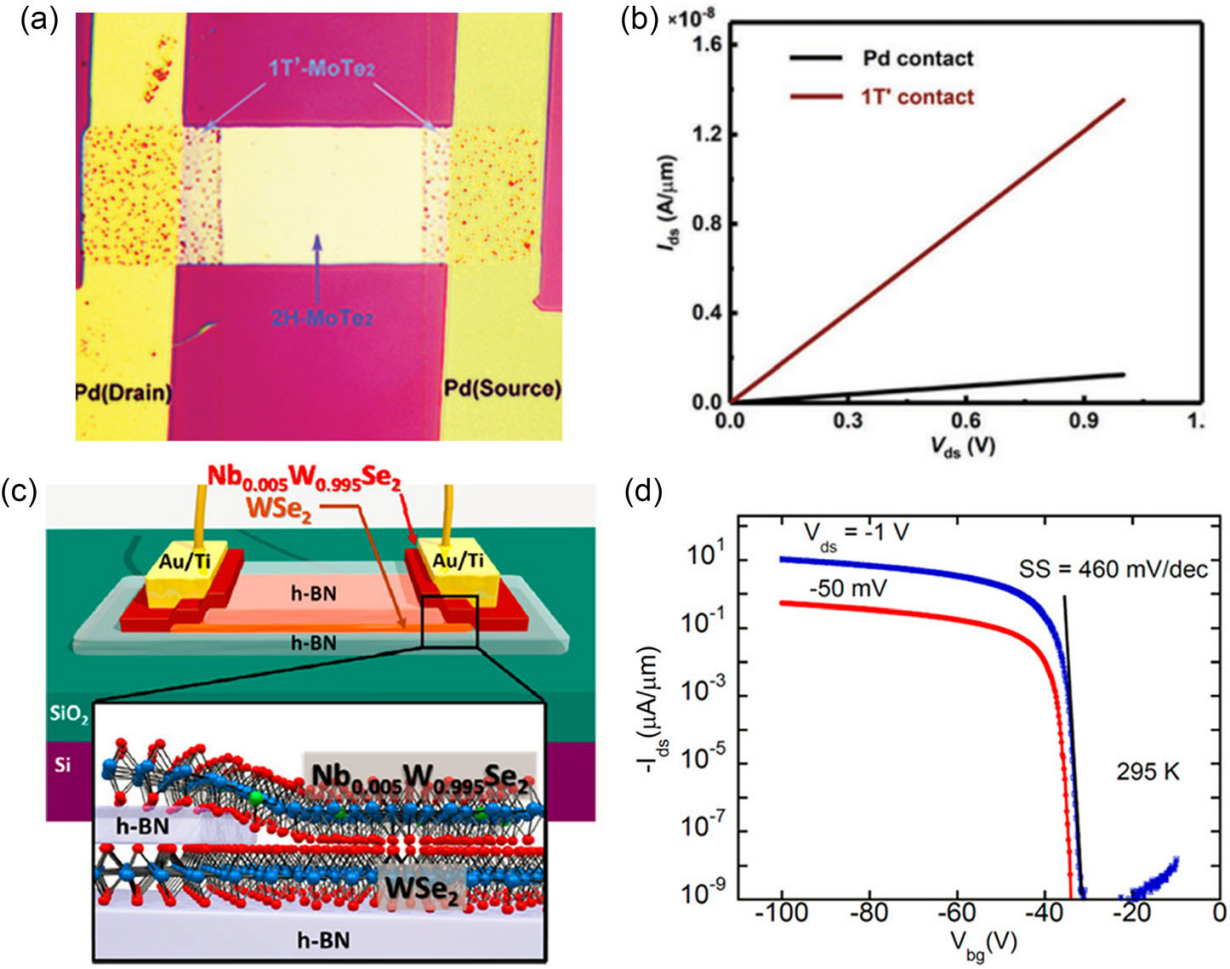
a) Structure of FET sensors based on in‐plane 1T′/2H/1T′ MoTe_2_.^[^ ^511^ ^]^ b) Output curves of the devices with 1T′/2H/1T′ and the 2H only contact at *V*_gs_ = 0 V and room temperature.^[^ ^511^ ^]^ a,b) Reproduced with permission.^[^ ^511^ ^]^ Copyright 2022, Wiley‐VCH GmbH. c) Schematics of WSe_2_‐based FET with 2D/2D contacts.^[^ ^516^ ^]^ d) At room temperature, the back‐gate voltage modulation (*V*_bg_) in a WSe_2_ field‐effect transistor (FET) with a channel length of 10.8 μm and width of 3.0 μm results in an outstanding on/off ratio of up to 10^9^ for the source‐drain current (*I*_ds_).^[^ ^516^ ^]^ c,d) Reproduced with permission.^[^ ^303^ ^]^ Copyright 2016, American Chemical Society.

In addition, Zhang et al.^[^ ^511^ ^]^ solved the problem of high contact resistance by fabricating 1T′/2H polymorph through metallic 1T′‐MoTe_2_ contact in MoTe_2_ based FET sensor.(**Figure** **23**a) Ohmic contact (with contact resistance ≈7.1 kΩ μm) was formed due to the seamless connection in‐plane heterophase. Figure 23b shows the output curve in the room temperature, both of which show linear behavior, but 11 times higher *I*_ds_ of 1T′/2H/1T′‐MoTe_2_. In addition, the 1T′/2H/1T′ FET is a sensor that detects NH_3_, and it also showed a high output current of micro level and high sensitivity that detects NH_3_ below 5 ppm. In particular, it is significant in that a phase engineering method of growing 2H/1T′ by patterning Mo film, making the exposed region into MoO_3_ through oxygen plasma treatment, and then tellurization.

However, group‐10 TMDs, namely, PtS_2_, PtSe_2_, and PdSe_2_, have been theoretically estimated to exhibit mobilities as high as approximately 200 cm^2 ^V^−1^ s^−1^, which is larger than those of most other TMDs; they also exhibit relatively high stability when exposed to air.^[^ ^114^, ^512^, ^513^ ^]^ In comparison with other TMDs comprising partially occupied *d*‐orbitals, those of group‐10 TMDs are almost fully occupied, and the *p*_*z*_ orbitals are strongly hybridized. Consequently, the interlayer interaction can be more enhanced than that observed in other TMDs. Owing to their strong interlayer interaction, certain group‐10 TMDs exhibit widely variable bandgaps and even metal–semiconductor transitions,^[^ ^114^, ^512^, ^513^, ^514^, ^515^ ^]^ indicating their potential to be used in single material‐based devices with dual operation of FETs and high electrical conductivity. Ciarrocchi et al.^[^ ^514^ ^]^ revealed that PtSe_2_ with a thickness of ≈13 nm exhibits metallic behavior with a contact resistance as low as 70 Ω μm. However, when the thickness is lesser than approximately 2.5 nm, PtSe_2_ exhibits semiconducting behavior with ambipolar transport characteristics, a bandgap of less than ≈2.2 eV, and an on/off ratio of ≈10^5^. The SS values were measured to be 83 and 106 mV dec^−1^ for holes and electrons, respectively. Furthermore, the authors suggested that the mobility of the material can increase up to approximately 1890 cm^2 ^V^−1^ s^−1^ by implementing ohmic contact and surface encapsulation. Oyedele et al.^[^ ^114^ ^]^ reported that the few‐layer orthorhombic PdSe_2_‐FET exhibits a thickness‐dependent metal–insulator transition as well as a high mobility of ≈158 cm^2 ^V^−1^ s^−1^. Additionally, unlike other typical TMDs, PdSe_2_ and PdS_2_ exhibit different effective masses with anisotropic in‐plane transport owing to their unique puckered pentagonal structures.^[^ ^108^, ^247^ ^]^ The puckered PdSe_2_ enables another polymorphic phase transition between orthorhombic PdSe_2_ (O‐PdSe_2_) and monoclinic PdSe_2_ (M‐PdSe_2_). Gu et al.^[^ ^134^ ^]^ successfully synthesized two different types of PdSe_2_s by controlling the Se‐supply conditions. The reported electrical transport measurements indicate that the M‐PdSe_2_‐FET exhibits n‐type charge carrier conduction, with electron mobilities reaching up to ≈298 cm^2 ^V^−1^ s^−1^. Additionally, the device exhibits a strong in‐plane anisotropic mobility of ≈1.9. Remarkably, not only is this value higher than that of the typical O‐PdSe_2_ (≈1.1),^[^ ^114^ ^]^ but it is also higher than that of most anisotropic 2D materials.

Another solution to reduce contact resistance is to form a 2D/2D ohmic contact by controlling a wide range of work functions and bandgaps using phase engineering. The interaction at the interface of the metal–semiconductor junction affects the Fermi‐energy pinning effect and carrier tunneling efficiency. In particular, it inhibits the formation of MIGS through vdW contacts, which can control the Schottky barrier via back gate voltage and weaken Fermi‐energy level pinning.^[^ ^504^ ^]^ Li et al.^[^ ^504^ ^]^ presented NiTe_2_/ZrSe_2_, NiTe_2_/PdSe_2_, HfTe_2_/PdTe_2_, and TaSe_2_/MoTe_2_ as TMDs with a high probability of ohmic contact, weak Fermi‐energy level pinning, and high carrier tunneling through DFT calculations. Using thermodynamic calculations, each was divided into a p‐type ohmic contact, an n‐type ohmic contact, and a Schottky contact. Researchers not only provided a basic understanding of contact characteristics in vdW contacts but also presented the direction of researches from the perspective of device applications. Experimentally, Chuang et al.^[^ ^516^ ^]^ reduced contact resistance by fabricating an FET with 2D/2D ohmic contact via phase engineering through doping in the source and drain regions of channel WSe_2_. (Figure 23c) Contact resistance was reduced to 0.3 kΩ, and the on‐off ratio also increased to more than 10^9^(Figure 23d). Conventional doping methods give damage to nanosheets or have weaknesses in long‐term stability. However, they succeeded in ohmic contact with air and thermal stability using the substitutional doping method that forms an Nb‐MoS_2_ covalent bond during synthesis. They formed phase‐engineered degenerately *p*‐doped WSe_2_ (Nb_0.005_W_0.995_Se_2_) 2D drain/source electrodes using dry transfer. Recently, research has been conducted to create 2D/2D contacts using phase‐engineered WSe_2_ doped with Nb.^[^ ^517^ ^]^ Therefore, even in 2D/2D vdW contact, phase engineering can be an important step toward a device with better performance, opening the path to fundamental quantum phase transitions in contact engineering using TMDs.

#### 7.1.2 Diode

**Figure 24:**
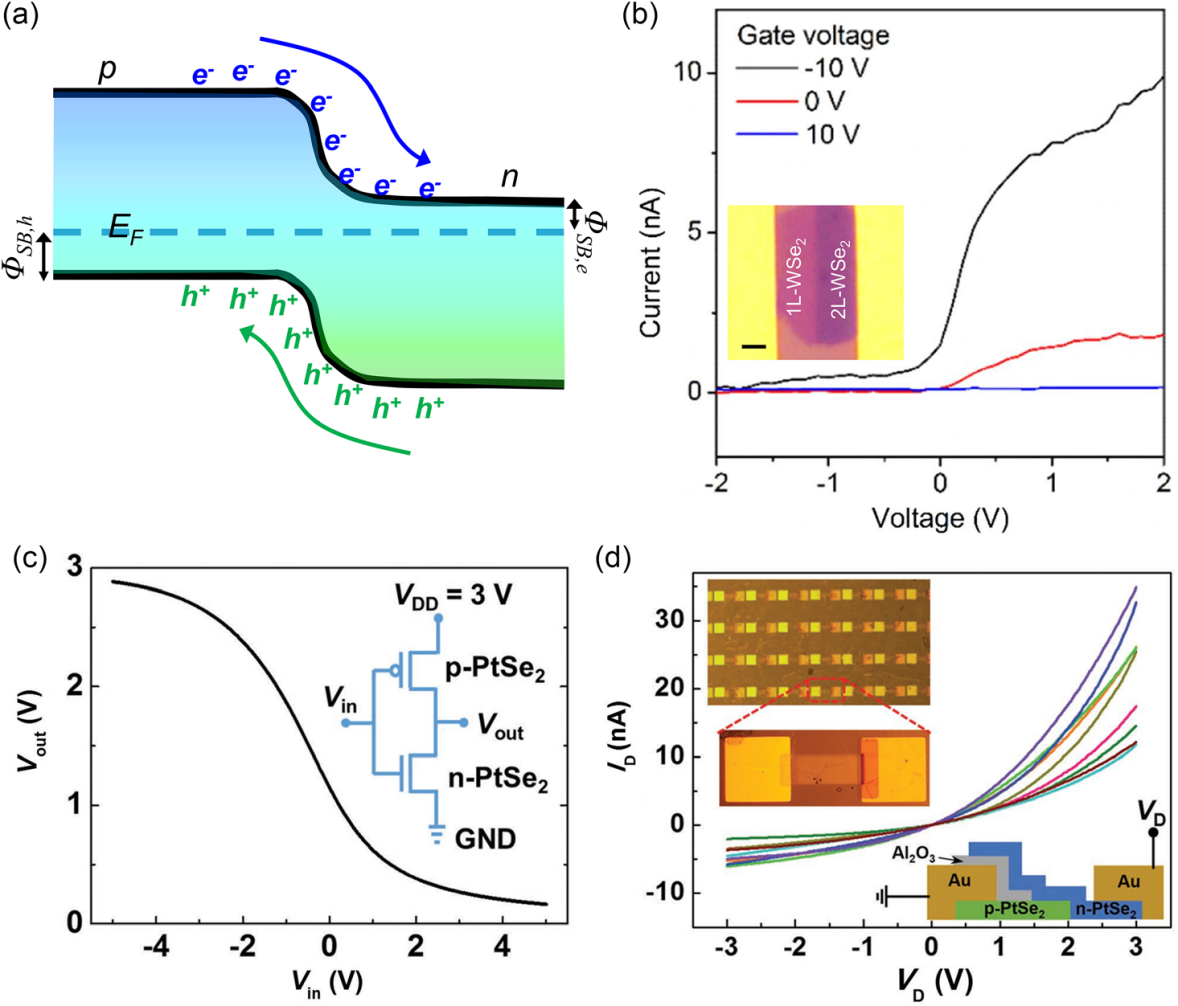
a) Schematic of the energy band diagram of the 1L–2L WSe_2_ heterojunction states. b) Current–voltage (*I–V*) curves of the 1L–2L WSe_2_ device exhibiting the rectifying behavior at −10 V (black), 0 V (red), and 10 V (blue).^[^ ^518^ ^]^ Reproduced with permission.^[^ ^518^ ^]^ Copyright 2016, IOP Publishing. c) Voltage transfer curve of a PtSe_2_ complementary inverter circuit. Inset: Schematic of the logic inverter.^[^ ^519^ ^]^ d) Rectifying behavior of the PtSe_2_ n–p diode. Insets: The optical image of a *p–n* diode array with a schematic of the device cross‐section.^[^ ^519^ ^]^ c,d) Reproduced with permission.^[^ ^519^ ^]^ Copyright 2018, Wiley‐VCH.

In an ideal band diagram of the p–n junction at the equilibrium state, an internal field in the depletion region propels electrons and holes in opposite directions (**Figure** **24**a). Specifically, as the barrier height (Φ_SB–h_) between VBE and E_F_ on the *p*‐side is considerably lower than that (Φ_SB–e_) between CBE and E_F_ on the *n*‐side, the carrier transport efficiency is higher for a positive bias than that for a negative one. This ultimately results in rectifying behavior. A homo‐*p–n* junction diode is based on a single 2D material, where the bandgap on each side is modified by the thickness‐dependent quantum confinement. Xu et al.^[^ ^518^ ^]^ created a lateral junction diode between single‐layer and double‐layer nanosheets of 2H‐WSe_2_ by selectively removing the top layer of WSe_2_ through photolithography and Ar plasma etching (Figure 24b). The authors employed KPFM to measure the voltage differences of approximately 75 and −302 mV at the drain/single‐layer WSe_2_ and source/bilayer WSe_2_ interfaces, causing n‐type and *p*‐type doping of the single‐layer and bilayer WSe_2_, respectively, with respect to the Au electrodes. Furthermore, a voltage difference of ≈79 mV is detected at the interface between the single‐layer and bilayer materials, indicating the presence of a built‐in potential that enhances the separation of excitons with higher efficiency.

Xu et al.^[^ ^519^ ^]^ suggested a facile and controllable method for synthesizing few‐layer PtSe_2_ films with an effective doping level at the wafer scale. The authors obtained continuous PtSe_2_ films with a few‐layer thickness by selenizing PtCl_4_ precursors in a CVD furnace (Figure 24c,d). Moreover, the doping level was determined to be controlled through the cooling process, which was correlated to the extent of selenization. If a sample is gradually cooled down with a continuous supply of Se even after the selenization process, the resulting PtSe_2_ becomes p‐type owing to excess Se atoms. Conversely, if the sample is rapidly cooled down by removing the Se precursor after selenization, the resulting PtSe_2_ becomes n‐type, approaching the stoichiometric ratio. Therefore, reasonably high mobilities of approximately 14 and 15 cm^2 ^V^−1^ s^−1^ were obtained for both types of doping in the p‐type and n‐type PtSe_2_, with a nearly symmetric p‐type and n‐type performance, respectively. Furthermore, the authors fabricated a complementary metal–oxide‐semiconductor (CMOS) inverter and a vertically stacked p–n junction based on bilayer PtSe_2_ by transferring and stacking two different types of PtSe_2_ samples. Here, the drain well exhibited typical rectifying behavior owing to the p–n junction, with a relatively modest current on/off ratio of ≈100 in the *I–V* characteristics.

Ahn et al.^[^ ^255^ ^]^ selectively synthesized 2D tin sulfide crystals of either hexagonal SnS_2_ or orthorhombic SnS by controlling the amount of H_2_ gas inserted during the growth process. They reported that the gate‐bias‐dependent rectifying behavior occurs in the back‐gated 2D SnS_2_ − SnS vertical heterojunction device. The output current in the diode is largely governed by the higher resistivity of the n‐type SnS_2_ rather than the p‐type SnS in the p–n series, resulting in an increase in current with the gate bias. Furthermore, unlike conventional p–n diodes, a depletion region cannot be defined between the p–n layers in a 2D diode junction owing to the limited diffusion. Therefore, the boundary region can be examined based on the interlayer recombination processes between two majority carriers crossing the abrupt potential discontinuity, such as the Langevin or Shockley–Read–Hall recombination, which is mediated by the interlayer defect states.^[^ ^520^ ^]^ Kim et al.^[^ ^521^ ^]^ reported that SnS_2_ nanosheets can be transformed to SnS by removing sulfur atoms using Ar plasma treatment. Here, SnS_2_ and SnS are n‐type and *p*‐type semiconductors with approximate bandgaps of 2.2 and 1.2 eV, respectively. Therefore, the junction of SnS_2_ and SnS can form a staggered band alignment with type‐II heterojunctions, resulting in a typical rectifying diode behavior. Using the Shockley diode equation, the authors calculated the ideality factor of the device to be ≈3.2. This value is significantly greater than the typical range of values (between 1 and 2) observed in conventional diodes.^[^ ^522^ ^]^ The high ideality factor can be attributed to the significant effect of the defect in the vdW heterojunction.^[^ ^523^, ^524^ ^]^ Furthermore, Gong et al.^[^ ^525^ ^]^ used intercalation to separately achieve p‐type, n‐type, and degenerate doping features from a single parent vdW material. Unlike n‐type SnS_2_, which results from the S vacancy generated during the CVD process, Cu‐intercalated bilayer SnS_2_ presents hole‐type field‐effect mobility of ≈40 cm^2^ V^−1^ s^−1^, whereas Co‐SnS_2_ exhibits metallic behavior. Combining these results, the authors fabricated a seamlessly integrated p–n junction with metallic contact only in 2D materials (SnS_2_ (n‐type), Cu‐SnS_2_ (*p*‐type), and Co‐SnS_2_ (metal)), which is generally challenging via typical transfer methods.

#### 7.1.3 Resistive Random‐Access Memory (RRAM)

**Figure 25:**
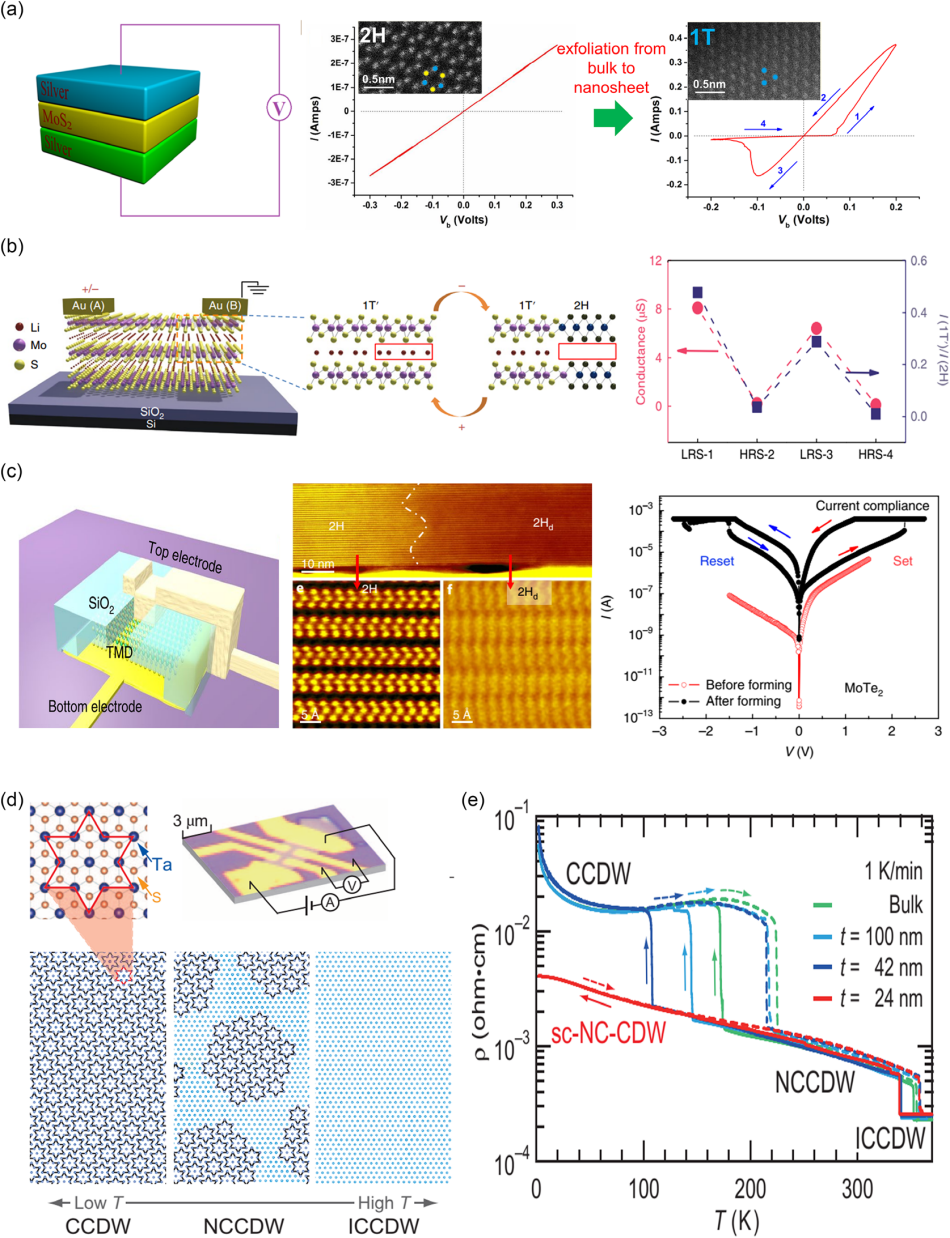
a) Schematic of the Ag/MoS_2_/Ag device structure (left). Typical current–voltage (*I–V*) characteristic curve for the bulk 2H‐MoS_2_ (center) and exfoliated 1T‐MoS_2_ nanosheet (right).^[^ ^519^ ^]^ Reproduced with permission.^[^ ^519^ ^]^ Copyright 2016, American Chemical Society. b) Schematic of the local 2H–1T′ phase shift in MoS_2_ by controlling the migration concentration of intercalated Li^+^ ions based on a lateral electric field (left). Correlation between changes in device conductance and the Raman shift between the 1T′ and 2H phases during resistive switching.^[^ ^528^ ^]^ Reproduced under the terms of the CC BY license.^[^ ^528^ ^]^ Copyright 2018, The Authors, under exclusive licence to Springer Nature Limited. c) Schematic of a vertical TMD device (left). HAADF‐STEM image indicating the coexistence of a distorted structure (2H_d_) with 2H in Mo_0.96_W_0.04_Te_2_ (center).^[^ ^529^ ^]^ Reproduced under the terms of the CC BY license.^[^ ^529^ ^]^ Copyright 2018, The Authors, published by Springer Nature. The hysteresis current becomes clear after the forming process (right). d) Optical microscope image of a nano‐thick crystal device (top‐right). Schematic of CCDW (left), NCCDW (middle), and ICCDW (right) phases (bottom). The dark blue David‐star circles represent the Ta atoms clustered for the CDW formation.^[^ ^494^ ^]^ e) Temperature dependence of the resistivity for bulk and nano‐thick 1T‐TaS_2_.^[^ ^494^ ^]^ d,e) Adapted from the Authors, published by Science Advances.

Integrated electronic systems cannot be fabricated without memory devices as they are considered a fundamental and necessary element. Therefore, a controllable phase change has been extensively investigated in TMDs for the feasibility of a memristive and neuromorphic application. Cheng et al.^[^ ^526^ ^]^ prepared 1T‐MoS_2_ using the ion intercalation method, where electron injection triggers the phase transition from 2H to 1T. Subsequently, they fabricated a symmetric vertical heterostructure by coating both sides of MoS_2_ nanosheets with conductive Ag paste in both the 2H and 1T phases. The device is similar to vertically configured metal–insulator–metal (MIM) structures, where the resistive switching behavior is governed by the phase transition of MoS_2_ nanosheets (**Figure** **25**a). The authors further demonstrated that bulk 2H‐MoS_2_ displays ohmic behavior, whereas exfoliated 1T‐MoS_2_ nanosheets exhibit a characteristic bipolar resistive switching effect when an electric field is applied between the two Ag electrodes. The application of a strong external electric field to the system can induce the displacement of Mo and S ions in the 1T phase, leading to lattice distortions. These distortions can delocalize electrons, resulting in increased conductivity. Similarly, Zhang et al.^[^ ^527^ ^]^ fabricated flexible nonvolatile memory devices based on MoS_2_‐polyvinylpyrrolidone (PVP) nanocomposite, where the 2H/1T phase of MoS_2_ was adjusted using a two‐step hydrothermal method. They fabricated the nanocomposite memory by mixing 2H‐ and 1T‐MoS_2_, which was featured with low set/reset voltages, large on/off ratio, long retention time, adequate endurance, and excellent mechanical flexibility. Furthermore, Zu et al.^[^ ^528^ ^]^ demonstrated that the 2H‐1T′ memristive effect can be achieved by driving the migration of Li^+^ ions with a lateral electric field in a Li‐intercalated MoS_2_‐based device (Figure 25b). The authors reported that the proposed method can enable a better reversible memory behavior compared with that of conventional memristive devices based on defect doping or conductive filament formation. This is because the redistribution of Li ions at concentrations higher than the critical level^[^ ^370^ ^]^ is driven by an electric field.

In general, phase shifting induced by the motion of ions exhibits a relatively slow response to external electric fields owing to its sluggish nucleation and percolative growth; this limits its application in electric devices requiring fast switching speeds. Apart from the ion‐regulated phase transition process, Zhang et al.^[^ ^529^ ^]^ fabricated a vertical memory device‐based on 2H‐MoTe_2_ and Mo_1–*x*_W_*x*_Te_2_, where the external electric field induced the phase transition and changed the semiconducting 2H phase to either a less resistive 2H_d_‐transient structure or a metallic T_d_ phase (Figure 25c). The temperature‐dependent resistance curve indicates that the 2H_d_ state can pivot from a semiconducting to a metallic state depending on the transient state. Here, the voltage set in Mo_1−*x*_W_*x*_Te_2_ is smaller than that in MoTe_2_, and the value decreases further with a decrease in thickness. Additionally, the authors verified that reproducible resistive switching can be achieved within 10 ns between high‐ and low‐resistance states, and the on/off current ratio can be equivalent to 10^6^ in selector‐less memory architecture. The results indicate the potential of MoTe_2_ and WTe_2_ to be used in nonvolatile memory applications.

As CDW‐based memory devices exhibit substantially faster switching responses than either ion‐ or field‐induced memristive devices, they have received significant attention from researchers. Stojchevska et al.^[^ ^530^ ^]^ reported that the ordering state in vdW materials featured as CDW (1T‐TaS_2_) can be switched using 35‐fs pulses, which is comparable to the speed record of 40‐fs current pulses for magnetic materials. Furthermore, Vaskivskyi et al.^[^ ^531^ ^]^ revealed the potential of 1T‐TaS_2_ nanosheet in the ultrafast nonvolatile memory area. A current pulse traveling through 1T‐TaS_2_ converts a commensurately ordered polaronic Mott insulating state into a metastable electronic state with textured domain walls. This is accompanied by the transfer of polarons to band states and simultaneous switching from an insulator to a metal. The authors demonstrated that CDW‐based nonvolatile memory devices can achieve a large resistance change with a switching speed of 30 ps and ultralow energy consumption per bit by manipulating all electronic states. Particularly, 1T‐TaS_2_ exhibits a sequential CDW transition from the ICCDW (temperature > 350 K) to the CCDW state (temperature < 180 K) via the NCCDW state (180 K < temperature < 350 K) during the cooling process; however, it remains in the triclinic state at 220 K < T < 280 K during the warming process. Yoshida et al.^[^ ^494^ ^]^ (Figure 25d,e) reported that few‐layer 1T‐TaS_2_ nanosheets undergo two phase transitions; the first transition occurs from the ICCDW to NCCDW state at 350 K, whereas the second is a transition to the Mott state of CCDW between 100 and 220 K. The direction of the temperature sweeps also affects the behavior of the transition. Additionally, the authors demonstrated that the kinetics of first‐order phase transitions in a nanosheet can be systematically adjusted by varying the thickness of 1T‐TaS_2_, resulting in the emergence of a metastable metallic state when an in‐plane voltage is applied. The CDW phase transitions produce both hysteretic current–temperature and memristive *I–V* curves, rendering them promising candidates for memory applications.

### 7.2 Electrochemistry

In the electrochemical field, studies on controlling the phase of TMDs are being actively conducted with respect to batteries, supercapacitors, and electrocatalysts. Controlling the phases of TMDs can facilitate the modifications in structural changes and chemical and physical properties.

#### 7.2.1 Energy Storage

The importance of energy storage has increased owing to environmental pollution, leading to the emergence of sustainable eco‐friendly energy sources and electric vehicles. Both batteries and supercapacitors have been steadily investigated to enhance the charge transfer properties required for the chemical reaction and storage of electrical energy. Furthermore, various attempts have been made to stabilize the 1T phase in energy devices. In this section, we introduce the research on batteries and supercapacitors based on the phase control of TMDs.

##### Battery

The primary factors impacting the research on batteries can be divided into two categories, namely, conductivity and high surface area. Based on these factors, phase engineering has been explored with respect to the conductive 1T phase of MoS_2_. Li ions are known for their high diffusivity and their ability to individually interact with electrodes favorably. Xu et al.^[^ ^532^ ^]^ calculated the adsorption and diffusion energy of Li ions in single‐layer 1T‐MoS_2_ using DFT analysis. The binding energy of Li decreases as the concentration of Li ions increases. Particularly, when the Li ions are twice as many as those of Mo, the binding energy barrier with MoS_2_ is higher than that of the Li metal. In the case of diffusion energy, the diffusion barrier of 1T‐MoS_2_ is higher than that observed for 1H‐MoS_2_; however, it is comparable to that of LiFePO_4_ used in industrial applications. Therefore, considering the better conductivity in the 1T phase than that observed in the 1H phase, researchers have suggested the possibility of using 1T‐MoS_2_ as a battery storage device.

Researchers have widely explored the concept of combining 1T phase TMDs with metallic materials. Jiao et al.^[^ ^156^ ^]^ fabricated a battery that combines metallic 1T‐MoS_2_ with a unique porous structure using the solvothermal method, which enabled the mass production of 1T‐MoS_2_ with adequate intrinsic conductivity and hydrophilic surfaces. Restacking is an issue commonly observed in all 2D materials in liquid, and this was noticed in 1T‐MoS_2_ as well; herein, the position where the Li ions could enter was reduced when the TMD was stacked. The researchers solved this issue by introducing a porous nanotube template. The structure of this porous nanotube facilitates the uniform and rapid diffusion of lithium ions and ensures vertical alignment of 1T‐MoS_2_ with the nanotube, which prevents the lithium ions from being restored. Therefore, the stability in the proposed structure was 1100 mAh g^−1^ at a current density of 5 A g^−1^ even after 350 cycles. The study^[^ ^156^ ^]^ structurally supplemented the limitations of 1T‐MoS_2_ and devised a manufacturing method to pave the way for using 1T‐MoS_2_ as an anode material for batteries. Similar to previous researchers, Lu et al.^[^ ^533^ ^]^ confirmed enhanced battery performance using vertically aligned 1T‐MoS_2_ with the high reversible capacity of 1269 mAh/g at a current density of 100 mA g^−1^ after 100 cycles. And Xiang et al.^[^ ^159^ ^]^ fabricated 1T‐MoS_2_ with a spacing of 9.8 Å aligned vertically on top of graphene using the solvothermal method. A chemical Mo‐O‐C bond between MoS_2_ and graphene generates a rapid transfer path for the charges. Furthermore, the authors verified that the performance of 1T‐MoS_2_ exhibited 666 mAh g^−1^ capacity at a current density of 3500 mA g^−1^. Apart from MoS_2_, phase engineering of other TMDs has been investigated for batteries. For instance, vertically grown 1T‐ReS_2_ exhibited lower performance degradation and improved stability compared with the existing battery. Gao et al.^[^ ^534^ ^]^ reported vertically orientated ReS_2_, particularly using common CVD methods. The researchers placed 1 mg of ReO_3_ powder and 500 mg of sulfur pellet in the middle of a furnace and in the region wrapped with heating tape of upstream, respectively. They realized that sulfur had to be added before the furnace temperature reached 450 °C. This is because sublimation becomes difficult when the temperature exceeds 450 °C as the ReO_3_ powder decomposes into ReO_2_. Subsequently, the amount of Re precursor required to react with the added S element decreased, reducing the number of nucleation sites and inducing a faster growth rate at high temperatures. Consequently, nanosheets were grown on the substrate. The researchers presented vertically grown 1T‐ReS_2_ by optimizing the CVD conditions, which can be used in various applications, including electrocatalysts, batteries, and optoelectronics.

**Figure 26:**
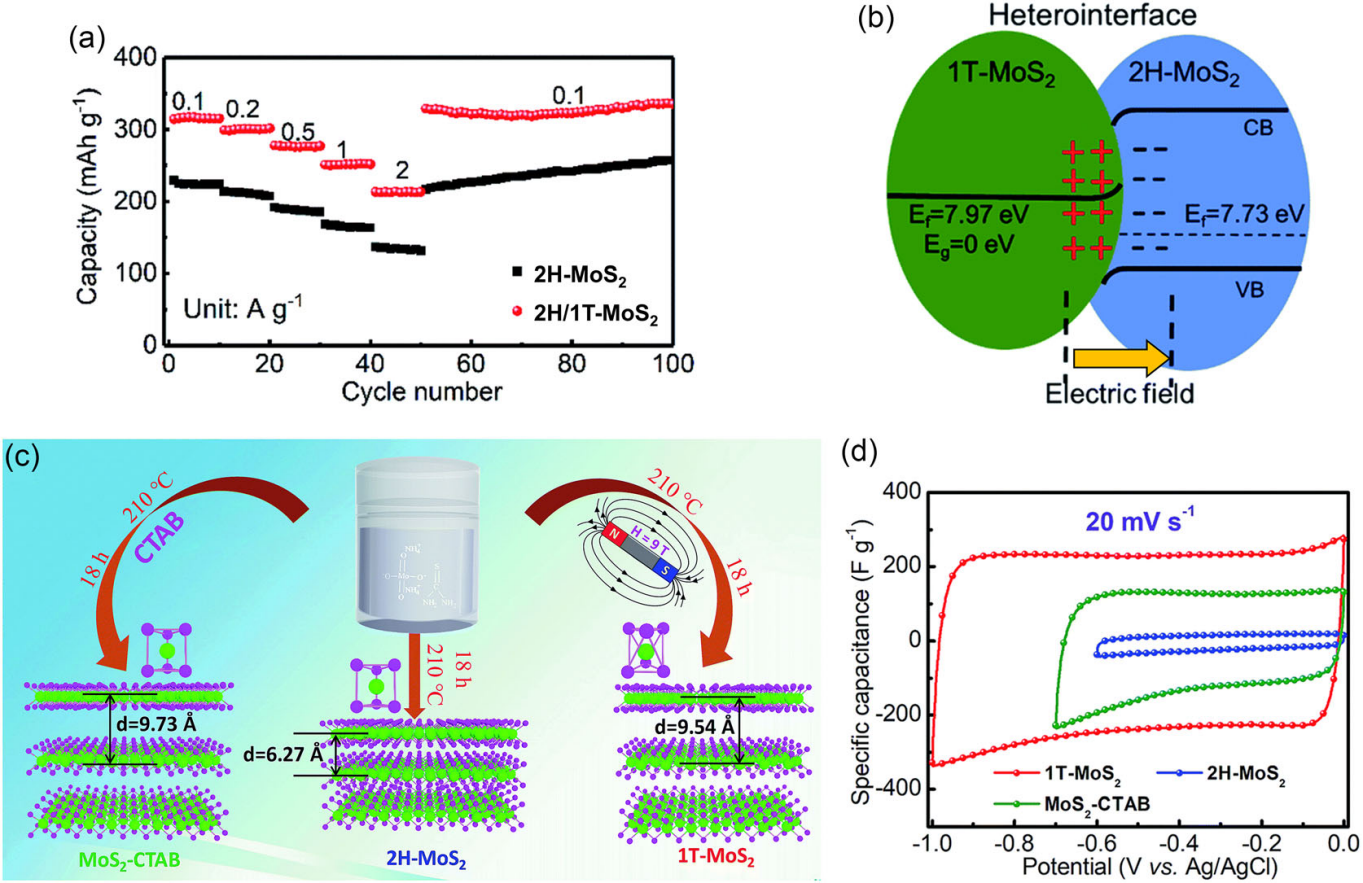
a) Rate capability of 2H‐MoS_2_ (red) and dual‐phase 2H/1T‐MoS_2_ (black).^[^ ^535^ ^]^ b) The electronic band structure in the interface between 1T‐MoS_2_ and 2H‐MoS_2_ inducing the electric field.^[^ ^535^ ^]^ a,b) Reproduced with permission.^[^ ^535^ ^]^ Copyright 2020, Royal Society of Chemistry. c) Schematic of the hydrothermal method used for synthesizing 1T‐MoS_2_ and the spacing distance of each sample, including MoS_2_‐CTAB, 2H‐MoS_2_, and 1T‐MoS_2_.^[^ ^543^ ^]^ d) Cyclic voltammetry (CV) curves of each sample, composed of MoS_2_‐CTAB, 2H‐MoS_2_, and 1T‐MoS_2_ in 1 m Na_2_SO_4_, exhibiting enhanced performance in 1T‐MoS_2_. The scan rate is 20 mV s^−1^.^[^ ^543^ ^]^ c,d) Reproduced with permission.^[^ ^543^ ^]^ Copyright 2019, Royal Society of Chemistry.

In addition to lithium‐ion batteries, sodium‐ion batteries (SIBs) are investigated because they are easily available at low costs and exhibit high specific capacity. Despite the clear advantages, the transport of ions is slow in SIBs. Wu et al.^[^ ^535^ ^]^ circumvented this problem by using dual‐phase 2H/1T‐MoS_2_. Owing to its low conductivity, the intrinsic properties of 2H‐MoS_2_ exhibit an issue when used as the SIB material and is degraded during charging and discharging. However, sodium ions can be easily intercalated easily because the spacing distance in 1T‐MoS_2_ is higher than that in 2H‐MoS_2_. This facilitates ion migration and increases stability, resulting in a reversible capacity of 300 mAh g^−1^ after 200 cycles at a current density of 0.5 A g^−1^ (**Figure** **26**a). Apart from this, certain other reasons exist for using dual‐phase TMDs. First, as the electronic structures of the 2H and 1T phases are different, an electric field is applied to the interface of the heterostructure (Figure 26b). This electric field helps sodium ions move more easily in 2H‐MoS_2_, increasing its overall conductivity. Second, the 2H‐MoS_2_ plays a role in stabilizing the relatively unstable 1T phase, thereby increasing stability. When tested considering a full cell combined with Na_3_V_2_(PO_4_)_3_, a specific capacity of 210 mAh g^−1^ was observed at 0.5 A g^−1^. Geng et al.^[^ ^536^ ^]^ synthesized graphene on a porous 3D structure of Ni foam using CVD, etched the Ni frame, and grew 1T‐MoS_2_ using the hydrothermal method on the remaining graphene. This resulted in a hollow structure with 1T‐MoS_2_ grown both inside and outside the graphene. A reversible capacity of 313 mAh g^−1^ was observed at a current density of 0.05 A g^−1^ after 200 cycles, which was attributed to the increase in the surface area into which sodium ions could enter and the fast transport of ions caused by the 1T phase. Another study explored free‐standing nanotubes based on 1T‐MoS_2_ using the hydrothermal method.^[^ ^537^ ^]^ This 1T‐MoS_2_‐grown hollow nanotube reduces the effect on the volume expansion that occurs with ion intercalation and supplies numerous entry paths for the ions.

When various TMDs are assembled with other two‐dimensional materials, they show unexpected characteristics or properties that cannot be seen in a single two‐dimensional material,^[^ ^538^ ^]^ as exemplified above. Consequently, another different type of phase engineering has emerged based on 2D/2D heterostructures, where the novel architecture and interfaces made from different materials can provide a more desirable structure and properties, expanding the windows of the phase engineering.

So, synergistic combination among materials should be selected, while suppressing the weaknesses of the individual materials for a better electrode structure. Research on MIB(metal ion battery) is drawing attention to solve the problem of 1) scarcity and 2) cyclic stability of Li^+^ and the 3) problem of low theoretical capacity due to the relatively low charge‐to‐mass ratio of monovalent Na^+^.^[^ ^538^ ^]^ However, as ions like Mg^2+^ and Ca^2+^ with a large size and multivalent characteristics of the MIB are charged and discharged, degradation occurs in the conventional battery electrodes.^[^ ^538^ ^]^ So, a 2D heterostructure electrode has been proposed as novel MIB electrode materials that solve this problem. Although TMDs have strengths of a high initial capacity, metal ions larger than Li^+^ have some limitations in the sense of capacity retention and cyclic stability.^[^ ^538^, ^539^ ^]^ Also, despite the high electrical conductivities of TMDs, since they behave as either metal and semiconductor depending on the phase, it is important to control the phase well or overcome the limitations using 2D heterostructure.^[^ ^538^, ^539^ ^]^ For example, an electrode material with high ion conductivity can be developed by combining graphene known to have high electrical conductivity with TMDs capable of achieving high energy density.^[^ ^539^ ^]^ Jiang et al.^[^ ^540^ ^]^ reported enhanced reversible capacity of 1200 mAh g^−1^ with good capacity retention of 95% after 200 cycles of MoS_2_ with graphene. They fabricated MoS_2_‐graphene aerogels to solve problems of restacking and semiconducting MoS_2_. 3D architecture of aerogels prevented MoS_2_ and graphene from being restacked, and the structure and graphene allowed the high electrical conductivity. Jin et al.^[^ ^541^ ^]^ initially synthesized layered TMD(MoS_2_)‐TMO(transition metal oxide, MnO_2_)) using charge balancing cations. With in situ spectroscopic analysis, they identified the efficient interfacial charge transfer of the restacked nanosheets. In addition, according to the results of the DFT calculation, the charge transfer kinetics was improved because stable metallic 1T‐MoS_2_ was obtained as the TMOs was interacting with neighboring TMDs. Therefore, research is underway to improve energy storage performance by selecting various TMDs to create a heterostructure structure, and it is important to study the pathway for selecting 2D heterostructure.

##### Supercapacitors

Supercapacitors can be broadly divided into two types, namely, double‐layer capacitors and pseudocapacitors. In the case of a double‐layer capacitor, the charge is electrostatically stored in the Helmholtz double layer, which is the interface between the electrode and electrolyte. In a pseudocapacitor, energy is stored as a Faradaic electron charge through a chemical reaction, such as a redox reaction. Several studies have explored hybrid capacitors considering both these mechanisms.

Researchers have reported that the 1T phase is favorable for the performance of supercapacitors. Soon et al.^[^ ^542^ ^]^ fabricated a hybrid capacitor with edge‐orientated 1T‐MoS_2_ using thermal evaporation. In terms of double‐layer capacitors, 1T‐MoS_2_ has a larger area than bulk materials to store charges, and the charges can be stored in both inter‐ and intra‐layers. In addition to the double‐layer capacitance, the Mo transition metal may undergo an oxidation reaction to form a Faradaic charge transfer. This 1T‐MoS_2_‐based supercapacitor exhibits a specific capacitance of 100 F g^−1^ at a scan rate of 1 mV s^−1^. Therefore, the edge‐oriented nanowall structure of 1T‐MoS_2_ was verified, opening the possibility for vertically aligned TMDs as enhanced supercapacitors. Wang et al.^[^ ^543^ ^]^ created an air‐stable multilayered 1T‐MoS_2_ using the magneto‐hydrothermal method and compared its performance with that of 2H‐MoS_2_ and MoS_2_‐cetyltrimethylammonium bromide (CTAB). 1T‐MoS_2_ exhibits a high interlayer spacing of 9.5 Å compared with 2H‐MoS_2_, which has a *d*‐spacing of 6.3 Å (Figure 26c); 1T‐MoS_2_ exhibits metallic characteristics and hydrophilic properties as well. The increase in interlayer spacing not only improves the stability during intercalation but also increases the specific capacitance. However, although the interlayer spacing of 2H‐MoS_2_ was increased using the intercalation of CTAB, the specific capacitance was substantially lower than that of the 1T‐MoS_2_. Consequently, the results indicated that the intrinsic conductive nature of 1T‐MoS_2_ contributes to an improved areal capacitance of 320 mF g^−1^ in 1 m Na_2_SO_4_ (Figure 26d). In a follow‐up study,^[^ ^544^ ^]^ 1T‐MoS_2_ was combined with Ti_3_C_2_ MXene nanosheet for the synergy between two materials, wherein the specific capacitance was improved to 386.7 F g^−1^ at 1 A g^−1^. Acerce et al.^[^ ^155^ ^]^ developed an electrode with a supercapacitor that exhibited improved capacitance of 700 F cm^−3^ by restacking 1T‐MoS_2_, which was chemically exfoliated via ion intercalation. The aforementioned analyses verified that 1T‐MoS_2_ has sufficient interlayer spacing for ions to enter during its operation. Moreover, the metallic properties of the 1T phase differ from those of 2H‐MoS_2_, rendering 1T‐MoS_2_ a promising material as an electrode for next‐generation supercapacitors.

Phase‐engineered 1T phase with additional factors, such as defects, can enhance the performance of 1T TMDs. Naz et al.^[^ ^545^ ^]^ fabricated 1T‐MoS_2_ with numerous defects as a supercapacitor electrode using the hydrothermal method. Defective 1T‐MoS_2_ exhibits excellent capacitance of 442 F g^−1^ because the defects, along with the characteristics of 1T‐MoS_2_, increase the reactive sites and provide more paths for ions to pass. A defective 1T‐MoS_2_ can be realized by adjusting the growth time in the hydrothermal method. Reducing the growth time to 6 h resulted in the fabrication of only MoS_2_, with 1T phase of 70% or more; the number of defects was also increased. This improved the electrochemical performance of 1T‐MoS_2_ and eased its use in electrochemical applications.

In summary, the surface area of TMDs should be increased and numerous active sites should be created in terms of phase engineering. Additionally, selecting a metallic or semi‐metallic phase is important for accelerating the movement of charges. This can be advantageous for applying both pseudocapacitor and double‐layer capacitor mechanisms to increase the charge storage in supercapacitors.

#### 7.2.2 Electrocatalysis

The concepts of HER, oxygen reduction reaction (ORR), oxygen evolution reaction (OER), and CO_2_ reduction are the aspects discussed here in terms of electrocatalysis. The number of reactive sites and the movement of electrons are important in chemical reactions. Therefore, improving the conductivity of electrons is extremely essential. In the case of group‐VI TMDs, engineering the phase into metallic 1T or 1T′ phase is considered advantageous.

##### Hydrogen Evolution Reaction (HER)

Hydrogen is a promising candidate as a future energy carrier as it does not generate air pollutants when using fossil fuels. Electrocatalysts can be used to accelerate the reaction and reduce the overpotential.

**Figure 27:**
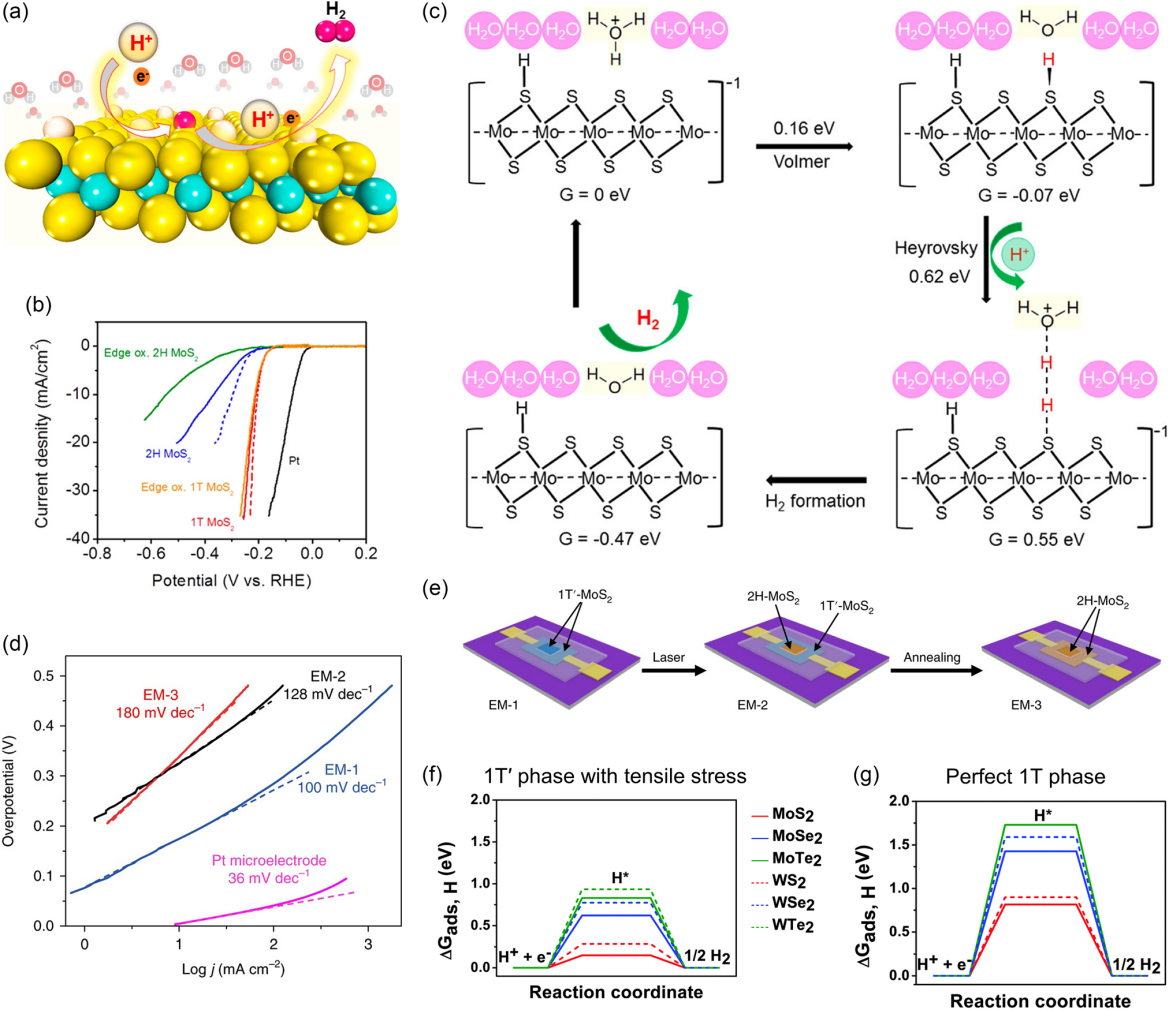
a) Schematic of the HER in 1T‐MoS_2_. Blue, yellow, and red atoms denote molybdenum, sulfur, and hydrogen, respectively.^[^ ^546^ ^]^ b) Polarization curves of 1T‐MoS_2_ and 2H‐MoS_2_ with and without the edge‐oxidation step. Unlike 2H‐MoS_2_, 1T‐MoS_2_ exhibits no significant change owing to edge‐oxidation. The dashed line indicates the *iR*‐corrected curve.^[^ ^549^ ^]^ c) The mechanism of HER in 1T‐MoS_2_. The hydrogen atom is well adsorbed on the sulfur site of the 1T‐MoS_2_ basal plane. Gibbs free energy and activation energy in each step (Volmer step and Heyrovsky step).^[^ ^546^ ^]^ a,c) Reproduced with permission.^[^ ^546^ ^]^ Copyright 2016, American Chemical Society. b) Reproduced with permission.^[^ ^549^ ^]^ Copyright 2013, American Chemical Society. d) Tafel slope and overpotential value of electrochemical microcells, EM‐1, EM‐2, and EM‐3. EM‐1 (1T′‐MoS_2_) exhibits the lowest Tafel slope of 100 mV dec^−1^.^[^ ^553^ ^]^ e) Schematic of the fabrication of each device, namely, EM‐1, EM‐2, and EM‐3.^[^ ^553^ ^]^ d–e) Reproduced under the terms of the CC BY license.^[^ ^553^ ^]^ Copyright 2018, The Authors, published by Springer Nature. f) Calculation of the free energy of hydrogen adsorption (Δ*G*_ads,H_) in the 1T′ phase with tensile stress.^[^ ^554^ ^]^ g) Calculation of the free energy of hydrogen adsorption (Δ*G*_ads,H_) in the perfect 1T phase of various TMDs.^[^ ^554^ ^]^ f,g) Reproduced with permission.^[^ ^554^ ^]^ Copyright 2015, Royal Society of Chemistry.

Before introducing phase engineering in HER applications, the mechanism of HER^[^ ^546^ ^]^ (**Figure** **27**a) should be introduced to understand the reasons for enhanced performance. After undergoing a Volmer step, wherein the proton is adsorbed on the electrode surface by an electrochemical reduction, one of the following two mechanisms occurs. The first possible mechanism is the Tafel step, where two hydrogen atoms adsorbed on the surface react with each other to generate hydrogen. The second possible mechanism is the Heyrovsky step, where a nonadsorbed proton is directly bonded to the adsorbed hydrogen atom. Therefore, as an adsorption/desorption reaction occurs during HER, the HER performance can be evaluated based on the Gibbs free energy of the adsorbed hydrogen.

Certain studies on HER performance using 2H‐MoS_2_ have considered an inert basal plane with the active sites primarily located at edge sites,^[^ ^547^ ^]^ in which the sulfide Mo‐edge is dominant.^[^ ^548^ ^]^

Several other studies have compared 2H‐MoS_2_ and 1T‐MoS_2_.^[^ ^354^ ^]^ Voiry et al.^[^ ^549^ ^]^ confirmed that the electrochemical performance of metallic 1T‐MoS_2_ with the low Tafel slope of 40 mV dec^−1^ is better than that of 2H‐MoS_2_ with a slope of 85 mV dec^−1^. They fabricated 1T‐MoS_2_ by intercalating solvent‐free lithium in a vacuum state. When the edges of 2H‐MoS_2_ and 1T‐MoS_2_ were oxidized, the performance of 2H‐MoS_2_ was degraded, whereas that of 1T‐MoS_2_ remained the same (Figure 27b). This indicated that the active site of 2H‐MoS_2_ is edge dominant, whereas both basal plane and edge site are active in 1T‐MoS_2_. The performance improved further when 2H‐MoS_2_ was combined with the conductive single‐walled carbon nanotubes (SWNTs) to increase conductivity. In their follow‐up study,^[^ ^550^ ^]^ the authors presented the effect of electrical conductivity on HER performance and improved the electrical coupling between the catalysts and substrate with efficient charge injection by reducing the contact resistance through phase engineering. A low‐resistance electrode was fabricated by partially phase‐transforming 1T‐MoS_2_ to 2H‐MoS_2_ and depositing the electrode on the 1T phase. The study reported that sulfur vacancies on the basal plane of 2H‐MoS_2_ can serve as active sites only if the contact resistance is sufficiently reduced to facilitate the charge injection. These studies validate the importance of phase engineering in metallic TMDs considering the advantages of HER catalyst. The 1T phase is not only well bonded with H at the S sites but it also exhibits adequate catalytic reactivity on the basal plane (Figure 27c), causing the active sites in the 1T phase to outnumber those in the 2H phase.^[^ ^546^ ^]^ Additionally, the excellent conductivity of the 1T phase in TMDs and the corresponding efficient charge transfer result in an easier and faster HER. Therefore, 1T‐MoS_2_ has been extensively studied by numerous researchers.^[^ ^139^, ^368^, ^551^, ^552^ ^]^

As the 1T phase of group‐VI TMDs has stability issues, obtaining a stable 1T phase is challenging. Yu et al.^[^ ^553^ ^]^ observed adequate HER performance in the synthesized metallic 1T′‐MoS_2_ in comparison with that of 2H‐MoS_2_. The 1T′ phase comprises a distorted octahedral structure and is more stable than the 1T phase while exhibiting adequate conductivity. The authors enabled the phase transition from 1T′ to 2H through laser irradiation and evaluated HER performance in the basal plane of 1T′‐MoS_2_, 2H‐MoS_2_ with low contact resistance by 1T′‐MoS_2_, and 2H‐MoS_2_ (Figure 27e). The results indicated that 1T′‐MoS_2_ has better HER performance with the onset overpotential of only 76 mV and Tafel slope of 100 mV dec^−1^ when compared with the 2H‐MoS_2_, where the Tafel slope was 180 mV dec^−1^ (Figure 27d). Furthermore, Putungan et al.^[^ ^554^ ^]^ demonstrated the stability and HER performance of 1T′‐MoS_2_, which improved under tensile stress as per the DFT calculations. The researchers determined that the group‐VI TMDs were more stable in the 1T′ phase than in the 1T phase and verified that sulfides were better than selenides or tellurides in terms of hydrogen adsorption. The hydrogen adsorption energies of 1T′‐MoS_2_ and 1T′‐WS_2_ with tensile stress were 0.15 and 0.28 eV, respectively, which were closer to the thermoneutral value than that observed in the 1T phase (Figure 27f,g). Based on these results, the authors suggested that 1T′‐MoS_2_ and 1T′‐WS_2_ are more promising HER catalysts than 2H‐MoS_2_. Furthermore, experiments corresponding to their calculations indicate that 1T′‐MoS_2_ has the potential for HER.^[^ ^549^, ^555^ ^]^

In addition to MoS_2_, studies have analyzed HER with respect to other TMDs.^[^ ^556^, ^557^, ^558^ ^]^ Yang et al.^[^ ^559^ ^]^ identified a favorable HER catalyst with a current density of 5000 mA cm^−2^ by fabricating metallic 2H‐Nb_1+*x*_S_2_ using the added Nb. 2H‐Nb_1+*x*_S_2_ exhibited superior properties in almost all aspects, including current density, overpotential, and Tafel slope, in comparison with other TMDs. Furthermore, researchers have calculated the thermodynamic energy of hydrogen adsorption using DFT analysis. The properties of HER catalyst can be considered adequate when the change is in a thermoneutral range, where the binding energy can be minimized, activating the hydrogen adsorption and hydrogen generation. Theoretical results have shown that the free energy change of 2H‐Nb_1.35_S_2_ is 0.11 eV, which is close to zero. Additionally, the stability and adequate conductivity of the phase improved the HER performance further.

Apart from implementing structural changes, studies have achieved better HER performance with the desired phase via doping^[^ ^560^ ^]^ and engineering defects and strain.^[^ ^554^, ^561^ ^]^ Tang and Jiang^[^ ^546^ ^]^ demonstrated that substitutional doping metals, such as Mn, Cr, Cu, and Ni, can serve as catalysts to enhance HER in 1T‐MoS_2_. The authors focused on V, Cr, Mn, and Fe, which modify the electronic structure by inducing strain without creating structural changes. In the case of Co, Ni, and Fe, the free energy (ΔG_H_) in the process of hydrogen evolution is reduced by doping; however, the bond between metals and catalysts may be excessively strong, which can decelerate the hydrogen production. Conversely, Cr can be used as a suitable dopant because ΔG_H_ is close to zero. Zhang et al.^[^ ^562^ ^]^ used Re‐doping and generated the phase transition in 2H‐MoS_2_ and S vacancies. MoS_2_ doped with Ru exhibits improved HER performance because the generation of phase or S vacancy transforms the electronic band structure. This in turn reduces the barrier between the Volmer step and the intermediate stage of hydrogen adsorption. Therefore, the Ru‐doped MoS_2_ with local phase transition exhibits a low overpotential of 76 mV at 10 mA cm^−2^ in alkaline media.

##### OER, ORR, CO_2_ Reduction

The interest in renewable energy has increased to reduce the use of fossil fuels. Therefore, in addition to HER, studies on OER, ORR, and CO_2_ reduction have also increased. In contrast to HER, when solar energy is stored in the form of O_2_ and H_2_, the process of water splitting into oxygen and hydrogen undergoes four complex chemical reactions that lead to the slow kinetics of OER. Although studies related to MoS_2_ have focused on HER, OER has rarely been considered. German et al.^[^ ^563^ ^]^ determined that it is intrinsically difficult for 1H‐MoS_2_ to act as a catalyst in OER owing to the slow activity caused by the extremely weak bond between two important intermediates (hydroxyl and hydroperoxyl) of MoS_2_. Therefore, in addition to considering the high conductivity and intrinsic activity in an OER catalyst to enable chemical reactions, the binding energy and desorption mechanism should be examined in the intermediate state. Several studies have shown the improvement in OER performance through phase engineering in TMDs other than MoS_2_. Khan et al.^[^ ^564^ ^]^ synthesized composite materials comprising graphite and 1T‐WS_2_ nanodisc using the solvothermal method; they confirmed excellent OER performance based on the photoelectrochemical performance. Combining 1T‐WS_2_ with graphite results in increased solar light absorption and enhanced conductivity that improves electron transfer. Zhang et al.^[^ ^565^ ^]^ reported that 2H‐TaS_2_ nanoflakes are fascinating OER catalysts. 2H‐TaS_2_ on the Ni foam exhibits an extremely low overpotential of 316 mV at 10 mA cm^−2^ and a Tafel slope of 81 mV dec^−1^, indicating that the process is faster when compared to that observed in RuO_2_. Although the Ni foam exhibits poor activity, 2H‐TaS_2_ presents adequate catalytic properties with high activity. Apart from improving charge transfer, 2H‐TaS_2_ remains highly stable in both alkaline and acidic electrolytes. This can be attributed to the superlattice in the single phase, wherein more active sites are exposed.

ORR is an important reaction in determining energy conversion performance. Luxa et al.^[^ ^566^ ^]^ explored the catalytic properties of 2H‐TaS_2_ and 1T‐TaS_2_ through computational analysis. These two phases exhibit poor HER performance yet operate as efficient catalysts in ORR. The authors performed electrochemical treatment of redox reactions, which increased the concentration of Ta^5+^. However, the reduction also created the Ta^0^ state, evidently degradable to Ta^5+^ owing to its unstable properties. The concentration of Ta^5+^ in the reduced TaS_2_ increased, which resulted in improved performance of the ORR catalyst. The aforementioned studies present the strategies for using a low‐cost ORR catalyst with comparable catalytic performance that can replace the existing precious metal catalysts.

As reducing CO_2_ emission has become a critical issue in terms of environmental pollution, converting the emitted CO_2_ to a usable fuel is essential for carbon neutrality. The emitted product may vary depending on the catalyst used and the mechanism of the reaction. Therefore, obtaining the desired products and enhancing the CO_2_ reduction reaction through phase engineering can sufficiently contribute to reducing environmental pollution. Hong et al.^[^ ^567^ ^]^ demonstrated the effect of metal doping on ORR performance based on 2H/1H‐MoS_2_. The linear scaling relationships between CO_2_ reduction intermediates limit the catalytic efficiency. Therefore, stabilizing key intermediates, such as COOH*, CHO*, and COH*, can enhance CO_2_ reduction. Researchers have reported that the bonding of CO* decreases when the sulfur edge of MoS_2_ is doped, resulting in increased adsorption of the key intermediates. The dopant metal reacts with CO* and sulfur reacts with the key intermediates, enhancing the ORR performance with a low overpotential of 0.54 eV. Linghu et al.^[^ ^568^ ^]^ conducted a study with respect to the 1T′ phase and verified that various products can be obtained through CO_2_ reduction by embedding transition metals on 1T′‐MoS_2_. Particularly, 1T′‐MoS_2_ embedded with Ru and Pt can produce CH_4_ and CH_3_OH, respectively, which can be used as energy sources. Similarly, one‐carbon chemistry products, such as CH_4_ and CH_3_OH (except CO), can be produced with high values by modifying with transition metals.

### 7.3 Optoelectronics

Owing to their three‐atomic‐layer structure, TMD nanosheets typically comprise multiple stable phases, wherein the final state can be easily tuned by external factors. Particularly, group‐VI TMDs have attracted significant research interest with respect to nano‐optoelectronics because of their unique optoelectronic properties combined with structural phases. From an engineering perspective, this presents both opportunities and challenges for phase control in optoelectronic applications. For instance, most group‐VI TMDs contain a semiconducting bandgap in the 2H phase. The size of this bandgap is controlled by the number of layers, and the direct‐to‐indirect bandgap transition occurs within the 2H phase. Therefore, TMDs such as MoS_2_ possess tunable light‐emitting properties ranging from visible light to near‐infrared (NIR) wavelength.^[^ ^569^ ^]^ Furthermore, the 1T and 1T′ phases of TMDs are predominantly metallic and semi‐metallic, respectively, which is beneficial for opto‐electronic charge transport. Moreover, the non‐centrosymmetric semiconducting structure in 3R‐MoS_2_ provides advantages in nonlinear optics.^[^ ^165^ ^]^ Therefore, phase engineering to a desired state is a prominent issue that should be addressed for improving the next‐generation optoelectronic applications. In this section, we broadly review the progress reported in light‐emitting devices (LEDs), single‐photon emitters, photodetectors, and photovoltaic cells.

#### 7.3.1 Light‐Emitting Device

A prominent advancement in the field of optoelectronics is the LED, which is an essential component in multiple modern technologies, such as displays, lighting, and sensing. Typically, TMD‐based LEDs are fabricated using a p–n junction with their 2H/1H phase, where the light is emitted by the recombination of holes and electrons. Unlike graphene, most group‐VI TMDs have a direct bandgap in a single layer and exhibit a highly sensitive response to the absorption of light. Moreover, the tunable semiconducting electronic band structure associated with the 2H phase can afford various engineering windows by controlling the electric field, defects, and strain in terms of light emission. Carladous et al.^[^ ^570^ ^]^ reported the light emission from the ultrathin gold films on 2H‐MoS_2_ via electrical excitation and decay along with the radiative path. Moreover, the tunable electrical structure of 2H‐TMD is known to enhance the quantum yield. Typically, researchers have improved the quantum yields by using electrostatic gate bias. Wada et al.^[^ ^571^ ^]^ fabricated the electrolyte‐based light emitter by growing a lateral heterostructure of 2H‐WS_2_/2H‐WSe_2_ using CVD. The fine interface of the heterostructure subjected to strain induces changes in the electronic structure. In this region, electrons selectively interact with the light at K and K′ valley through the valley drift caused by the strain and electric field. In other words, this 2H‐WS_2_/2H‐WSe_2_ heterostructure in a circular polarization‐sensitive device exhibits a polarizability of 10%. Another possible method of enhancing electroluminescence is to alloy semiconductors while modulating their bandgaps in 2H TMDs.^[^ ^572^ ^]^ A TMD alloy with a gradually varying composition with respect to position exhibits the color‐tunable light‐emitting feature. Pu et al.^[^ ^573^ ^]^ used the CVD‐grown single‐layer WS_2_/WSe_2_ alloy, wherein the light‐emission energy ranged from 1.7 to 2.1 eV. Other researchers have reported enhanced light emission in 2H TMDs by controlling the degree of defects. Edelberg et al.^[^ ^574^ ^]^ optimized growth conditions to control intrinsic point defects, such as metal vacancies and chalcogen interstitial defects. They enhanced the quantum yield by reducing the number of point defects in semiconducting TMDs, which can cause nonradiative recombination in excitons.

**Figure 28:**
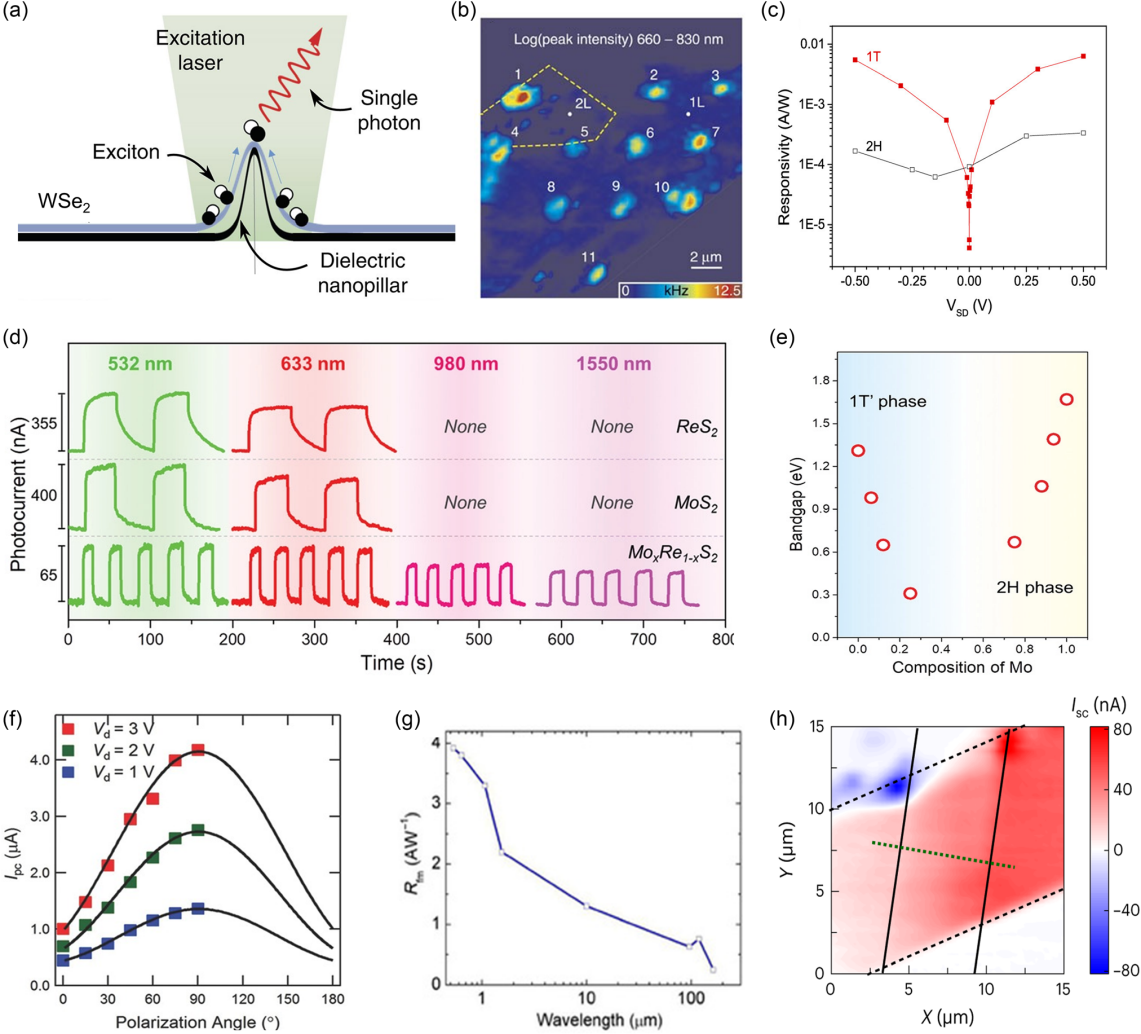
a) Fabrication of the point‐like defect. WSe_2_ nanosheets on nanopillars emit a single photon under the excitation laser.^[^ ^580^ ^]^ b) Mapping photoluminescence peak on WSe_2_ with the peak intensity ranging from 660 to 830 nm.^[^ ^580^ ^]^ a,b) Reproduced under the terms of the CC BY license.^[^ ^580^ ^]^ Copyright 2017, The Authors, published by Springer Nature. c) Photoresponsivity depending on the source–drain bias (*V*_SD_). The comparison of contacts on 1T‐MoS_2_ and 1H/2H‐MoS_2_ indicates enhanced responsivity in 1T‐MoS_2_ contact.^[^ ^582^ ^]^ Reproduced with permission.^[^ ^582^ ^]^ Copyright 2015, American Chemical Society. d) Photocurrent as a function of time in ReS_2_, MoS_2_, and Mo_*x*_Re_1–*x*_S_2_ alloys. Light is illuminated at different wavelengths, including 532, 633, 980, and 1550 nm. V_DS_ is 2 V, V_G_ is 0 V, and the laser density is low at 5 nW mm^−2^. Photoresponse is observed only in Mo_*x*_Re_1–*x*_S_2_ alloys under wavelengths of 980 and 1550 nm.^[^ ^587^ ^]^ e) Bandgap of Mo_*x*_Re_1–*x*_S_2_ alloys as a function of the composition of molybdenum. Electronic band structure can be tuned using composition engineering.^[^ ^587^ ^]^ d,e) Reproduced with permission.^[^ ^587^ ^]^ Copyright 2020, Wiley‐VCH. f) Plots of the photocurrent depending on the polarization angle of the light under different values of drain bias (V_D_).^[^ ^588^ ^]^ Reproduced with permission.^[^ ^588^ ^]^ Copyright 2016, Wiley‐VCH. g) Figure‐of‐merit as a function of wavelength from the visible light to the terahertz range to investigate current responsivity. Wide responsivity in the varying wavelength indicates ultrabroadband photoresponsivity. External bias is 0.72 V, and the light is provided by continuous wave solid‐state lasers (*R*_fm_ = *I*_p_/*P*).^[^ ^589^ ^]^ Reproduced under the terms of the CC BY‐NC 4.0 license.^[^ ^589^ ^]^ Copyright 2018, The Authors, exclusive licensee American Association for the Advancement of Science. h) Spatial distribution of photocurrent in 3R‐MoS_2_ strained along the armchair direction. The light illuminated is 633 nm as per the He–Ne laser with a power of 7 μW.^[^ ^591^ ^]^ Reproduced under the terms of the CC BY license.^[^ ^591^ ^]^ Copyright 2022, The Authors, under exclusive license to Springer Nature Limited.

Single‐photon emitters are recent advancements of traditional LEDs with promising potential in sophisticated and cutting‐edge fields, such as quantum computation and quantum communication. Therefore, several researchers have investigated the applicability of 2H‐TMDs to single‐photon emitters,^[^ ^575^, ^576^, ^577^ ^]^ where a single defect in TMDs can serve as the localized single‐photon sources.^[^ ^578^ ^]^ A single photon is emitted when an exciton recombines near a single‐defect site. The wavelength and intensity of the emitted light can be controlled by the type of excitons and the recombination path, which are tunable with the location of the defect and the local environment. Furthermore, as low‐dimensional semiconducting vdW nanosheets exhibit a high elastic strain limit,^[^ ^579^ ^]^ they present the possibility of controllable quantum confinement with strain. For instance, Branny et al.^[^ ^580^ ^]^ fabricated a quantum emitter in an atomically thin semiconductor using strain engineering at the nanoscale. Nanopillars generate localized nano‐sized strain in single‐layer and bilayer 2H‐WSe_2_ (**Figure** **28**a), which can alter the exciton energy and efficiently concentrate excitons at a unique localized exciton trap site. This results in high‐purity single‐photon emission (Figure 28b). The study revealed that the opto‐electrical properties of TMD nanosheets can be mechanically tailored even in the 2H phase.

#### 7.3.2 Photodetector

A photodetector converts the absorbed light energy into an electrical signal that can either be measured or processed further. Therefore, photodetectors are essential components in numerous electronic devices that require light, such as cameras, optical sensors, and fiber‐optic communication systems. The research on phase‐transformation to 1T/1T′ phase has significantly progressed in terms of metallic and semi‐metallic properties. Wang et al.^[^ ^581^ ^]^ reported different properties between metallic and semiconducting phases of MoS_2_. Metallic 1T′‐MoS_2_ exhibits high responsivity, whereas semiconducting 2H/1H‐MoS_2_ exhibits a high on/off ratio. Yamaguchi et al.^[^ ^582^ ^]^ reported that photocurrent can be significantly enhanced by reducing the Schottky barriers through the phase transformation of 2H/1H‐MoS_2_ into 1T phase under metal electrodes. In 2H/1H‐MoS_2_, maximum photocurrent occurs in the regions of contact. However, the maximum value of photocurrent is observed at the center of the channel region in 1T‐MoS_2_, which can be attributed to the decreased Schottky barrier. Furthermore, researchers have determined that the photoresponsivity of the device can be enhanced by over one order of magnitude (Figure 28c) by manipulating the phase to metallic 1T‐MoS_2_. In addition to reducing the contact resistance, the metallic or semi‐metallic phase of TMDs can be appropriate for NIR detection owing to its zero or small bandgaps. Moreover, mid‐infrared technologies have attracted significant attention with respect to certain applications, such as optical communications, optical sensors, and night‐vision cameras. Kim et al.^[^ ^583^ ^]^ modified the phase of MoTe_2_ by optimizing the growth parameters of metal–organic CVD. The 1T′‐MoTe_2_, with a bandgap smaller than that of the 2H phase, can produce a substantial photocurrent in the NIR region, rendering it a desirable candidate for IR photodetection.

Several studies have demonstrated that the electronic band structure of semiconducting phases can be tuned further based on defect engineering without additional structural phase transitions. Yu et al.^[^ ^584^ ^]^ presented a method for tuning the bandgap in 1T‐PtSe_2_ via Se vacancy defect engineering using Ar plasma treatment. Although 1T‐PtSe_2_ is a stable n‐type semiconductor with an indirect bandgap, Se vacancies decrease the bandgap, ultimately leading to broadband photoresponse in bilayer 1T‐PtSe_2_. Consequently, the device exhibits strong light absorption in the mid‐infrared region. Apart from defects, strain can be used to control the optical response while finely modulating the bandgap in the original phase. The Schottky barrier at the interface of heterojunction devices can be manipulated by exploiting the strain‐induced piezoelectric effect, which can be beneficial for flexible photodetectors. Li et al.^[^ ^585^ ^]^ reported that the piezoresistive and piezoelectric effects occur because of the changes in the bandgap and Schottky barrier, respectively, caused by the induced strain. Additionally, Lin et al.^[^ ^586^ ^]^ determined that CdS mixed‐2H‐WSe_2_ heterojunction can be applied to flexible photodetectors, with significant enhancement in photoresponse under the strain‐induced piezoelectric effect. This flexible photodetector exhibits an increased photocurrent of 0.65 nA and photoresponsivity of 33.4 A W^−1^. This is because the polarization charges at the WSe_2_/CdS interface lead to local energy band bending, accelerating the transport of photoexcited carriers. Deng et al.^[^ ^587^ ^]^ reported another method for driving the phase and electronic band in photodetectors. The authors tuned the phase of the alloy by controlling the relative composition of Mo_*x*_Re_1−*x*_S_2_ via CVD growth. For instance, Mo_0.98_Re_0.02_S_2_ and Mo_0.37_Re_0.63_S_2_ present the 2H and 1T′ phases, respectively, wherein the bandgap is modulated via composition engineering. Increasing the Mo concentration from 0.75 to 1 increases the bandgap from 0.62 to 1.67 eV (Figure 28e). Therefore, the device based on Mo_*x*_Re_1−*x*_S_2_ can generate photocurrent ranging from visible light of 532 nm to NIR light of 1550 nm (Figure 28d). The authors fabricated photodetectors that could interact with the light of the desired wavelength, particularly in the NIR region.

Apart from external factors, such as engineering defects, strain, and alloy, several researchers used intrinsic properties, including specific crystal structure and other unique properties, to enhance the performance of photodetectors. First, anisotropic crystal structures have been considered for light polarization‐sensitive photodetector devices. Liu et al.^[^ ^588^ ^]^ fabricated broadband polarization‐sensitive photodetectors with multilayer ReS_2_. The distinctive anisotropic in‐plane crystal structure of ReS_2_ demonstrated the polarization‐dependent photoresponse, which is consistent with optical absorption anisotropy. Figure 28f illustrates the dependence of absorption on the polarization angle at different photo energies in the ReS_2_ device. The photodetector based on the CDW phase of ReS_2_ manifests n‐type behavior with mobility of ≈40 cm^2 ^V^−1^ s^−1^ and an on/off ratio of 10^5^. The high photoresponsivity of the linear dichroic photodetection highlights the potential of TMD nanosheets with intrinsic anisotropy and presents new possibilities for their utilization in light polarization detection. Wu et al.^[^ ^589^ ^]^ demonstrated that a CDW system of 1T‐TaS_2_ is highly responsive to visible light at terahertz frequencies, resulting in a current response of ≈1 A W^−1^ at room temperature (Figure 28g). The CDWs present in 1T‐TaS_2_ render it an exceptional photodetector that exhibits high sensitivity across an ultrabroadband range, spanning from visible light to the terahertz range.

In addition, the epitaxial growth of SnX (X = S, Se), which have five reported phases, with a controlled phase is in the spotlight. However, it is difficult to synthesize SnX of different phases with the same composition.^[^ ^590^ ^]^ Using PVD, Sheng et al.^[^ ^590^ ^]^ controlled the phase of SnS without change of composition and ultimately achieved optoelectronics with high performance. By reducing the growth temperature and precursor concentration, α‐SnS (*Pbnm*) nanosheets could be phase changed to β‐SnS (*Cmcm*) nanowires. It results from competition between SnS and mica interface bondings and phase cohesive energy. By achieving a phase transition from α‐SnS to β‐SnS, the stability of the SnS nanostructure is improved, and the bandgap is reduced from 1.03 to 0.93 eV. Therefore, it was possible to produce a broadband photodetector with a very low dark current of 21 pA at 1 V and a very fast response speed of ≤14 μs. β‐SnS‐based photodetector showed a maximum detection rate (2.01 × 10^8^ Jones), 1–2 times larger than α‐SnS, showing the potential to improve optoelectronics performance by controlling the phase of Tin chalcogenide nanomaterials.

Therefore, phase engineering provides an essential means of controlling the different performances of photodetectors by manipulating the presence of specific individual phases or the precise combination of multiple phases.

#### 7.3.3 Photovoltaic Cell

A photovoltaic cell, also referred to as a solar cell, is a device that converts the absorbed light into electrical energy. However, unlike photodetectors, photovoltaic cells primarily refer to generating electric power from sunlight or other light sources. Analogous to photodetectors, photovoltaic cells are generally in the form of *p*–*n* diodes, indicating that precise phase‐tuning is important. While the semiconducting phase is used for regulating the wavelength of incident light that interacts with the devices, the metallic phase is generally employed for facilitating the transfer of charge carriers.

Researchers have studied photovoltaic devices whose unique properties emerge from a specific phase. Dong et al.^[^ ^591^ ^]^ observed a bulk piezo‐photovoltaic effect in 3R‐MoS_2_. Multilayer 3R‐MoS_2_ has a non‐centrosymmetric rhombohedral structure, and the bulk photovoltaic effect is enhanced when the strain is applied owing to the strain‐induced polarization. At tensile stress of 0.2%, the photocurrent increases by two orders of magnitude. This polarization‐enhanced bulk photovoltaic effect exhibits unique crystallographic orientation dependence, with an increase in anisotropy along the armchair direction (Figure 28h). Fan et al.^[^ ^592^ ^]^ reported the enhancement of photovoltaic output using 1T‐WS_2_ nanosheets as rear window layers of bifacial perovskite solar cells. They placed the 1T‐WS_2_ in the organic hole transporting layer, indicating that hole transfer is accelerated with the increase in both thickness and transmittance. The reduced interfacial potential of the hybrid hole transfer layer increases the work function, which results in the valance band being close to that of perovskite.

A dye‐sensitized solar cell is a type of photovoltaic device that converts illuminated sunlight into electrical energy. They comprise a semiconductor material, such as titanium dioxide (TiO_2_), coated with a light‐absorbing dye, typically a metal complex or an organic dye, which is capable of absorbing light in the visible light to NIR range. Metallic TMDs can be used for the counter electrode (CE), which requires adequate catalytic activity for the reduction.^[^ ^593^ ^]^ Hussain et al.^[^ ^594^ ^]^ reported that the 1T′ phase of MoTe_2_ grown by sputtering CVD performs excellently as the CE with a high‐power conversion efficiency of 7.25% under a solar light of 100 mW cm^−2^; this was comparable to the CE of Pt (8.15%). Furthermore, researchers have reported that 2H‐WS_2_/1T′‐MoTe2 heterostructure as the CE in dye‐sensitized solar cells can result in an even higher power conversion efficiency of 7.99%.^[^ ^595^ ^]^ This high value suggests that precise phase tuning in heterostructure is necessary for increasing the efficiency of photon‐to‐current conversion.

Another approach to phase engineering TMDs is to alter their chemical composition. Tan et al.^[^ ^596^ ^]^ fabricated the 1T phase of MoS_2*x*_Se_2(1−*x*)_ and Mo_*x*_W_1−*x*_S_2_ with a high yield of 66% via the electrochemical Li‐intercalation method, which was capable of mass production. The thin‐film MoS_2*x*_Se_2(1−*x*)_ was used as a CE, and a power conversion efficiency of 6.5% was observed. Hudie et al.^[^ ^597^ ^]^ also conducted phase engineering of semi‐metallic Mo_*x*_W_1−*x*_Te_2_ nanosheets by controlling the composition. They proposed a method to fabricate semi‐metallic T_d_‐Mo_0.29_W_0.72_Te_1.99_. Here, the T_d_ phase exhibited improved performance with a carrier density of (1.59 ± 0.04) × 10^20^ cm^−3^. The efficiency was 8.85%, which was better than that of sputtered Pt (8.01%) owing to its low charge‐transfer resistance in electrocatalyst reactions.

## 8 Conclusion and Outlook

In this review, we explored the developments reported with respect to vdW materials, specifically focusing on the phase engineering of 2D TMDs. To date, the research on phase engineering in vdW TMD nanosheets has been broadly categorized into two types of strategies, namely, the phase‐conversion and phase‐selective synthesis or treatments. Unlike other bulk materials, vdW materials can easily introduce structural and electrical changes via their unique hetero‐stacking or the twisting between layers. Moreover, additional structural degrees of freedom can be obtained through the polymorphic transition within a layer as the thermodynamics of low‐dimensional nanomaterials are highly vulnerable to external stimuli.

In Section 2, we briefly summarized several representative structural phases available in vdW TMDs and compared their commonalities and differences. Initially, we introduced the 2H/1H and 1T structures of vdW TMDs, including their derivative structures, and explained the relationships among them. The polymorphism of vdW TMD nanosheets can be classified into two types, including different structures in a single‐layer TMD (1H, 1T′, 1T″, and 1T‴) and differently stacked multilayer TMDs (3R, 2H_a_, and T_d_). Using MoS_2_ as a basic example, we outlined the impact of structural diversity on the electronic band structure. The five states of the *d*‐orbital can be split into different energy levels, which are dependent on the p–d hybridization, and vdW interactions, which are influenced by structural distortion in 2H/1H and 1T configurations.

In Section 3, we briefly introduced classical thermodynamics to gain a more intuitive understanding of polymorphic behaviors. Our analysis of existing studies determined that spontaneous phase shifts are characterized by a negative change in Gibbs free energy. Although rapid kinetics of transformation can modify the final state at the nano or atomic scale, Gibbs free energy continues to serve as a convenient approach for estimating the relative stability of different phases. Subsequently, to demonstrate the validity of the suggested interpretation approach, we presented various phase conversions reported with respect to MoS_2_ and applied the concept of free energy to understand the phase switching between 2H/1H and 1T (or 1T′). The examples demonstrate that the activation energy barrier for phase conversion is kinetically influenced by various stimuli, such as atomic position deviation, defects, and electric field.

In Section 4, we reviewed the experimental tools that are used for monitoring the phase behaviors of vdW TMD nanosheets. Thus far, TEM/STEM, GIXRD, Raman spectroscopy, and STM/SPM have been extensively employed to evaluate phase behavior and detect morphological transitions. However, several unresolved issues exist in terms of experimental detection. First, resolving the polymorphic structures of TMD nanosheets can be challenging owing to their overall structural similarity. Therefore, comprehensive elucidation is generally necessary by combining multiple measurements. Second, as several 2D materials are unstable in the open air, their synthesis and characterization are frequently restricted in UHV conditions. This is because the evaluation of their structures primarily relies on UHV‐compatible methods, such as UHV‐STM/SPM. Additionally, despite its potential to exhibit a greater impact on phase behavior compared with bulk materials, the kinetics of 2D phases are not sufficiently explored owing to the fast reaction rate. Therefore, time‐resolved characterization techniques, such as in situ TEM/STEM, in situ XRD, and in situ Raman spectroscopy, are deemed essential. Recently, in situ experiments using aberration‐corrected TEM have provided kinetic information on structural changes driven by various stimuli with high‐spatial resolution and submillisecond time resolution. Along with the structural evolution, the corresponding changes in electronic structure, vibrational spectra, and symmetry‐related properties can be investigated using time‐resolved analytic tools, such as in situ ARPES and SHG. Advancements in time‐resolved studies have facilitated a more dynamic exploration of the kinetics of phase changes in TMD nanosheets.

As the electronic structure and final stable phase are often affected by the atomic coordination and *d*‐electron configuration of transition metals, we classified the TMDs based on the group number of the periodic table for our analysis (Section 5). Furthermore, we briefly reviewed the electronic band properties and structural coordination of TMDs according to this group number. As Mo‐ and W‐based TMDs reveal substantial changes in both structural and electronic properties during phase conversion, they have been subjected to extensive investigation and application. Therefore, we focused on reviewing the advancements in phase engineering specifically in group‐VI TMDs, where the energy difference between 1H and 1T (or 1T′) decreases with the increasing period of the chalcogen atom, whereas the energy difference between 1T and 1T′ increases monotonically. Such trends can be interpreted by the variations in p–d hybridization and work function.^[^ ^87^, ^99^ ^]^ Therefore, although the energy barrier required to activate phase transition is relatively high for MoS_2_, it remains sufficiently low for MoTe_2_. Specifically, MoTe_2_ and WTe_2_ exhibit wide engineering margins and intriguing properties owing to their rich polymorphism, facilitated by the low energy barrier.

In Section 6, we reviewed various phase engineering strategies involved in both phase‐conversion and phase‐selective synthesis using MoTe_2_ as the primary example owing to its low energy barrier. As a representative direct synthesis method, CVD‐based phase regulation of TMDs has been primarily considered with respect to MoTe_2_ and its related alloys. Critical growth factors associated with phase selectivity (composition, precursor, temperature, substrate, and atmosphere) were reviewed to gain an overall understanding of the related thermodynamics and kinetics with potential application to other TMDs. Additionally, we reviewed various post‐growth treatments for phase engineering, including ion intercalation, electric field applications, chemical doping, strain application, and thermal treatment.

Over the years, studies have reported the occurrence of the CDW phase in TMDs; however, certain disputes exist in terms of the mechanisms of their formation. We determined that vdW TMD nanosheets can provide a convenien*t* testing platform for CDW analysis. Therefore, we briefly summarized CDW in vdW TMD nanosheets in Section 7, particularly based on group‐4 transition metals, such as Ta, Nb, and V. The occurrence of the CDW phase can be attributed to several fundamental models, including Fermi surface nesting, electron–phonon coupling, and exciton condensation. Additionally, we presented examples that demonstrate representative CDW behaviors observed using various experimental techniques. We also discussed the method for controlling the CDW phase state through pressure, element doping, and dimensional reduction.

Finally, in Section 8, we reviewed the prospective research directions in the field of phase engineering and discussed their potential applications. Owing to the ability of phase engineering to modulate the structural symmetry and electronic band structure, TMDs have demonstrated increased adaptability in novel electronic devices. This can be attributed to the high carrier mobility, high on/off ratios, low contact resistance, and memory properties of TMDs. Additionally, phase engineering can modulate the bandgap structure over a relatively wide range, thereby expanding the applicability of TMDs to the optoelectrical fields, where the wavelength of the light can be controlled by appropriate phase tuning. This tunable band structure and electrical properties of TMDs render them ideal for use in LEDs, photodetectors, and solar cells. Furthermore, certain TMDs exhibit exotic intrinsic properties, such as anisotropic structures or CDW systems, which enable the design of unique devices with polarization‐sensitive properties or broadband photoresponsivity, respectively. In electrochemical applications, the phase conversion to metallic 1T/1T′ or the increase in the number of active sites in the 2H phase via defect or strain engineering can significantly impact the reaction mechanism and charge transfer. Therefore, precise control of TMD phases can result in electrochemically active materials. In this article, we discussed the application of TMDs in batteries, supercapacitors, and electrocatalysis for HER, ORR, OER, and CO_2_ reduction. As the charge transfer is crucial in these fields, metallic TMDs are primarily used for enhancing the transport rate and dual phase has been extensively studied for the same purpose.

Thus far, the precise control of phase in TMDs has shown its potential in various applications in the fields of electronics, optoelectronics, and electrochemistry. Despite its promising possibility in diverse fields, certain challenges must be overcome before advancing the use of TMDs in practical applications. First, the difficulties in accurately controlling and manipulating the phases of TMDs should be addressed. As the process of phase engineering often requires delicate, complex, and expensive manufacturing techniques, moving to the stage of industrial applications may not be easy until the aforementioned challenges are circumvented. Second, the low stability of the engineered phase is a problem. Certain phases, primarily the 1T phase of group‐VI TMDs, tend to spontaneously revert to a more stable phase under ambient environmental conditions, resulting in degraded durability, stability, and longevity. Finally, researchers should identify synthesis and control methods that are compatible with the conventional method for creating phase‐engineered TMDs on a mass‐production scale. Therefore, to use phase‐engineered TMDs in various applications, the aforementioned problems must be overcome based on further research and development.

## Conflict of Interest

The authors declare no conflict of interest.
